# Reduced adipocyte glutaminase activity promotes energy expenditure and metabolic health

**DOI:** 10.1038/s42255-024-01083-y

**Published:** 2024-07-15

**Authors:** Simon Lecoutre, Salwan Maqdasy, David Rizo-Roca, Gianluca Renzi, Ivan Vlassakev, Lynn M. Alaeddine, Romane Higos, Jutta Jalkanen, Jiawei Zhong, Danae S. Zareifi, Scott Frendo-Cumbo, Lucas Massier, Ondrej Hodek, Marta Juvany, Thomas Moritz, Thais de Castro Barbosa, Muhmmad Omar-Hmeadi, Marta López-Yus, Fatiha Merabtene, Jimon Boniface Abatan, Geneviève Marcelin, Elie-Julien El Hachem, Christine Rouault, Martin O. Bergo, Paul Petrus, Juleen R. Zierath, Karine Clément, Anna Krook, Niklas Mejhert, Mikael Rydén

**Affiliations:** 1 Department of Medicine (Huddinge), Karolinska Institutet, ME Endokrinologi, Karolinska University Hospital Huddinge, Huddinge, Sweden; 2 Nutrition and Obesities: Systemic Approaches Research Group, NutriOmics, Sorbonne Université, INSERM, Paris, France; 3 Department of Physiology and Pharmacology, Karolinska Institutet, Stockholm, Sweden; 4 Swedish Metabolomics Centre, Department of Forest Genetics and Plant Physiology, Swedish University of Agricultural Sciences, Umeå, Sweden; 5 The Novo Nordisk Foundation Centre for Basic Metabolic Research, Faculty of Health and Medical Sciences, University of Copenhagen, Copenhagen, Denmark; 6 Adipocyte and Fat Biology Laboratory (AdipoFat), Translational Research Unit, University Hospital Miguel Servet, Zaragoza, Spain; 7 Instituto Aragonés de Ciencias de La Salud (IACS), Zaragoza, Spain; 8 Instituto de Investigación Sanitaria (IIS)-Aragón, Zaragoza, Spain; 9 Department of Biosciences and Nutrition, Karolinska Institutet, Huddinge, Sweden; 10 Department of Molecular Medicine and Surgery, Karolinska Institutet, Stockholm, Sweden; 11 Nutrition Department, Assistance Publique Hôpitaux de Paris, CRNH Ile-de-France, Pitié-Salpêtrière Hospital, Paris, France

**Keywords:** Obesity, Fat metabolism

## Abstract

Glutamine and glutamate are interconverted by several enzymes and alterations in this metabolic cycle are linked to cardiometabolic traits. Herein, we show that obesity-associated insulin resistance is characterized by decreased plasma and white adipose tissue glutamine-to-glutamate ratios. We couple these stoichiometric changes to perturbed fat cell glutaminase and glutamine synthase messenger RNA and protein abundance, which together promote glutaminolysis. In human white adipocytes, reductions in glutaminase activity promote aerobic glycolysis and mitochondrial oxidative capacity via increases in hypoxia-inducible factor 1α abundance, lactate levels and p38 mitogen-activated protein kinase signalling. Systemic glutaminase inhibition in male and female mice, or genetically in adipocytes of male mice, triggers the activation of thermogenic gene programs in inguinal adipocytes. Consequently, the knockout mice display higher energy expenditure and improved glucose tolerance compared to control littermates, even under high-fat diet conditions. Altogether, our findings highlight white adipocyte glutamine turnover as an important determinant of energy expenditure and metabolic health.

## Abstract (web summary)

Lecoutre, Maqdasy and Rizo-Roca show that whole-body pharmacological inhibition or adipocyte-specific deletion of glutaminase in mice activates thermogenesis in inguinal adipocytes and promotes metabolic health. They also link decreased plasma and adipose tissue glutamine-to-glutamate ratios to insulin resistance in humans with obesity.

## Main

Obesity-associated insulin resistance and type 2 diabetes are characterized by changes in the circulating levels of multiple amino acids^1–4^. Targeted restorations in some of these metabolites improve metabolic health, indicating that they are more than mere biomarkers of disease. For example, brown adipose tissue (BAT) activation in mice increases the consumption of branched-chain amino acids and thereby protects against their insulin resistance-promoting effects^5,6^. In humans living with obesity and insulin resistance, several amino acids and their derivatives are altered in white adipose tissue (WAT)^7^. However, the mechanism(s) by which disturbances in white adipocyte amino acid metabolism contribute to metabolic disorders is not fully understood.

Glutamine and glutamate are essential components in numerous cellular processes, encompassing nucleotide and glutathione biosynthesis, tricarboxylic acid cycle (TCA) anaplerosis and transcriptional regulation via epigenetic modifications^8^. These polar amino acids are interconverted by multiple enzymes and their respective levels serve as biomarkers of type 2 diabetes risk, where a high plasma glutamine-to-glutamate ratio is protective in both humans and mice^9,10^. In healthy participants, WAT is an important contributor to this circulating ratio through the release of glutamine and the uptake of glutamate^11^. It is therefore conceivable that perturbations in WAT glutamine and glutamate turnover induced by obesity may contribute to the development of metabolic disorders.

Herein, we investigated how white adipocyte glutamine metabolism affects metabolic health. By combining clinical and experimental studies, we demonstrate that glutamine to glutamate conversion (glutaminolysis) in white adipocytes is increased in people with versus without obesity and insulin resistance. Genetic reduction and pharmacologic inhibition of glutaminase (GLS) in white adipocytes increases the glutamine-to-glutamate ratio and improves glucose tolerance in vivo by rewiring intracellular metabolism and elevating energy expenditure.

## Results

### Glutamine-to-glutamate ratios are linked to insulin resistance

**Fig. 1:**
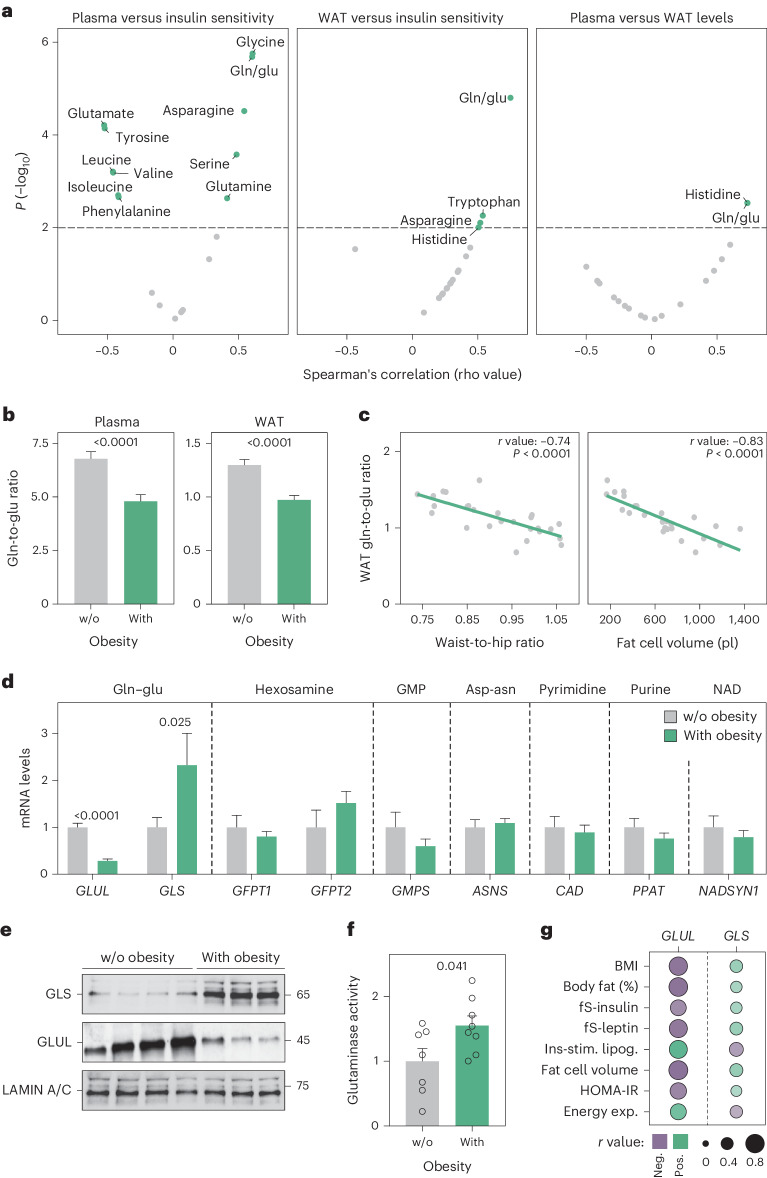
Adipose glutaminolysis is increased in obesity-induced insulin resistance. **a**, Correlations between amino acid levels in plasma and insulin sensitivity (left), amino acid levels in WAT and insulin sensitivity (middle) and between amino acid levels in plasma (*n* = 53) and WAT (*n* = 26) (right) in cohort 1. Insulin sensitivity was measured by hyperinsulinaemic euglycaemic clamp expressed as glucose disposal rate corrected for mean plasma insulin levels at steady state (M/I). Associations were calculated using Spearman’s rank correlation. **b**, The plasma (*n* = 53) (left) and WAT (*n* = 26) (right) glutamine-to-glutamate (gln to glu) ratios in cohort 1 comparing women living without (w/o) or with obesity (BMI ≥ 30 kg m^−2^). Groups were compared using Student’s *t*-test. **c**, The correlation between WAT glutamine-to-glutamate ratios and waist-to-hip ratio (left) or fat cell volume (right) in cohort 1 (*n* = 26). Pearson’s correlation coefficients are shown. **d**, Expression of genes encoding glutamine–glutamate (gln–glu) metabolizing proteins measured by qPCR in isolated fat cells from cohort 2. Results are displayed according to biological pathways and compared for people living w/o (*n* = 12) or with (*n* = 16) obesity. Groups were compared using Student’s *t*-test or the Mann–Whitney *U-*test, depending on the distribution. **e**, Protein levels of GLS and GLUL in subcutaneous white adipocytes from participants living w/o (*n* = 4) or with (*n* = 3) obesity (cohort 2). Proteins derived from the same samples were loaded on two different gels. **f**, GLS activity in subcutaneous adipocytes from participants living w/o (*n* = 7) or with (*n* = 8) obesity in cohort 2. Groups were compared using Student’s *t*-test. **g**, Correlations between indicated clinical and adipocyte parameters and *GLUL* or *GLS* expression in bulk transcriptomic data of subcutaneous WAT from cohort 3 (*n* = 56). All correlations were significant (*P* < 0.05), circle sizes are proportional to the Pearson’s correlation coefficient and the colour indicates positive (pos.) (green) or negative (neg.) (purple) associations. Data in **b**, **d** and **f** show mean ± s.e.m. Relevant *P* values are shown. asp-asn, aspartate to asparagine conversion; energy exp., energy expenditure by indirect calorimetry corrected for body weight (kcal kg^−1^ day^−1^); GMP, guanosine monophosphate; ins-stim. lipog., log_10_ maximal insulin-stimulated lipogenesis in isolated mature fat cells. Source data

**Extended Data Fig. 1:**
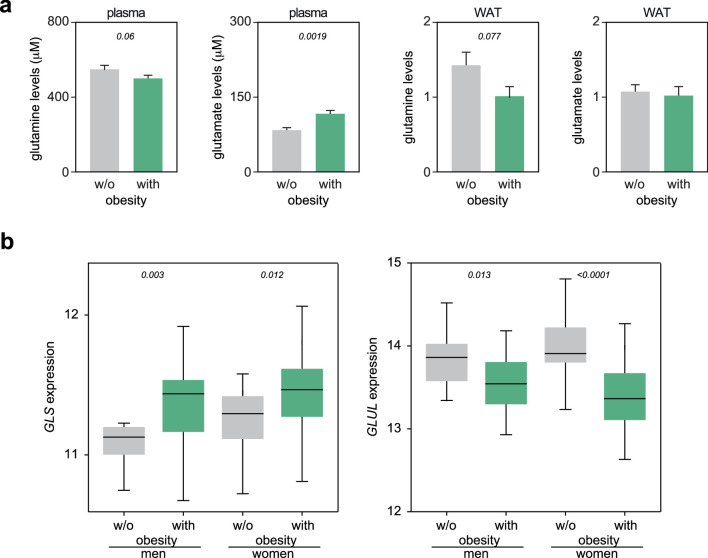
Glutamine turnover is altered in WAT of men and women with obesity. **a**. Plasma (n = 53, the two panels to the left) and subcutaneous white adipose tissue (n = 26, the two panels to the right) glutamine and glutamate levels compared between individuals living with or without (w/o) obesity. The log values of glutamine levels in WAT and plasma have previously been published in^7^. Data were analyzed by Student’s *t*-test or the Mann-Whitney U test, depending on the distribution, and *p* values are shown. Error bars are S.E.M. **b**. Results from publicly available RNA-seq data (GSE95640)^14^ summarizing the expression of *GLS* (left panel) and *GLUL* (right panel) in subcutaneous white adipose tissue from men living with (n = 55) or w/o obesity (n = 10) and women living with (n = 101) or w/o (n = 14) obesity. Data were analyzed by Wilcoxon rank sum test and *p* values are shown. Box plots display min, max, and median. Source data

We set out to systematically determine the relationship between obesity-induced insulin resistance and glutamine-to-glutamate stoichiometry in the circulation and WAT. For this, we collected plasma and subcutaneous WAT samples from women with and without obesity exhibiting different degrees of insulin sensitivity as determined by hyperinsulinaemic euglycemic clamps (clinical characteristics are provided in Supplementary Table 1). As expected, our mass spectrometry-based analyses revealed that plasma branched-chain (valine, leucine and isoleucine) and aromatic (tyrosine and phenylalanine) amino acids were negatively associated with glucose disposal rate, while serine, asparagine and glycine displayed positive correlations (Fig. 1a)^2,12^. These findings demonstrate that our measures are accurate, and that the cohort recapitulates known biology. By mining the data further, we noticed that the circulating glutamine and glutamate levels displayed reciprocal relationships where the glutamine-to-glutamate ratio was strongly associated with insulin sensitivity (Fig. 1a). When comparing with the corresponding levels in WAT, we found the glutamine-to-glutamate ratio in the two compartments to be closely correlated (Fig. 1a). Furthermore, in women with versus without obesity, the plasma and WAT glutamine-to-glutamate ratios were decreased, and the latter was associated with waist-to-hip ratio and fat cell volume: two independent measures of metabolic health (Fig. 1b,c and Extended Data Fig. 1a). Altogether, our results indicate that the subcutaneous WAT glutamine-to-glutamate ratio reflects circulating levels and is linked to alterations in fat mass distribution and insulin sensitivity.

### *GLS* and *GLUL* levels associate with insulin resistance and energy expenditure

**Extended Data Fig. 2:**
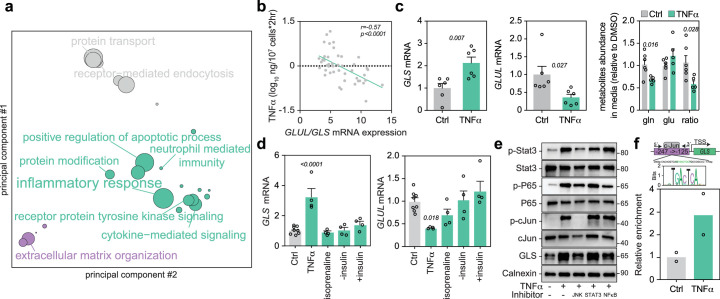
TNFα promotes glutaminolysis in white adipocytes. **a**. Pathway analysis of genes positively associated with *GLS* mRNA expression and negatively associated with *GLUL* mRNA expression. The size of the dots is proportional to the Gene Set Enrichment Analysis score. **b**. Correlation between *GLUL/GLS* mRNA expression and TNFα secretion *ex vivo* in human subcutaneous adipose tissue in cohort 3 (n = 45). r- and *p* values are shown for correlation using simple Pearson´s regression analysis. **c**. Freshly isolated human adipocytes were cultured with or without TNFα for 24 hrs. Analyses of gene expression by qPCR (six replicates per condition, repeated >three times) (left) and glutamine and glutamate levels including glutamine-to-glutamate ratio (six and five replicates per condition) in conditioned media (right) were performed. Data were analyzed by Student’s *t*-test and *p* values are shown. **d**. Analyses of gene expression by qPCR in human adipocytes incubated with vehicle (n = 8), TNFα (n = 4), isoprenaline (n = 4) or insulin (n = 4). Data were compared using one-way ANOVA with Dunnett’s post-hoc test and *p* values are shown (experiment repeated >three times). **e**. Representative examples of protein levels of GLS and the inflammatory pathways induced by TNFα (JNK, STAT3 and NFκB) in human adipocytes incubated with TNFα for four hours with or without inhibitors of each pathway (repeated two times). Proteins from the same samples were loaded on three different gels in parallel. **f**. Chromatin immunoprecipitation followed by qPCR analysis showing that c-Jun binds to the human *GLS* promoter. Complexes containing c-Jun were immunoprecipitated from cross-linked and digested chromatin isolated from human adipocytes incubated with PBS or TNFα. Following reversal of cross-links and purification of DNA, qPCR was run using primers designed to amplify a 196-bp fragment centered around the putative c-Jun binding site at position −247 to −125 bp relative to the transcriptional start site (TSS). The data presented are the relative quantity values from independent duplicate reactions. Data in panels **c-d** show mean ± S.E.M. **Abbreviations:** TNFα = Tumor necrosis factor alpha, TG = triglycerides, -insulin = cells deprived from insulin for 24 hours, +insulin = insulin treatment for six hours, isoprenaline = isoprenaline treatment for six hours. Source data

The glutamine-to-glutamate ratio in WAT could either reflect plasma concentrations or altered metabolism of these amino acids directly within the tissue. As the interconversion of glutamine and glutamate is controlled by several enzymes^13^, we determined whether genes encoding proteins involved in this turnover were altered in the obese, insulin resistant state. Quantitative PCR (qPCR) measurements based on isolated mature human fat cells from 22 women and six men (clinical characteristics in Supplementary Table 1), revealed that glutamine synthase (*GLUL*) was lower and *GLS* was higher comparing cells from participants with versus without obesity (Fig. 1d). Given that the levels of the other investigated genes were unaffected, this alteration in stoichiometry was unique and suggested increased glutaminolysis in people living with obesity. This notion was corroborated (1) in a replication cohort comprising men and women^14^ (Extended Data Fig. 1b), (2) at the protein level by western blot (Fig. 1e) and (3) with enzyme activity assays (Fig. 1f). To validate these findings further, we measured *GLS* and *GLUL* messenger RNA (mRNA) expression in subcutaneous WAT from women displaying a broad range in body weight and insulin sensitivity^15^. We found inverse associations between these genes and multiple clinical parameters including body mass index, energy expenditure and insulin sensitivity at the whole-body and fat cell level (Fig. 1g). Altogether, this suggests that obesity-induced increases in WAT glutaminolysis are linked to attenuated energy expenditure and insulin sensitivity.

### TNF promotes *GLS* expression via the JNK-c-Jun pathway

We next set out to identify factors that regulate *GLS* and *GLUL* expression in fat cells. For this, we first identified pathways correlating with the *GLUL*-to-*GLS* ratio in human WAT from 56 women with or without obesity^15^. Our analyses revealed pro-inflammatory processes to be highly enriched (Extended Data Fig. 2a). By measuring secretion of the pro-inflammatory cytokine TNF from WAT pieces in the same cohort, we confirmed a negative correlation between the *GLUL*-to-*GLS* ratio and TNF levels (Extended Data Fig. 2b). To test for causality, we incubated adipocytes in the presence or absence of recombinant TNF. Compared to non-stimulated cells, TNF treatment increased *GLS* and concomitantly reduced *GLUL* mRNA levels, changes that were paralleled by a reduction of the glutamine-to-glutamate ratio (Extended Data Fig. 2c). Given that TNF increases lipolysis^16^, we also assessed the effects of two other regulators of lipid turnover, insulin and isoprenaline, on *GLS* and *GLUL* expression. However, incubations with these factors did not affect the mRNA levels of the two genes indicating that the effects of TNF were independent of its action on lipid metabolism (Extended Data Fig. 2d). Next, we dissected the intracellular signalling events linking TNF to glutamine metabolism. As shown in Extended Data Fig. 2e, incubation with inhibitors targeting signal transducer and activator of transcription 3 (STAT3), nuclear factor-κB and c-Jun N-terminal kinase (JNK), revealed that only the latter abrogated the effects of TNF on GLS protein levels. In human breast cancer cells, JNK has been shown to regulate glutaminolysis by promoting binding of the transcription factor c-Jun to the promoter region of *GLS*^17^. We therefore performed chromatin immunoprecipitation followed by qPCR and observed an enrichment of c-Jun in the *GLS* promoter following TNF stimulation (Extended Data Fig. 2f). Taken together, we show that TNF transcriptionally regulates *GLS* levels through the JNK-c-Jun pathway.

### Glutaminolysis perturbations alter energy metabolism gene sets

**Fig. 2:**
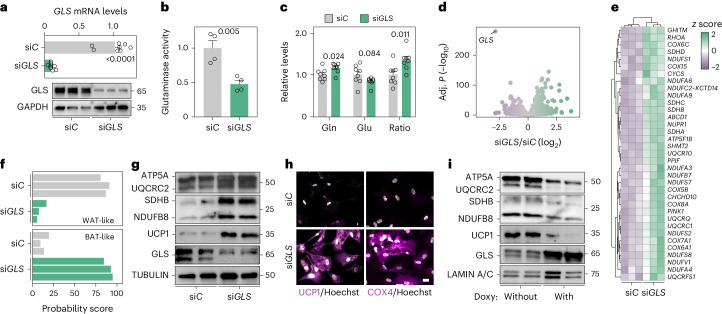
Reduced GLS activity promotes thermogenic gene expression in adipocytes. **a**, GLS mRNA and protein levels in human adipocytes transfected with non-silencing (si*C*) or GLS-targeting (si*GLS*) oligonucleotides. Data for mRNA are expressed relative to si*C* and were compared using Student’s *t*-test (eight replicates per condition, repeated more than three times). A representative western blot of GLS protein is shown (repeated more than three times). GAPDH was used as a loading control. **b**,**c**, Comparisons of si*C* and si*GLS* adipocytes displaying GLS enzyme activity (four replicates per condition, repeated more than three times) (**b**) and glutamine (gln) and glutamate (glu) levels including glutamine-to-glutamate ratio (eight replicates per condition, repeated more than three times) (**c**). Data were compared using Student’s *t*-test. **d**,**e**, RNA sequencing data comparing si*C* and si*GLS*-treated human adipocytes (three replicates per condition). Data are presented as a volcano plot of all genes (**d**) and a heatmap of changes in the expression of cellular respiration genes (**e**). **f**, Probability of BAT- or WAT-like transcriptomic profiles in human adipocytes transfected with si*C* or si*GLS* oligonucleotides. The score is based on ProFAT^18^, which quantifies the thermogenic potential from gene expression datasets. **g**, ETC, uncoupling protein 1 (UCP1) and GLS protein levels in human adipocytes transfected with si*C* or si*GLS* oligonucleotides (repeated more than three times). Proteins from the same experiment were loaded on three different gels run in parallel. **h**, Representative images of UCP1 and cytochrome C oxidase (COX4) immunofluorescence in human adipocytes transfected with si*C* or si*GLS* oligonucleotides (repeated three times). Scale bar, 20 μm. **i**, Representative examples of GLS, ETC, UCP1 and LAMIN A/C protein levels in human adipocytes engineered to overexpress *GLS* on doxycycline (doxy) incubation. Data show comparisons of non-induced versus induced cells (repeated twice). Data **a**–**c** show mean ± s.e.m. Relevant *P* values are shown. Source data

**Extended Data Fig. 3:**
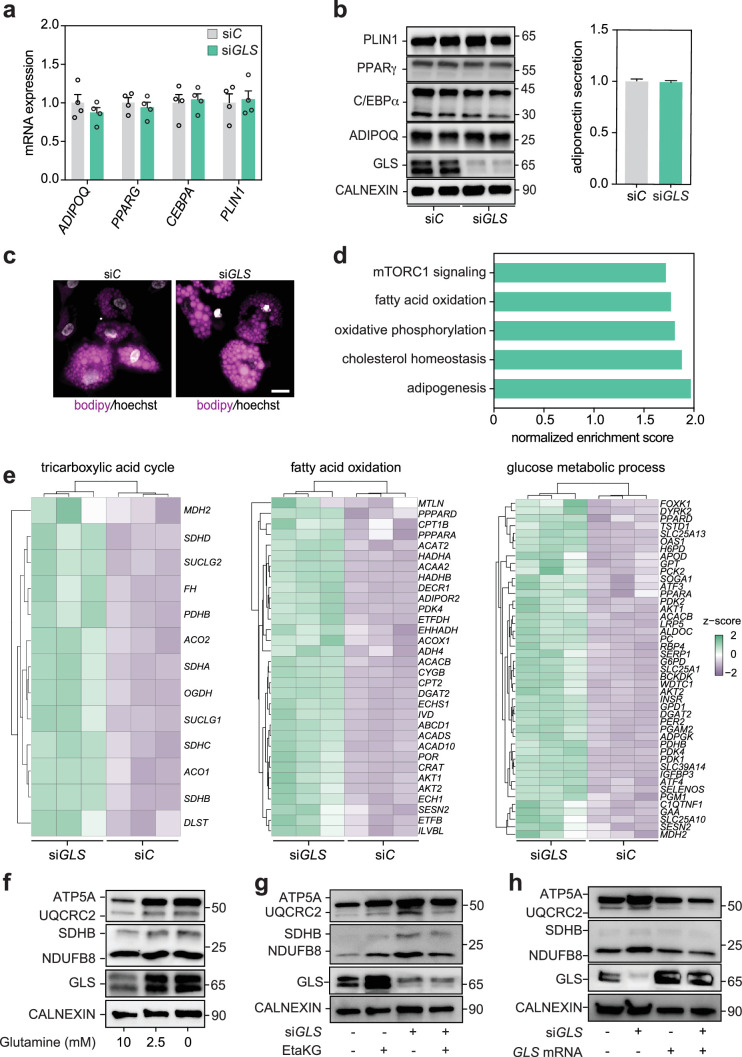
GLS depletion promotes metabolic gene programs independently of effects on adipogenesis. **a-c**. *In vitro* differentiated human adipocytes were transfected with si*C* or si*GLS*. Effects on (**a**) gene expression (four replicates per condition, repeated >three times), (**b**) protein abundance (repeated twice) of adipogenic markers (left) and adiponectin secretion (in total sixteen replicates per condition from two independent experiments) (right) and (**c**) lipid droplet morphology (repeated >three times) were determined. Scale bar in panel **c** = 20 μm. Proteins from the same samples were loaded on four different gels in parallel. **d**. Gene set enrichment analysis listing the top five pathways altered in si*GLS vs*. si*C* human adipocytes. **e**. Heatmaps of indicated pathways and genes altered in si*GLS vs*. si*C* treated human adipocytes. **f**. Representative examples of electron transport chain (ETC) protein levels in human adipocytes incubated with 0, 2.5 and 10 mmol/L of glutamine. Cells were either deprived or treated with indicated concentrations of glutamine for 48 hours (repeated three times). Proteins from the same samples were loaded on two different gels in parallel. **g**. Representative examples of ETC protein levels in human adipocytes transfected with si*C* or si*GLS* oligonucleotides incubated with or without eta-ketoglutarate (EtaKG, repeated three times). **h**. Representative examples of ETC protein levels in human adipocytes transfected with si*C* or si*GLS* oligonucleotides and with scrambled mRNA or over expressing *GLS* mRNA. (repeated two times). Error bars in panels **a-b** are S.E.M. **Abbreviations**: ADIPOQ = adiponectin, BODIPY = boron-dipyrromethene, CEBPA = CCAAT/enhancer-binding protein alpha, PLIN1 = Perilipin 1, PPARG = peroxisome proliferator-activated receptor gamma. Source data

To understand how altered glutamine turnover affects white fat cell function, we targeted *GLS*, which encodes the rate-limiting enzyme in glutaminolysis^8^. By electroporating human adipocytes with small-interfering RNA (siRNA) duplexes, we efficiently reduced GLS mRNA and protein levels (Fig. 2a), resulting in decreased GLS activity (Fig. 2b) and increased glutamine-to-glutamate ratio (Fig. 2c). These effects were not secondary to alterations in adipogenesis as the treatment did not affect adipocyte marker mRNA and protein levels, endocrine function or lipid content (Extended Data Fig. 3a–c). By performing RNA sequencing and pathway analyses in *GLS*-depleted versus control adipocytes, we found no clear patterns among downregulated genes. However, multiple metabolic gene sets, for example, oxidative phosphorylation, TCA cycle and fatty acid oxidation, were upregulated in si*GLS* adipocytes (Fig. 2d,e and Extended Data Fig. 3d,e). To analyse our data in more detail, we used ProFAT that quantifies the thermogenic potential from transcriptomic data^18^. This revealed that *GLS* depletion increased the expression of genes linked to thermogenesis and substrate use (Fig. 2f), including components of the electron transport chain (ETC) and uncoupling protein 1 (UCP1), effects that were also observed at the protein level (Fig. 2g,h). To validate and extend these results, we used several orthogonal approaches. First, we observed effects on ETC protein levels by altering glutamine concentrations in the media (Extended Data Fig. 3f). Second, in rescue experiments, we reversed the si*GLS* phenotype by incubating the cells with eta-ketoglutarate (a cell-permeable analogue of the glutamate derivative α-ketoglutarate to restore TCA anaplerosis) or by re-expressing GLS (Extended Data Fig. 3g–h). Third, we generated human adipocytes with doxycycline-inducible *GLS* expression. In these cells, incubations with doxycycline resulted in marked *GLS* induction with concomitant reductions in UCP1 and ETC proteins (Fig. 2i). Collectively, this suggests that perturbations of human adipocyte glutaminolysis affect the expression of genes involved in substrate use and bioenergetic pathways.

### Reduced GLS activity increases oxidative metabolism

**Fig. 3:**
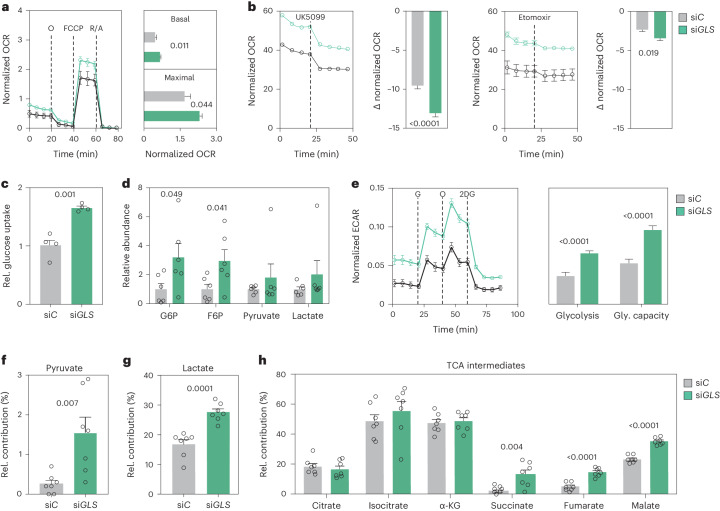
Adipocyte GLS inhibition promotes glucose utilization and oxidative metabolism. **a**, OCRs during mitostress tests in si*C* (black) or si*GLS* (green) human adipocytes (left). Data (12 replicates per condition, experiment repeated at least three times) were compared for basal respiration and maximal respiratory capacity using Student’s *t*-test (right). **b**, Metabolic fuel flex tests in si*C* or si*GLS* human adipocytes. Data (12 replicates per condition from more than three experiments) displaying the drop in OCR from baseline after drug injection (UK5099 in left or etomoxir in right) were calculated and compared using Student’s *t*-test. **c**, Glucose uptake in human adipocytes transfected with si*C* or si*GLS* (four replicates per condition, repeated twice). Data were compared using Student’s *t*-test. **d**, Intracellular levels of glucose-6-phosphate (G6P), fructose-6-phosphate (F6P), pyruvate and lactate in human adipocytes transfected with si*C* or si*GLS* (six replicates per condition). Data were compared using Student’s *t*-test. **e**, ECAR during glycostress tests in si*C* or si*GLS* human adipocytes (left). Data (12 replicates per condition, repeated more than three times) were compared for glycolysis and glycolytic (gly.) capacity (right) using Student’s *t*-test. **f**–**h**, Glucose-^13^C incorporation in pyruvate (**f**), secreted lactate (**g**) and TCA intermediates (**h**) in human adipocytes transfected with si*C* or si*GLS* (seven replicates per condition). Data were compared using Student’s *t*-test. Data in all panels show mean ± s.e.m. Relevant *P* values are shown. 2DG, 2-deoxy-d-glucose; α-KG, alpha-ketoglutarate; FCCP, carbonyl cyanide-4-(trifluoromethoxy)phenylhydrazone; G, glucose; norm., normalized; O, oligomycin; R/A, rotenone/antimycin A; rel., relative. Source data

**Extended Data Fig. 4:**
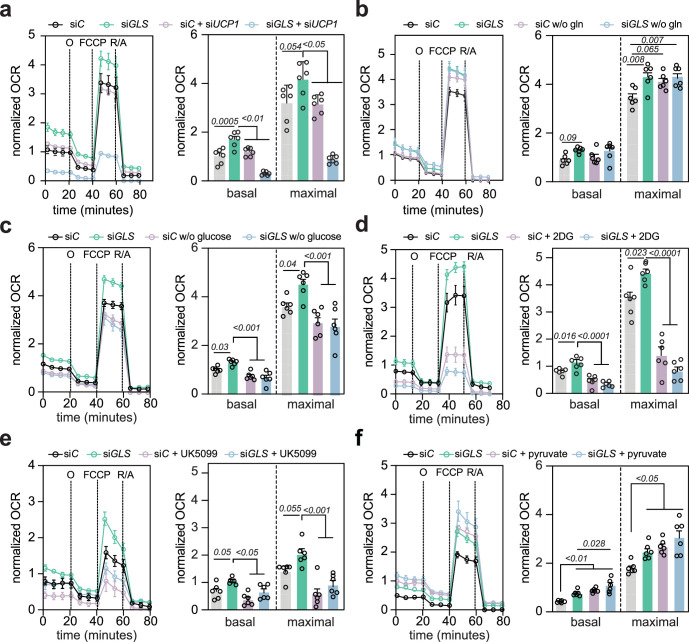
Glycolytic activity drives oxygen consumption rates following GLS depletion. **a**. Oxygen consumption rates (OCR) during mitostress tests in si*C* or si*GLS* human adipocytes co-transfected with si*C* or si*UCP1* (left). Data (six replicates per condition, repeated two times) were compared for basal and maximal respiratory capacity. Results were compared using two-way ANOVA and Tukey’s post-hoc test and *p* values are shown (right). **b**. OCR during mitostress tests in *siC* or *siGLS* human adipocytes incubated with or without glutamine for 48 hours (left). Data were compared for basal and maximal respiratory capacity. Results were compared using two-way ANOVA and Tukey’s post-hoc test and the *p* value is shown (right) (six replicates per condition, repeated three times). **c**. OCR during mitostress tests in si*C* or si*GLS* human adipocytes (left) incubated with or without glucose. Data were compared for basal and maximal respiratory capacity. Results were compared using two-way ANOVA and Tukey’s post-hoc test and *p* values are shown (right) (six replicates per condition, repeated two times). **d**. OCR during mitostress tests in si*C* or si*GLS* human adipocytes (left) incubated with or without 2-deoxy-D-glucose (2DG). Data were compared for basal and maximal respiratory capacity. Results were compared using two-way ANOVA and Tukey’s post-hoc test and *p* values are shown (right) (six replicates per condition, repeated two times). **e**. OCR during mitostress tests in si*C* or si*GLS* human adipocytes (left) incubated with or without UK5099. Data (repeated two times) were compared for basal and maximal respiratory capacity. Results were compared using two-way ANOVA and Tukey’s post-hoc test and *p* values are shown (right) (six replicates per condition, repeated two times). **f**. OCR during mitostress tests in si*C* or si*GLS* human adipocytes (left) incubated with or without sodium pyruvate. Data (repeated two times) were compared for basal and maximal respiratory capacity. Results were compared using two-way ANOVA and Tukey’s post-hoc test and *p* values are shown (right) (six replicates per condition, repeated two times). Data in all panels show mean ± S.E.M. **Abbreviations:** FCCP = carbonylcyanide-p-trifluoromethoxyphenylhydrazone, O = oligomycin, R/A = rotenone/antimycin A. Source data

To assess the functional implications of the observed transcriptional reprogramming, we measured basal and maximal oxygen consumption rates (OCR). Consistent with our findings at the mRNA and protein levels, si*GLS* versus si*C* adipocytes displayed elevated basal and maximal OCR demonstrating that mitochondrial respiration was increased (Fig. 3a). These effects required UCP1 and were phenocopied by glutamine depletion (Extended Data Fig. 4a,b). To determine substrate preference, we performed mitochondrial fuel flex tests in the basal energetic state. For this, we treated cells with etomoxir (a carnitine palmitoyl transferase 1 inhibitor) or UK5099 (a mitochondrial pyruvate carrier inhibitor). Our data revealed that while both fatty acids and glucose were metabolized at higher rates in si*GLS* adipocytes compared to control cells, glucose was the major energy source (Fig. 3b). In line with this observation, we found that si*GLS* versus si*C* cells displayed increased uptake of glucose (Fig. 3c), elevated intracellular levels of several glucose-derived metabolites (Fig. 3d) and increased glycolytic activity as measured by the extracellular acidification rate (ECAR) (Fig. 3e). By incubating the cells with [U-^13^C6] glucose, we confirmed these results as the label was incorporated to a higher degree into pyruvate, secreted lactate and TCA intermediates in *GLS*-depleted compared to control cells (Fig. 3f–h). To test whether the increased OCR in si*GLS* adipocytes was dependent on glucose metabolism, we depleted the cells of extracellular glucose, inhibited glycolysis with 2-deoxy-glucose or suppressed mitochondrial pyruvate oxidation using UK5099. Under all these conditions, the phenotype of *GLS*-depleted cells was reversed (Extended Data Fig. 4c–e). Conversely, incubations with pyruvate amplified OCR in both si*C* and si*GLS* cells (Extended Data Fig. 4f). These findings suggest that mitochondrial activation on *GLS* depletion is characterized by increased glucose catabolism.

**Extended Data Fig. 5:**
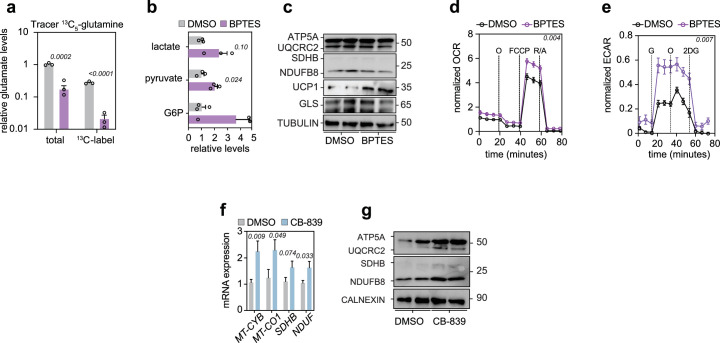
Pharmacologic inhibition of GLS induces oxidative activity. **a**. Effects of BPTES on glutamate labeling using ^13^C-labeled glutamine (three replicates per condition). **b-e**. Effect of BPTES on (**b**) glucose-6-phosphate (G6P), pyruvate and lactate levels, (**c**) electron transport chain, UCP1 and GLS protein levels (three replicates per condition), (**d**) oxygen consumption rates (OCR) (six replicates per condition, repeated >three times) and (**e**) extracellular acidification rates (ECAR) in human adipocytes (six replicates per condition, repeated three times). In panel **c**, the proteins from the same samples were loaded on two different gels in parallel. **f**. Effects of CB-839 on primary human mature adipocytes (n = 12 from two independent experiments) on gene expression of mitochondrial markers. **g**. Effects of CB-839 in human mature adipocytes on protein expression of electron transport chain proteins (repeated two times). Data in **a, b, f** were compared using Student’s *t*-test and *p* values are shown. Data in **d, e** were compared using two-way ANOVA and *p* values reflecting treatment effects are shown. Data in panels **a-b** and **d-f** show mean ± S.E.M. **Abbreviations:** 2DG = 2-Deoxy-D-glucose, DMSO = dimethyl sulfoxide, FCCP = carbonylcyanide-p-trifluoromethoxyphenylhydrazone, O = oligomycin, R/A = rontenone/antimycin A. Source data

In addition to genetically reducing *GLS* levels, we performed short-term experiments following GLS inhibition with bis-2-(5-phenylacetamido-1,3,4-thiadiazol-2-yl)ethyl sulfide (BPTES). Mass spectrometry-based analyses confirmed that BPTES-treated adipocytes displayed reduced glutamine conversion (determined as ^13^C-glutamine incorporation) and increased intracellular levels of multiple glycolytic metabolites, ETC protein levels, OCR and ECAR (Extended Data Fig. 5a–e) compared to control cells. Similar effects on mitochondrial gene expression and protein abundance were observed with the GLS inhibitor teleglenastat (CB-839) (Extended Data Fig. 5f,g). Thus, both genetic and pharmacologic reductions in glutaminolysis promote glycolytic and oxidative activity in adipocytes.

### HIF1α promotes glycolysis following *GLS* depletion

**Fig. 4:**
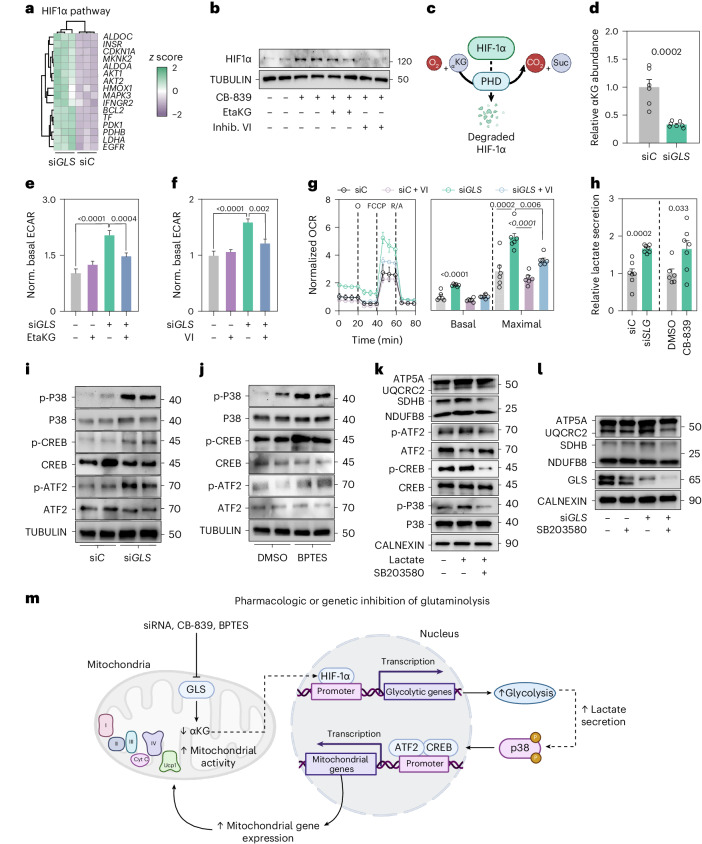
HIF1α promotes glycolysis following adipocyte *GLS* depletion. **a**, Heatmap of HIF1α target genes altered in si*GLS* versus si*C* treated human adipocytes (three replicates per condition). **b**, Effects of CB-839 in human adipocytes on protein levels of HIF1α. Effects of eta-ketoglutarate (EtaKG) or the HIF1α inhibitor VI (Inhib. VI) are shown (repeated twice). **c**, Illustration of how alpha-ketoglutarate (α-KG) degrades HIF1α through prolyl hydroxylase domain (PHD) proteins. **d**, Relative levels of α-KG in si*GLS* (*n* = 7) versus si*C* (*n* = 6) human adipocytes (repeated twice). Data were compared using Student’s *t*-test. **e**, Normalized (norm.) basal ECAR during glycostress tests in si*C* or si*GLS* human adipocytes treated with EtaKG (11 replicates per condition, repeated twice). Data were compared using two-way analysis of variance (ANOVA) and Tukey’s post hoc test. **f**, Normalized basal ECAR rate during glycostress tests in si*C* or si*GLS* human adipocytes treated with the HIF1α inhibitor VI (12 replicates per condition, repeated twice). Data were compared using two-way ANOVA and Tukey’s post hoc test. **g**, Normalized OCR in si*C* or si*GLS* human adipocytes treated with or without the HIF1α inhibitor VI (left) (six replicates per condition, repeated two times). Data were compared for basal respiration and maximal respiratory capacity using two-way ANOVA and Tukey’s post hoc test (right). **h**, Lactate secretion in human adipocytes transfected with si*C* (*n* = 7) or si*GLS* (*n* = 7) (left) or treated with DMSO (*n* = 6) or CB-839 (*n* = 7) (right). Data were compared using Student’s *t-*test (repeated more than three times). **i**,**j**, Representative western blots of total and phosphorylated protein levels of p38 MAPK, CREB and ATF2 in human adipocytes transfected with si*C* or si*GLS* (**i**) or incubated with DMSO or BPTES (**j**, repeated twice). Proteins from the same experiment were loaded on four different gels in parallel for **i**,**j**. **k**, Representative western blots of total and phosphorylated protein levels of p38 MAPK, ATF2 as well as ETC proteins in human adipocytes incubated with or without lactate (for 30 min) and the p38 MAPK inhibitor SB203580 (pretreatment for 2.5 h) (repeated twice), Proteins from the same experiment were loaded on five different gels in parallel. **l**, Representative western blot showing the ETC and GLS protein levels in si*C* or si*GLS* human adipocytes treated with or without the p38 MAPK inhibitor SB203580 (experiment repeated twice). **m**, Model of how inhibition of glutaminolysis drives a metabolic reprograming promoting adipocyte thermogenesis. Data **d**–**h** show mean ± s.e.m. Relevant *P* values are shown. **c**,**m**, Created with BioRender.com. Source data

**Extended Data Fig. 6:**
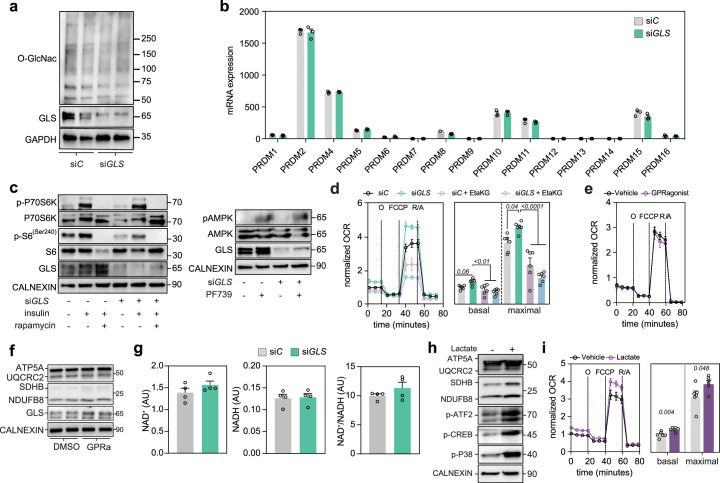
Links between GLS depletion and oxygen consumption. **a**. Western blot of global protein O-GlcNAcylation in si*C* or si*GLS* human adipocytes. Representative results are displayed (repeated two times). **b**. Expression of indicated *PRDM* genes in si*C* or si*GLS* human adipocytes based on RNA-seq data from human adipocytes transfected with si*C* or si*GLS* (three replicates per condition). **c**. The mTORC1 activity (left panel) in si*C* or si*GLS* human adipocytes was assessed by western blot analysis. This was performed by treating cells with or without an mTORC1 activator (insulin, 15 minutes treatment), and by additionally treating with or without an mTORC1 inhibitor (rapamycin) for three hours in insulin-deprived cells (repeated three times). The AMPK activity (right panel) in human adipocytes subjected to si*C* or si*GLS* was assessed through western blot analysis. Cells were incubated cells with or without an AMPK activator (PF739) for 24 hours (repeated two times). Proteins from the same experiments were loaded on two different gels in parallel for the panels on the left and on two different gels for the panels on the right. **d**. Oxygen consumption rates during mitostress tests in si*C* or si*GLS* human adipocytes (left) incubated with or without eta-ketoglutarate (EtaKG). Data were compared for basal respiration and maximal respiratory capacity (six replicates per condition, repeated two times). Data were compared using two-way ANOVA and Tukey’s post-hoc test, *p* values are shown (right). **e**. Oxygen consumption rates during mitostress test in human adipocytes incubated with a GPR81 agonist (six replicates per condition, repeated two times). **f**. Representative examples of ETC protein levels in human adipocytes incubated with GPR81 agonist (two replicates per condition, repeated two times). **g**. NAD^+^, NADH and the ratio in si*C* or si*GLS* human adipocytes (four replicates per condition, repeated two times). **h**. Representative examples of ETC protein and P38 MAPK pathway levels in human adipocytes incubated with lactate for 30 minutes (repeated three times). Proteins from the same samples were loaded on two different gels in parallel. **i**. Oxygen consumption rates during mitostress test in human adipocytes incubated with sodium lactate (six replicates per condition, repeated two times). Data were compared using Student’s *t*-test and *p* values are shown (right). Data in panels **d-e**, **g, i** show mean ± S.E.M. Source data

We hypothesized that altered glutaminolysis in adipocytes is linked to the transcriptional rewiring of metabolism through intracellular protein O-GlcNAcylation^7^ or by altered expression of the histone lysine methyltransferase PR/SET domain 9 (encoded by *PRDM9*)^19^. However, we found no discernible changes in O-GlcNAcylation comparing si*GLS* versus si*C* cells (Extended Data Fig. 6a) and *PRDM9* was undetectable in our cell model (Extended Data Fig. 6b). Next we determined whether hypoxia-inducible factor 1α (HIF1α), adenosine monophosphate-activated protein kinase (AMPK) or mammalian target of rapamycin complex 1 (mTORC1) promoted the observed phenotype as these proteins are established regulators of energy metabolism^20–22^. While the activities of AMPK and mTORC1 appeared unaffected (Extended Data Fig. 6c), we observed an increase in the expression of HIF1α target genes in *GLS*-depleted versus control cells (Fig. 4a). To follow up on this, we confirmed that HIF1α protein levels were upregulated following GLS inhibition (Fig. 4b). We reasoned that this could be secondary to reductions in α-ketoglutarate, as this metabolite is a substrate for proline hydroxylation and subsequent degradation of HIF1α (Fig. 4c)^23^. Accordingly, we observed a reduction in alpha-ketoglutarate levels in *GLS*-depleted cells (Fig. 4d), and supplementation with eta-ketoglutarate attenuated the increase in HIF1α in cells with reduced GLS activity (Fig. 4b). HIF1α has been shown to promote glycolytic activity^24^. In line with this, we observed that eta-ketoglutarate supplementation or HIF1α inhibition (using inhibitor VI) abrogated the increase in ECAR following *GLS* depletion (Fig. 4e,f). Both these treatments also reversed the elevated OCR observed in si*GLS* cells (Fig. 4g and Extended Data Fig. 6d). Taken together, our results indicate that reductions in GLS activity affect HIF1α stability, via lowered α-ketoglutarate levels, and that this step is required for the observed increases in glycolysis and mitochondrial activity.

### *GLS* depletion promotes mitochondrial activity via p38 MAPK

Next, we investigated how reduced GLS activity in adipocytes induces mitochondrial capacity downstream of glycolytic activation. Consistent with elevated ECAR, both *GLS* silencing and CB-839 treatment resulted in increased lactate levels (Fig. 4h), which in turn has been linked to adipocyte browning via (1) signalling through the hydroxycarboxylic acid receptor 1 (also known as G protein-coupled receptor 81, GPR81), (2) altering the redox state (NAD^+^/NADH ratio) and/or (3) activating the mitogen-activated protein kinase (MAPK) p38 (refs. ^25–28^). To dissect between these potential regulatory steps, we first incubated adipocytes with a GPR81 agonist but found no changes in OCR and ETC protein levels (Extended Data Fig. 6e,f). Furthermore, we found that NAD^+^/NADH levels were unaltered in si*GLS* versus si*C* adipocytes (Extended Data Fig. 6g). However, we observed that the phosphorylation of p38 MAPK, as well as its downstream targets cyclic AMP-responsive element binding protein (CREB) and activating transcription factor 2 (ATF2) were increased following *GLS* depletion and GLS inhibition (Fig. 4i,j). Similar effects on p38/CREB/ATF2 phosporylation and increases in ETC proteins and OCR were observed following lactate incubations (Extended Data Fig. 6h,i). The dependency on p38 MAPK was tested by pretreatment with an inhibitor of this kinase (SB203580). In this experimental setup, the effects of lactate and si*GLS* on ETC proteins were abrogated (Fig. 4k,l). Altogether, our data suggest that glutaminolysis coordinates glycolysis and mitochondrial capacity via HIF1α, lactate and p38 MAPK (Fig. 4m).

### GLS inhibition results in weight loss and WAT browning

**Fig. 5:**
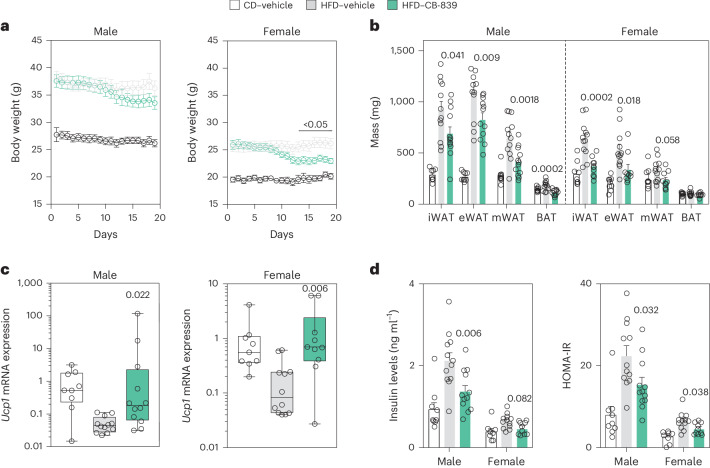
Pharmacological *Gls* inhibition in male and female mice reduces fat mass and induces adipocyte *Ucp1* expression in inguinal WAT. **a**, Body weight changes in male and female mice fed chow (CD) (*n* = 9 for each gender) or a HFD, treated either with vehicle (*n* = 12 for each gender) or with 200 mg kg^−1^ of CB-839 (*n* = 12 males and *n* = 10 females) administered daily by gavage for 19 days. Data were analysed by two-way ANOVA and Tukey’s post hoc test. **b**, The weights of one pad out of the two pads of inguinal (iWAT), one pad out of the two pads of epigonadal (eWAT) and mesenteric (mWAT) WAT as well as BAT were measured in the male and female mice described in **a**. Data were analysed by one-way ANOVA and Tukey’s post hoc test. **c**, *Ucp1* gene expression profiles were analysed in iWAT samples obtained from the male and female mice described above. Data are presented as min–max (median) and were compared using non-parametric (Kruskal–Wallis) one-way ANOVA. **d**, The circulating levels of insulin were measured and the Homeostatic Model Assessment-Insulin Resistance (HOMA-IR) index was calculated in male and female mice fed CD (*n* = 9 males and *n* = 8 females) or HFD, treated either with vehicle (*n* = 12 for each gender) or with CB-839 (*n* = 12 males and *n* = 10 females) as described above. Data were analysed by one-way ANOVA and Dunnett’s post hoc test. Data in **b** and **d** show mean ± s.e.m. The *P* values are displayed only for the comparison between HFD treated with either vehicle or CB-839. Source data

**Extended Data Fig. 7:**
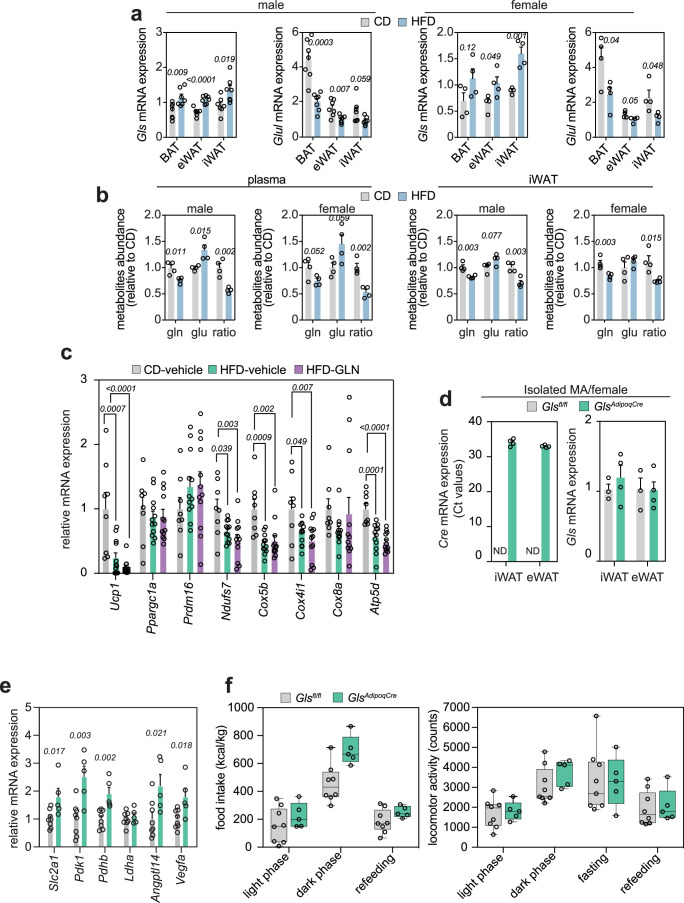
Cross-talk between high fat diet and glutamine metabolism in male and female mice. **a**. *Gls* and *Glul* mRNA expression in different adipose depots in male or female mice fed chow (CD) or high fat diet (HFD) for 15 weeks (seven male mice and four female mice per condition). Data were compared using Student’s *t*-test and *p* values are shown. **b**. Glutamine (gln) and glutamate (glu) levels including glutamine-to-glutamate ratio were measured in the plasma (left) or iWAT (right) of male and female mice fed chow (CD) or high fat diet (HFD) for 15 weeks (four mice per gender and per condition). Data were compared using Student’s *t*-test and *p* values are shown. **c**. Expression of genes encoding UCP1 and electron transport chain proteins in iWAT of mice fed chow (CD-vehicle, n = 8) or HFD injected intraperitoneally with either vehicle (HF-vehicle, n = 12) or glutamine (HF-GLN, n = 12) for two weeks. This cohort of mice has been described before^7^. Data were compared using one-way ANOVA and Tukey’s post-hoc test, *p* values are shown. **d**. Ct values of *Cre* (left) and *Gls mRNA* expression (right) in mature adipocytes from eWAT or iWAT of *Gls*^*fl/fl*^ (n = 3) and *Gls*^*AdipoqCre*^ (n = 4) female mice. **e**. Expression of HIF1α target genes in iWAT of *Gls*^*fl/fl*^ (n = 8) and *Gls*^*AdipoqCre*^ (n = 5) male mice. Data were analyzed by Student’s *t*-test and *p* values are shown. **f**. Food intake (left) and locomotor activity (right) in *Gls*^*fl/fl*^ (n = 8) and *Gls*^*AdipoqCre*^ (n = 5) male mice. Data (eight and five mice per condition) were analyzed by two-way ANOVA with no statistical significance detected. Data presented in box plot represent min., max and median. Data in panels **a-e** show mean ± S.E.M. **Abbreviations:** Ct = threshold cycles, *Cre* = Cre recombinase-mediated DNA recombination, eWAT = epididymal white adipose tissue, iWAT = inguinal white adipose tissue, MA = mature adipocytes, ND = non-detectable. Source data

We next assessed whether glutamine metabolism is dysregulated by obesity also in mice. For this, we obtained adipose tissue samples from male and female mice fed chow or high-fat diet (HFD). In two WAT depots, we found that *Gls* mRNA levels were increased while *Glul* levels were decreased following 15 weeks of HFD (Extended Data Fig. 7a). Concomitantly, we observed a reduction in glutamine-to-glutamate ratios in plasma and WAT (Extended Data Fig. 7b). As these results recapitulated and extended our findings in human WAT, we tested whether GLS inhibition in mice influenced WAT mass and carbohydrate metabolism via WAT browning. For this, mice were fed HFD for 7 weeks where the last 19 days included daily gavage administration of either CB-839 or vehicle. As shown in Fig. 5a,b, CB-839 treatment resulted in total body and fat mass loss in both male and female mice. qPCR analyses revealed an increase in the expression of *Ucp1* in inguinal WAT (iWAT) (Fig. 5c). In addition, this short-term treatment resulted in reduced circulating insulin levels and decreased HOMA-IR (Fig. 5d). As the observed effects could be due to increased systemic glutamine availability, we treated HFD-fed mice with intraperitoneal glutamine injections for 2 weeks and measured the expression of genes encoding factors involved in browing and bioenergetic pathways in iWAT. In this setup, glutamine did not affect the expression of any of the measured genes (Extended Data Fig. 7c), suggesting that the effects observed on GLS inhibition are not secondary to systemic glutamine build-up.

### Adipocyte *Gls* deletion alters WAT mass and fat cell morphology

**Fig. 6:**
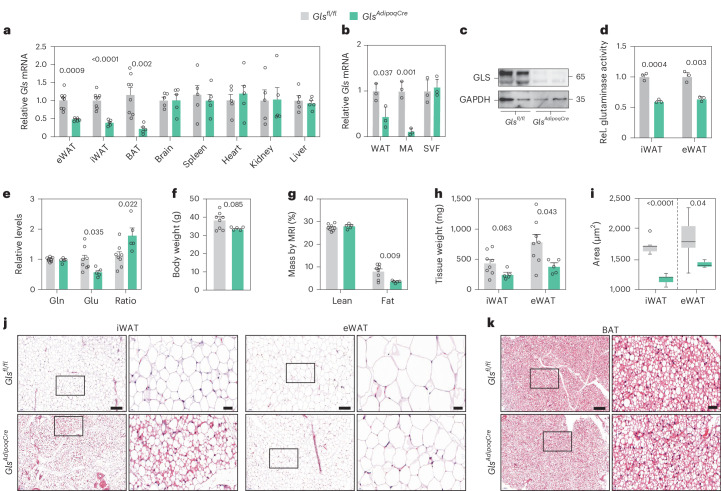
Adipocyte *Gls* depletion promotes a browning phenotype in inguinal WAT. **a**, *Gls* mRNA expression in adipose depots and different organs from *Gls*^*fl/fl*^ (*n* = 8 for adipose tissues (iWAT, eWAT and BAT) and *n* = 5 for other organs) and *Gls*^*AdipoqCre*^ (*n* = 5) mice. **b**, *Gls* mRNA expression in intact WAT, mature adipocytes (MA) and the stromal vascular fraction (SVF) in the two genotypes (*n* = 3 per condition). **c**, Gls protein expression in mature adipocytes isolated from iWAT of *Gls*^*fl/fl*^ and *Gls*^*AdipoqCre*^ mice (two mice per condition). **d**, GLS activity in iWAT and eWAT of *Gls*^fl/fl^ and *Gls*^AdipoqCre^ mice (*n* = 3 per condition). **e**, Data from mice in **a** displaying the levels of glutamine (gln), glutamate (glu) and the glutamine-to-glutamate ratio in iWAT of *Gls*^*fl/fl*^ (*n* = 8) and *Gls*^*AdipoqCre*^ (*n* = 5) mice. **f**, Body weight of *Gls*^*fl/fl*^ (*n* = 8) and *Gls*^*AdipoqCre*^ (*n* = 5) mice. **g**, Fat and lean body mass determined by magnetic resonance imaging (MRI) in *Gls*^*fl/fl*^ (*n* = 8) and *Gls*^*AdipoqCre*^ (*n* = 5) mice. **h**, Weight of iWAT and eWAT of *Gls*^*fl/fl*^ (*n* = 8) and *Gls*^*AdipoqCre*^ (*n* = 5) mice. **i**, Fat cell size of iWAT and eWAT (five mice per condition) measured as described in the Methods. Boxplots display the median and the 1.5× IQR method is used to determine the length of the whiskers. **j**, Representative haematoxylin and eosin staining of iWAT and eWAT with a specified area magnified (to the right of each image). Scale bars represent 100 μm for the images and 20 μm in the magnified areas. **k**, Representative haematoxylin and eosin staining of BAT with a determined area magnified (to the right side of the images). Scale bars same as in **j**. **a**,**b** and **d**–**h** show mean ± s.e.m. Data in **a**, **b** and **d**–**i** were compared using Student’s *t*-test. Relevant *P* values are shown. eWAT, epigonadal WAT; iWAT, inguinal WAT; Rel., relative. Source data

To test the effect of adipocyte GLS depletion in vivo, we generated adipocyte-specific *Gls* knockout mice (*Gls*^*AdipoqCre*^) by crossing *Gls*-floxed and *adiponectin*-Cre transgenic mice. In male mice, we confirmed that *Gls* expression was selectively reduced in WAT and BAT but not in the other investigated organs (Fig. 6a). Within WAT, the depletion of *Gls* was evident in the mature white adipocyte fraction, but not in the stromal cells (Fig. 6b), and resulted in reduced GLS protein levels and GLS activity as well as increased plasma glutamine-to-glutamate ratio compared with Cre-negative littermates (*Gls*^*fl/fl*^) (Fig. 6c–e). In contrast to these data, we were not able to deplete *Gls* in mature adipocytes from female mice despite clear mRNA expression of Cre recombinase (Extended Data Fig. 7d). Further studies were therefore performed only in male mice. In these animals, we found that the two genotypes exhibited similar total and lean body weights (Fig. 6f,g). The knockout mice, however, displayed a reduction in fat mass, where both the inguinal and epididymal adipose depots were reduced and contained smaller fat cells (Fig. 6h,i). Our histological assessments also revealed a larger proportion of inguinal adipocytes with multilocular lipid droplets in *Gls* knockout mice (Fig. 6j), while there were no discernible differences in the morphology of the BAT comparing genotypes (Fig. 6k).

### *Gls* deletion promotes energy expenditure and metabolic health

**Fig. 7:**
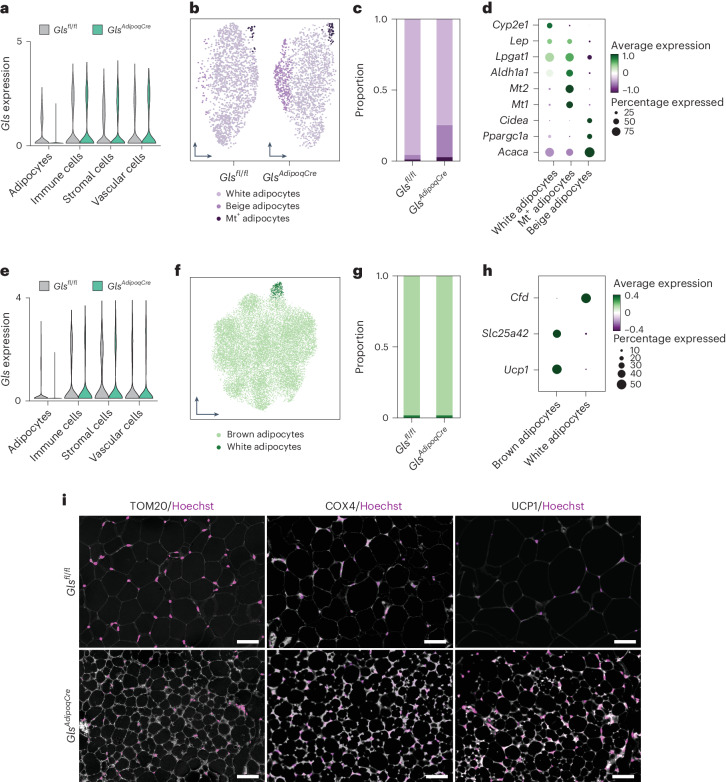
Subclustering of mouse adipocytes identifies a distinct beige subpopulation within the iWAT of *Gls*^*AdipoqCre*^ mice. **a**, *Gls* gene expression for each cell population in the single-nucleus RNA sequencing dataset from inguinal WAT (iWAT) of *Gls*^*fl/fl*^ and *Gls*^*AdipoqCre*^ mice (*n* = 5 for each genotype). **b**, UMAP projection of adipocytes in iWAT of *Gls*^*fl/fl*^ and *Gls*^*AdipoqCre*^ mice. **c**, Proportion of adipocyte subpopulations in iWAT of *Gls*^*fl/fl*^ and *Gls*^*AdipoqCre*^ mice. **d**, Top marker genes across the three identifiable subpopulations in iWAT presented in a dot plot where circle sizes and colours are proportional to the detection rate and expression of the marker genes, respectively. **e**, *Gls* gene expression for each cell population in the single-nucleus RNA sequencing dataset from BAT of *Gls*^*fl/fl*^ and *Gls*^*AdipoqCre*^ mice. **f**, UMAP projection of adipocytes in BAT. **g**, Proportion of adipocyte subpopulations in BAT of *Gls*^*fl/fl*^ and *Gls*^*AdipoqCre*^ mice. **h**, *Ucp1*, *Slc25a42* and *Cfd* mRNA expression of brown adipocyte and inguinal white adipocyte of *Gls*^*fl/fl*^ and *Gls*^*AdipoqCre*^ mice. **i**, Representative microphotographs of iWAT displaying immunofluorescence signal of TOM20, COX4 and UCP1 protein in *Gls*^*fl/fl*^ and *Gls*^*AdipoqCre*^ mice. Hoechst was used to stain nuclei. Scale bars, 50 μm. COX4, cytochrome C oxidase subunit; Cfd, complement factor D; Mt, metallothionein 1A; Slc25a42, solute carrier family 25 member 42; TOM20, translocase of outer mitochondrial membrane 20; UMAP, uniform manifold approximation and projection. Source data

As a hallmark feature of beige adipocytes is lipid droplet multilocularity^29^, we next investigated the cellular composition of iWAT using single-nucleus RNA sequencing. We found four major cell classes, that is, adipocytes, leukocytes, vascular cells and fibroblasts and adipogenic progenitors, where *Gls* was selectively depleted in the adipocyte fraction of *Gls*^*AdipoqCre*^ mice (Fig. 7a). Through targeted examinations of the fat cells, three distinct subtypes were identified: white, beige and a minority of metallothionein-expressing adipocytes. Notably, the population of the beige subtype was substantially augmented in *Gls*^*AdipoqCre*^ mice (Fig. 7b–d). Similar analyses in BAT revealed no obvious genotype-related differences in the cellular composition between genotypes (Fig. 7e–h). To extend these results, we performed iWAT immunostainings with antibodies directed against multiple mitochondrial proteins. Our data showed that immunofluorescence signals for UCP1, COX4 and TOM20 were higher in iWAT from *Gls*^*AdipoqCre*^ versus control mice (Fig. 7i). Thus, our findings suggest that adipocyte-specific depletion of *Gls* results in iWAT browning with concomitant reductions in body fat.

**Fig. 8:**
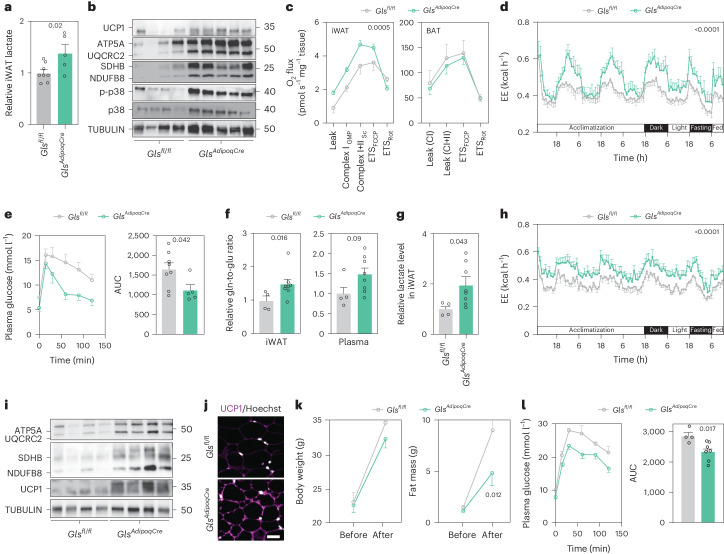
Loss of *GLS* in adipose tissue promotes mitochondrial activity and protects mice against obesity. **a**, Lactate levels in inguinal WAT (iWAT) from *Gls*^*fl/fl*^ (*n* = 8) and *Gls*^*AdipoqCre*^ (*n* = 5) mice on a chow diet. **b**, UCP1, ETC, total and phosphorylated p38 MAPK protein levels in iWAT from *Gls*^*fl/fl*^ and *Gls*^*AdipoqCre*^ mice fed chow diet. Proteins from the same samples were loaded on three different gels. **c**, High-resolution respirometry showing O_2_ consumption in iWAT (left) and BAT (right) from *Gls*^*fl/fl*^ and *Gls*^*AdipoqCr*e^ mice fed chow diet. Data (*n* = 5 for iWAT and *n* = 3 for BAT in *Gls*^*fl/fl*^, and *n* = 5 for iWAT and *n* = 4 for BAT in *Gls*^*AdipoqCr*e^) were compared using two-way ANOVA. **d**, Energy expenditure (EE) in *Gls*^*fl/fl*^ (*n* = 8) and *Gls*^*AdipoqCr*e^ (*n* = 5) mice fed chow diet measured in metabolic cages. Data were analysed by two-way ANOVA (mixed-effects analysis). **e**, Data from intraperitoneal glucose tolerance test in *Gls*^*fl/fl*^ (*n* = 8) and *Gls*^*AdipoqCr*e^ (*n* = 5) mice fed chow diet. The area under the curve (AUC) is displayed in the right-hand panel. **f**, Glutamine-to-glutamate ratio in iWAT (left) and plasma (right) from *Gls*^*fl/fl*^ (*n* = 4) and *Gls*^*AdipoqCr*e^ (*n* = 8) mice following 8 weeks of HFD. Data were compared using Mann–Whitney test for the left panel and Student’s *t*-test for the right panel. **g**, Lactate levels in iWAT from *Gls*^*fl/fl*^ (*n* = 4) and *Gls*^*AdipoqCr*e^ (*n* = 8) mice fed HFD. **h**, Energy expenditure in *Gls*^*fl/fl*^ (*n* = 4) and *Gls*^*AdipoqCr*e^ (*n* = 8) mice fed HFD. Data were analysed by two-way ANOVA (mixed-effects analysis). **i**, UCP1 and ETC protein levels in iWAT from *Gls*^*fl/fl*^ and *Gls*^*AdipoqCre*^ mice fed HFD (four mice per condition). **j**, Representative microphotographs of UCP1 immunofluorescence in iWAT from *Gls*^*fl/fl*^ and *Gls*^*AdipoqCre*^ mice fed HFD. Hoechst was used to stain nuclei. Scale bar, 50 μm. **k**, Body weight and fat mass determined by magnetic resonance imaging in *Gls*^*fl/fl*^ (*n* = 4) and *Gls*^*AdipoqCre*^ (*n* = 8) mice before and after HFD. Data were analysed by two-way ANOVA and Bonferroni’s post hoc test. **l**, Data from intraperitoneal glucose tolerance test in *Gls*^*fl/fl*^ (*n* = 4) and *Gls*^*AdipoqCr*e^ (*n* = 7) mice after 7 weeks of HFD. The AUC is displayed in the right panel. Data in **a**, **c**–**h** and **k**–**l** show mean ± s.e.m. Data in **a**, **e**, **g** and **l** were compared using Student’s *t*-test. Relevant *P* values are shown. Source data

To assess whether the observed tissue browning resulted in lactate build-up and p38 MAPK activation, we measured these parameters in iWAT. In analogy with our data on human fat cells, we found that samples from *Gls* KO mice displayed higher lactate levels and p38 MAPK phosphorylation compared to control littermates (Fig. 8a,b). Moreover, several HIF1α target genes were upregulated in iWAT of *Gls* KO mice (Extended Data Fig. 7e). We next determined whether this translates into altered metabolic phenotypes by performing high-resolution respirometry at the tissue level, whole-body indirect calorimetry and glucose tolerance tests. Our analyses revealed that iWAT from *Gls*^*AdipoqCre*^ mice displayed higher oxygen consumption than the corresponding tissues from control littermates (Fig. 8c). This contrasted with our measures in BAT where there were no genotype-related differences (Fig. 8c). By placing the mice in metabolic cages, we confirmed higher energy expenditure in *Gls*^*AdipoqCre*^ versus control mice without any changes in food intake or locomotor activity (Fig. 8d and Extended Data Fig. 7f). These differences were associated with improved glucose tolerance in *Gls* KO mice (Fig. 8e). Following HFD feeding, the increases in glutamine-to-glutamate ratio and lactate levels, elevated energy expenditure as well as iWAT browning were retained in *Gls*^*AdipoqCre*^ mice (Fig. 8f–j), which in turn protected them from HFD-induced fat mass accumulation and glucose intolerance (Fig. 8k,l). Altogether, our studies in mice corroborate our human data and indicate that glutamine-to-glutamate conversion via GLS in white adipocytes regulates energy expenditure and metabolic health.

## Discussion

Herein, we demonstrate that obesity-induced insulin resistance is characterized by increased fat cell glutaminolysis, which is evident at the transcript, protein and metabolite levels in both humans and mice. In human white adipocytes, *GLS* transcription is induced by TNF and a targeted reduction in GLS activity results in a robust induction of metabolic and thermogenic transcriptional programs with increases in glycolysis, lactate levels and mitochondrial respiration. We find that this WAT browning phenotype is dependent on p38 MAPK signalling, and is phenocopied in inguinal, but not epigonadal or brown adipose regions of male mice with adipocyte-specific GLS depletion. Consequently, these mice display increased energy expenditure and are protected against HFD-induced fat mass accumulation and glucose intolerance. Altogether, our results uncover a role for adipocyte glutamine turnover in regulating white adipocyte energy consumption and whole-body metabolic health.

A previous study has proposed that glutamine is a substantial mitochondrial energy source in white adipocytes from rats^30^. Our data indicate that this also pertains to human and mouse fat cells where inhibition of glutaminolysis results in increased use of glucose. This metabolic adaptation promotes oxidative capacity and a thermogenic phenotype via activation of the p38 MAPK pathway in both human adipocytes and mouse iWAT. Our results are in line with previous work in murine beige adipocytes demonstrating that p38 MAPK activation promotes *Ucp1* expression and oxidative capacity^31–35^. Although further studies are required to elucidate the mechanism by which glutaminolysis influences p38 MAPK signalling, we propose that GLS inhibition results in a reduction of α-ketoglutarate and thereby elevated HIF1α levels. The ensuing HIF1α-mediated increase in glycolysis exceeds the oxidative capacity of the cell, resulting in lactate build-up and activation of thermogenic transcriptional programs via p38 MAPK signalling. While this model is based on metabolite add-backs and withdrawals (glucose, pyruvate, lactate, glutamine and eta-ketoglutarate), pharmacological inhibitors (BPTES, CB-839, SB203580, Inhibitor VI and UK5099), genetic depletion (si*GLS and Gls*^*AdipoqCre*^) and gain-of-function experiments (*GLS* over expression), we acknowledge that each step may be interconnected and that the pathway may therefore be more complex than described herein. For example, while we did not observe any changes in NAD^+^/NADH in *GLS*-depleted cells, these assessments were performed in whole cell lysates after several days of *GLS* knockdown. It is possible that dynamic changes in the redox state (potentially in different organelles) may be important in promoting the browning effect as has been reported previously^25,36^. Furthermore, lactate has recently raised considerable interest where multiple effects have been attributed to this metabolite^37^, including actions independent of its metabolism^38^. Thus, further studies are needed to understand the interactions between glutamine metabolism, lactate turnover and thermogenic activation.

Comparisons of control and *Gls*^*AdipoqCre*^ mice using histological and single-nucleus RNA sequencing profiling of iWAT, revealed a substantial increase in the proportion of beige multilocular fat cells, marked by the expression of *Ppargc1a*, *Cidea* and *Acaca*, in the knockout mice. Functionally, these results were corroborated by high-resolution respirometry demonstrating higher oxygen consumption in iWAT of *Gls*^*AdipoqCre*^ mice compared with Cre-negative littermates. By contrast, no changes in BAT cellularity or tissue respiration were observed comparing genotypes. In mice, the inguinal depot is highly susceptible to undergo browning^29,39^, and our data align with previous reports demonstrating that induction of a brown-like phenotype selectively in iWAT improves metabolic health^40,41^. The mice included in the present study were housed at room temperature. It is possible that exposure to cold or thermoneutrality could affect the observed phenotype as these conditions have pronounced effects on glutamine turnover in BAT^42,43^.

Our analyses of human and mouse WAT in obesity revealed that *GLS* mRNA levels were increased in both sexes compared with relevant control groups. We therefore set out to generate adipocyte-specific *Gls* knockout mice. However, although Cre recombinase was expressed in mature fat cells of female *Gls*^*AdipoqCre*^ mice, we observed no reduction of *Gls* expression. Thus, while our conclusions in humans and wild-type mice are relevant for both sexes, the present knockout model data only pertains to male animals. Although sex-specific differences in the Cre-loxP system have been described^44,45^, we cannot currently explain the mechanisms underlying the poor gene editing efficiency in female mice. However, administration of CB-839 had similar effects in both sexes, indicating that there are no qualitative differences in the role of glutamine turnover between females and males.

We provide evidence that increased white adipocyte glutaminolysis is linked to a reduction in the glutamine-to-glutamate ratio in both WAT and plasma. Conversely, we find that an adipocyte-specific depletion of GLS in male mice results in increased circulating glutamine versus glutamate levels. Together with data from healthy human participants^11^ and rats^46^, this indicates that WAT contributes to plasma glutamine-to-glutamate stoichiometry and that this ratio partly reflects WAT function. Similar effects have been reported in non-alcoholic steatohepatitis where hepatic *GLS* expression is increased, the plasma glutamine-to-glutamate ratio is decreased and enzyme inhibition reduces the hepatocyte lipid content^47^. Thus, inhibition of glutaminolysis may be a potential therapeutic target to improve energy homeostasis in people living with obesity, type 2 diabetes and/or steatohepatitis. As we found here that a short-term treatment with CB-839 in HFD-fed mice resulted in weight loss and iWAT browning, randomized controlled trials testing the efficacy of GLS inhibitors such as CB-839 in improving metabolic health would be interesting to explore. In clinical studies, these compounds have been shown to be safe and they are currently being studied as anticancer drugs^48^.

In conclusion, our results highlight that glutamine metabolism in white adipocytes is an important determinant of energy consumption and metabolic health. We base this on multiple model systems where reductions in GLS activity specifically in fat cells, induces a transcriptional response that rewires glucose oxidation and increases mitochondrial respiratory capacity. The resulting increase in energy expenditure protects against HFD-induced glucose intolerance in mice and correlates with a beneficial clinical profile in humans.

## Methods

### Clinical studies

#### Anthropometric and metabolic examinations

All examinations were conducted in the morning after an overnight fast. Anthropometric measures were determined; obesity was defined as body mass index (BMI) ≥ 30 kg m^−^^2^, and waist and hip circumferences were used to calculate waist-to-hip ratio. Body fat percentage was determined by bioimpedance (Bodystat). Energy expenditure was measured by indirect calorimetry using an open-system ventilated hood (Deltatrac II; Datex-Ohmeda). Blood samples were obtained to measure circulating levels of leptin and insulin. Homeostasis Model Assessment of insulin resistance (HOMA-IR) was calculated as (fP-glucose in mmol l^−1^ × fS-insulin in mU l^−1^)/22.5 (ref. ^49^). Participants in cohort 1 (which includes people living with obesity scheduled for bariatric surgery and non-obese controls, NCT01727245) underwent a hyperinsulinaemic euglycemic clamp as described^50^. The mean glucose infusion rate (glucose disposal) between 60 and 120 min was determined (*M* value is mg of glucose uptake per kg of body weight per min) and was expressed corrected for mean plasma insulin during steady state (M/I). Clinical data for cohorts 1 and 2 are presented in Supplementary Table 1.

#### Investigations of WAT biopsies

Subcutaneous WAT needle biopsies were obtained under local anaesthesia from the periumbilical area^51^. These isolated fat cells were prepared by collagenase digestion^52^. Mean fat cell volume was determined and lipogenesis experiments were performed as described^53^. For the latter, the medium was supplemented with a low concentration of unlabelled and tritiated glucose and insulin-stimulated incorporation of ^3^H into total lipids was determined. Results were expressed as amount of glucose incorporated into lipids (nmol glucose 2 h^−1^ (10^7^ fat cells)^−1^).

#### Omics in clinical samples

Plasma and WAT metabolomics data from cohort 1 and WAT transcriptomic data from cohort 3 were generated and presented in ref. ^7^, ref. ^54^ and ref. ^15^, respectively. Targeted gene expression analyses in men and women were performed in previously published data from ref. ^14^. For correlations between metabolites, gene expression and clinical parameters, the Hmisc package (rcorr function) in R Studio (v.4.2.1) was used.

#### Ethical approvals

All studies were approved by the regional ethics boards in Stockholm (cohort 1 and 3) and Paris (cohort 2), and informed written consent was obtained from all study participants.

### Mouse studies

For all animal studies, mice were handled following the European Union laws and guidelines for animal care, health inventories were performed according to the guidelines of the Federation of European Laboratory Animal Science Associations and special care was taken to minimize animal suffering and to reduce the number of mice used.

#### HFD intervention in wild-type mice

Male C57BL/6J mice, sourced from Charles River Laboratories (France) at 7 weeks of age, were group-housed under a 12-h light-dark cycle with ad libitum access to food and water. Following 1 week of acclimatization, the mice were assigned to either a standard chow diet or a HFD for an additional 15 weeks. For the CB-839 study, C57BL/6J male mice from Envigo (France) were acclimatized to a standard chow diet for 2 weeks. Housed in groups of five, they were subjected to a 12-h light/12-h dark cycle with ad libitum access to food and water. On reaching 10 weeks of age, mice were switched to either a standard chow diet or an HFD for 5 weeks. After 3 weeks on the HFD, mice were randomly allocated to treatment groups on the basis of body weight. Those on the HFD received daily gavage administration of CB-839 (200 mg kg^−1^ body weight) or vehicle for 19 days (25% (w/v) hydroxypropyl-β-cyclodextrin (ThermoFisher) in 10 mmol l^−1^ citrate buffer (pH 2)), while the standard chow group received vehicle only. Blood glucose and plasma insulin levels were monitored 12 days posttreatment initiation. After 19 days, mice were euthanized and tissue weights were recorded. Samples were snap-frozen in liquid nitrogen. All mice were housed in the animal facility of Pitié-Salpetrière, in conformity with EU regulations, and studied according to a protocol that received ethics approval from the French ministry for research.

#### Generation, interventions and phenotyping of Gls^Adipoq-Cre^ mice

Adiponectin-Cre mice were bred with *Gls*^*fl/fl*^ mice (Gls^tm2.1Sray^/J, stock 017894) to generate adipocyte-specific *Gls*-depleted mice (*Gls*^*AdipoqCre*^). *Gls*^*fl/fl*^ littermates were used as controls. Mice were housed in groups at the KM-B animal facility in ventilated cages (with a 12-h light/12-h dark cycle (lights between 6:00 and 18:00) in a temperature-controlled (20–24 °C, 50% humidity) facility with ad libitum access to food and water. All experimental procedures were approved by the Stockholm North Animal Ethical Committee. Five to seven-week-old male mice were fed with an HFD (D12492i, 60% kcal fat, Research Diets) for a duration of 7 weeks. Five weeks after the start of the HFD, mice were fasted for 4 h and glucose tolerance was assessed by an intraperitoneal glucose injection (1.5 g kg^−1^). Blood glucose concentration was monitored from the tail tip using a glucometer (Contour XT, Bayer) before and at the indicated time intervals following glucose injection. Indirect calorimetry was performed 1 week later in the Phenomaster Home cage system (TSE Systems). Ambulatory and locomotor activity was automatically assessed by counting the number of photo beam breaks in the *x* and *y* axis. A feeding sensor monitored food intake without disturbances by the experimenters. Lean and fat mass were measured before the start of the HFD and the indirect calorimetry protocols using the EchoMRI-100 (EchoMRI). Seven weeks after the start of the HFD protocol, at 13–15 weeks of age, mice were euthanized under general anaesthesia by avertin injection, and the wet weight of each dissected tissue was measured. Subsequently, one part of the samples was snap-frozen in liquid nitrogen and one part was fixed in in 4% paraformaldehyde (PFA). The same procedures were conducted in another cohort of mice kept on a standard chow diet (CRM (P), 801722, Special Diets Services). Chow mice were individually housed in metabolic cages at 14–18 weeks of age and dissected 4 weeks later.

#### Plasma, media and tissue analyses of glutamine, glutamate and lactate

The glutamine-to-glutamate ratio in plasma, media and in iWAT was measured using the Glutamine/Glutamate-Glo Assay (Promega). Lactate measurements in snap-frozen iWAT samples were performed at The Swedish Metabolic Center (detailed in the targeted metabolic analysis below). Data were normalized by tissue weight.

#### Immunofluorescence analyses in murine WAT

As described in ref. ^54^, WAT samples were fixed in 4% PFA for 1 day, embedded in paraffin, cut into 5-μm sections and stained with hematoxylin and eosin (Sigma-Aldrich). For immunostaining, sections were rehydrated by successive baths in xylene, ethanol and PBS followed by blocking with 10% goat serum. The tissue sections were subsequently incubated overnight with antibodies directed against UCP1 (1:100), COX4 (1:100) and TOM20 (1:100). Goat anti-Rabbit Rhodamine Red-X (1:500) was used a as a secondary antibody and Hoechst 34580 (1:500) was applied for 20 min to counterstain nuclei. Images were acquired using a Axio Observer Z1 inverted fluorescence microscope (Zeiss) and the AxioVision software.

### Determination of fat cell size by image analysis

Qupath (v.0.3.4)^55^ was used to export histology images (tiles 2,048 × 2,048 pixels in .tiff format, 5× downsample). Fiji^56^ with the Adiposoft^57^ plugin (v.1.16) was used to quantify adipocyte size of whole tissue sections, pixel size was set to 1.2 μm, with expected diameter of 5–150 μm. An unpaired Student’s *t*-test was performed in R v.4.2.3 (R Core Team, v.2021) using the rstatix package.

### Single-nucleus RNA sequencing

#### Nuclei isolation and library preparation

BATs and WATs were collected from chow diet-fed *Gls*^*fl/fl*^ mice and *Gls*^*AdipoqCre*^ mice (*n* = 5 for each genotype) and promptly frozen in liquid nitrogen. Samples from each depot and genotype were pooled for nuclei isolation. Briefly, frozen tissue samples were minced and homogenized in cold lysis buffer using a gentleMACS Dissociator (Miltenyi Biotec). After addition of lysis buffer with Triton X-100 (X100, Sigma-Aldrich), the lysates were filtered and washed before centrifugation to collect the nuclei pellet. The isolated nuclei were stained with DAPI (D9542, Sigma-Aldrich), sorted, and counted before loading onto a 10X Chip G (10X Genomics). Libraries were prepared using the Chromium Single-Cell v.3.1 kit and sequenced on a Nextseq 2000 platform (Illumina).

#### Single-nucleus RNA sequencing data preprocessing

Raw sequencing files were processed using Cell Ranger v.7.0.1 based on the default parameters to demultiplex cell barcodes and generate cell-by-gene expression matrices. The mm10 mouse genome (refdata-gex-mm10-2020-A) from 10X genomics was used. We next applied SoupX v.1.6.2 (ref. ^58^) and DoubletFinder v.2.0.3 (ref. ^59^) to remove ambient RNA contamination and doublets, respectively. We filtered cells with more than 5% of mitochondrial RNA and excluded haemoglobin and mitochondrial genes, ribosomal protein families, *MTRNR* and *MALAT1* for downstream analyses.

#### Integration and cell type identification

We integrated the libraries on experimental conditions to remove batch effects using scVI v.0.16.2 (ref. ^60^). Briefly, all matrices were merged by Seurat v.4.1.3 (ref. ^61^), and a subset of the top 2,000 highly variable features was identified. From scVI, get_latent_representation was used for generating the latent embedding. By leveraging this latent embedding, we built a shared nearest neighbour graph, clustered cells and visualized all cells in a two-dimensional embedding by using Seurat’s FindNeighbors, FindClusters and RunUMAP. FindMarker was used for determination of differentially expressed genes. We annotated clusters through comparison of highly expressed genes of each cluster with well-established adipose cell type specific markers^62,63^.

### High-resolution respirometry in adipose tissues

#### Dissection and preparation of iWAT pieces

Inguinal WAT mitochondrial respiration was measured ex vivo using high-resolution respirometry following previously established methods (Oxygraph 2k, Oroboros)^64^. From *Gls*^*fl/fl*^ and *Gls*^*AdipoqCre*^ mice, iWAT was dissected, cleaned and cut in approximately 20 mg pieces. In total, 40 to 55 mg of the tissue was placed in 2 ml of ice-cold BIOPS buffer (2.77 mmol l^−1^ CaK_2_EGTA anhydrous, 7.23 mmol l^−1^ K_2_EGTA anhydrous, 5.77 mmol l^−1^ Na_2_ATP, 6.56 mmol l^−1^ MgCl_2_-6H_2_O, 20 mmol l^−1^ Taurine, 15 mmol l^−1^ Na_2_ phosphocreatine, 20 mmol l^−1^ Imidazole, 0.5 mmol l^−1^ eithiothreitol, 50 mmol l^−1^ MES) until the start of the assay. Excess of BIOPS buffer was then removed by blotting the tissue on filter paper before placing the samples in a respirometry chamber containing 2.1 ml of MIR05 buffer (0.5 mmol l^−1^ EGTA, 3 mmol l^−1^ MgCl_2_-6H_2_O, 60 mmol l^−1^ lactobionic acid, 20 mmol l^−1^ taurine, 10 mmol l^−1^ KH_2_PO_4_, 20 mmol l^−1^ HEPES, 110 mmol l^−1^ d-sucrose, 1 g l^−1^ fatty acid free bovine serum albumin).

#### High-resolution respirometry for inguinal white adipose

After adding the tissue into the respiration chamber, the chamber was closed and allowed to equilibrate before adding any substrates. Leak respiration associated with complex I was assessed by adding 2 mmol l^−1^ malate, 10 mmol l^−1^ pyruvate and 10 mmol l^−1^ glutamate. After O_2_ consumption rates were stabilized, ADP was added to a final concentration of 5 mmol l^−1^, triggering complex I-driven coupled respiration. After stabilization, complex II respiration was stimulated by adding 10 mmol l^−1^ succinate, obtaining the respiration rate corresponding to complex I + complex II-driven respiration. The maximal capacity of the electron transfer system (ETS) was assessed by titrating carbonyl cyanide-4-(trifluoromethoxy) phenylhydrazone in 0.5 μmol l^−1^ steps. Complex II-linked ETS capacity was evaluated by inhibiting complex I respiration with 0.5 µmol l^−1^ rotenone. Finally, 2.5 µmol l^−1^ antimycin A was added into the chamber, inhibiting complex III and therefore blocking mitochondrial electron circulation. The subsequent residual oxygen consumption—corresponding to non-mitochondrial respiration—was subtracted from the previous measured states. O_2_ consumption was normalized to the amount of tissue mass added in each assay. Mitochondrial membrane integrity and damage was assessed by adding cytochrome C after measuring complex I respiration. In our measurements, we did not observe differences in O_2_ consumption following this control step.

#### Dissection and preparation of BAT pieces

Interscapular BAT was dissected and separated from the surrounding white fat. Approximately 20 mg of the tissue was placed in a microcentrifuge tube containing 200 µl of MIR05 buffer and gently homogenized with a handheld pestle homogenizer. Then 20 µl (roughly 2 mg) of homogenate was added to the respiratory chamber containing 2.1 ml of MIR05 buffer.

#### High-resolution respirometry protocol for BAT

Uncoupled complex I- and complex I + complex II-driven mitochondrial respiration and maximal ETS capacity were evaluated by following the protocol described above for iWAT, minus the addition of ADP into the respirometry chamber.

### Cell studies

#### Differentiation and perturbations of human adipocytes

Isolation, proliferation and differentiation of human adipocyte progenitor cells (from an anonymous male donor, ethical permit no. 2009/764-32, regional ethics board of Stockholm) were performed as described^7^. Short interfering oligonucleotides (siRNAs) were transfected by electroporation using a Neon Transfection System (1,300 V, 20 ms, two pulses) using the 100 µl of Kit (Invitrogen) at day eight of adipocyte differentiation. All transfections were performed using a final concentration of 20 nmol l^−1^ siRNA oligonucleotides. Results were compared with non-silencing control siRNAs. A full list of RNA interference (RNAi) oligonucleotides is provided in Supplementary Table 2. In addition to the inhibitors used in the Seahorse assays, the cells were treated with the following chemicals (final concentrations and incubation times): dimethylsulfoxide (DMSO), BPTES (10 μmol l^−1^ for 3 h), CB-839 (10 μmol l^−1^ for 6 h), eta-ketoglutarate (2 mmol l^−1^ for 1 day), GPR81 agonist (500 nmol l^−1^ for 1 day), insulin (50 nmol l^−1^ for 15 min or 4 h), rapamycin (50 nm l^−1^ for 4 h), TNF (2 ng ml^−1^ for 4 h), STAT3 inhibitor VII (5 μmol l^−1^ for 5 h), NF-κB inhibitor (trifluoroacetate, 50 μg ml^−1^ for 5 h), JNK inhibitor SP600125 (10 μmol l^−1^ for 5 h), isoprenaline (10 μmol l^−1^ for 6 h), SB203580 (10 μmol l^−1^ for 24 h in RNAi experiments and a total of 3 h in lactate treatments including 2.5 h of preincubation), 2-deoxyglucose (100 μmol l^−1^ for 4 h), UK5099 (10 μmol l^−1^ for 4 h), pyruvate (5 mmol l^−1^ for 4 h), PF-739 (5 μmol l^−1^ for 1 day), sodium lactate (20 mmol l^−1^ for 30 min) and HIF Inhibitor VI (100 μmol l^−1^ for 4 h). For the glutamine depletion experiment, DMEM/F12 without glutamine was used either supplemented or not with 2.5 or 10 mmol l^−1^ of glutamine. All catalogue numbers and suppliers are detailed in Supplementary Table 2.

To generate cells with doxycycline-inducible *GLS* expression, the pCW-Cas9 plasmid (Addgene, no. 50661) was digested using BamHI-HF and NheI-HF and dephosphorylated using Calf Intestinal Alkaline Phosphatase according to the instructions from New England Biolabs (NEB). The digested plasmid was gel-purified using NucleoSpin Gel and PCR Clean-Up (Macherey-Nagel) and ligated overnight at 16 °C using T4 DNA ligase (NEB) with a codon-optimized sequence of *GLS*. The insert was generated by PCR from two distinct gBlocks as the sequence complexity was too high for synthesis (primers listed in Supplementary Table 2). Stable competent cells (*E. coli*, High efficiency, NEB, cat. no. C3040H) were transformed with the ligated plasmid according to the manufacturer’s instructions and single colonies were selected and cultured. From these, plasmids were extracted using QIAprep Spin Miniprep Kit (Qiagen) and verified by Sanger sequencing. Lentiviruses were created by transfecting human embryonic kidney 293 cells with this plasmid together with two additional packaging vectors (Addgene nos. 12259, 12260) using Opti-MEM and Lipofectamine 3000 (ThermoFisher) according to the manufacturer’s instructions. Viruses were collected from the conditioned media 2 days following transfection and filtered using Puradisc 25-mm 0.45-μm PES syringe filters (VWR). Around 200,000 proliferating adipocyte progenitor cells per well were seeded and cultured without antibiotics, then spinfected with 50,000 ng of virus for 1 h, 800*g* at 37 °C. Three days later, cells were incubated with 1 ng ml^−1^ puromycin. The selection was stopped when control cells in parallel wells were no longer viable. Induction of GLS was carried out by adding 2 ng ml^−1^ doxycycline to the cell culture media for at least 1 day.

To reintroduce GLS in siRNA-transfected adipocytes, *GLS* mRNA was in vitro transcribed using a DNA template encoding a codon-optimized coding sequence of *GLS* driven by a T7 promoter according to the HiScribe T7 ARCA mRNA Kit (with tailing, NEB no. E2060S). N1-Methylpseudo-UTP (cat. no. NU-890L, Saveen Werner) was added to the reaction to increase mRNA stability and thereby extend the intracellular expression. A total of 37 pmol of *GLS* mRNA was added in each Neon transfection (1,700 V, 20 ms, one pulse) with or without *GLS* siRNA as described above. Primers used to generate the DNA template are listed in Supplementary Table 2.

#### Mature adipocyte isolation and culture

Following dissection, WAT was subjected to careful washing and fine mincing. The minced tissue was thereafter placed in vials containing a Krebs–Ringer phosphate buffer (composed of 0.9% NaCl, 0.1 M NaH_2_PO_4_, 0.11 M CaCl_2_ and 0.154 M KCl, with a pH of 7.4) supplemented with 10% FBS, 5 mmol l^−1^ d-glucose, 1% penicillin-streptomycin (Thermo Fisher Scientific) and 0.05% collagenase type 1 (Sigma-Aldrich). The samples were placed in a shaking water bath set at 37 °C for a duration of 45 min. Subsequently, any connective tissue or substantial undigested fragments were effectively eliminated by filtration through a 250-µm nylon mesh. The resultant floating adipocytes underwent a series of four washes using PBS supplemented with 10% FBS, 1% penicillin-streptomycin and 5 mmol l^−1^ glucose (all from Sigma-Aldrich). The washed adipocytes were then poised for downstream analyses, either in their fresh state or following freezing at a temperature of −80 °C.

Human mature adipocytes were cultured within 6.5-mm Transwells (Costar-3413 and 3397), following a previously published protocol^65^. In brief, 30 µl of densely packed human adipocytes (approximately 60,000 cells) were carefully dispensed into each well. The Transwells were then inverted and positioned over a well of 24-well plates containing 1 ml of medium comprising DMEM/F12, 10% FBS and 1% penicillin-streptomycin. After a 4-h stabilization period, the cells were incubated with the following chemicals (final concentrations and incubation times): DMSO, CB-839 (10 μmol l^−1^ for 2 days) and TNF (10 ng ml^−1^ for 1 day).

#### RNA isolation, cDNA synthesis and qPCR

Total RNA was extracted from cells or intact human/murine WAT as described^7^. The RNA concentration and purity was measured using Varioskan Lux (ThermoFisher) and samples were reverse transcribed using iScript complementary DNA (cDNA) synthesis kits (BioRad). Messenger RNA levels were determined using TaqMan (Applied Biosystems) or SYBR-green (BioRad) assays and relative expression levels were calculated with the comparative Ct-method, that is, 2^ΔCt-target gene^/2^ΔCt-reference gene^. All primers are listed in Supplementary Table 2.

#### Western blot

Western blots were performed as described previously^7^ except that the membranes were scanned using a ChemiDoc MP system (BioRad). For experiments where the proteins of interest run at the same size, lysates were subdivided in equal amounts and loaded on separate gels as detailed in the respective figure legends. All antibodies used are listed in Supplementary Table 2.

#### Immunofluorescence analyses in human adipocytes

Cells were seeded on milli EZSlide-4 well slides and fixed in 4% PFA for 15 min at room temperature. Cells were washed twice with PBS, permeabilized using PBS supplemented with 0.2% Triton-X100 for 10 min and blocked for 30 min in PBS containing 2% bovine serum albumin (Sigma-Aldrich). The cells were subsequently incubated overnight with primary antibodies targeting UCP1 (1:100) or COX4 (1:100). The antibodies were diluted in PBS supplemented with 5% normal goat serum. Cells were washed three times with PBS containing 0.05% Tween-20 and incubated with goat anti-Rabbit Rhodamine Red-X secondary antibody (1:500) for 60 min. Subsequently PBS supplemented with Hoechst 34580 (1:500) and BODIPY 493/503 (1:2,500, ThermoFisher) was applied for 15 min to counterstain nuclei and lipid droplets, respectively. Finally, cells were washed in PBS and then mounted in fluorescence mounting medium (Fluoromount Aqueous Mounting Medium, refractive index 1.4). Images were obtained using an Axio Observer.Z1 inverted fluorescence microscope (Zeiss) and the AxioVision software.

#### Human adipocyte adiponectin secretion

For analyses of conditioned media from human adipocytes, samples were collected at day 14 of differentiation. Secretion of adiponectin was determined by ELISA (R&D Systems) according to the manufacturer’s instructions.

#### Redox balance measurements

Cultured si*C* or si*GLS* adipocytes were collected at day 14 of differentiation. For analyses of NAD^+^ and NADH, the NAD^+^/NADH Assay Kit (cat. no. ab65348) was used according to the manufacturer’s instructions. Data were normalized by protein concentrations per sample.

#### Seahorse assays and CyQUANT analyses

Real-time measurements of oxygen consumption and ECARs were performed using a Seahorse XF96 Extracellular Flux Analyzer (Agilent Technologies) as previously described^54^. In brief, adipocytes were incubated in Seahorse DMEM medium (pH 7.4) supplemented with 1 mmol l^−1^ pyruvate, 2 mmol l^−1^ glutamine and 10 mmol l^−1^ glucose. The assays were performed by sequential addition of 1.5 μmol l^−1^ oligomycin (inhibitor of ATP synthesis), 1.5 μmol l^−1^ carbonyl cyanide-p-trifluoromethoxyphenylhydrazone and 0.5 μmol l^−1^ rotenone/antimycin A (inhibitors of complex I and complex III of the respiratory chain, respectively). To assess responses to glucose addition during glycostress tests, cells were incubated in medium with 2 mmol l^−1^ glutamine but without glucose and pyruvate. The assays were performed by sequential addition of 10 mmol l^−1^ glucose, 1 μmol l^−1^ oligomycin and 50 mmol l^−1^ 2-deoxyglucose. Mitochondrial fuel stress tests were performed using the XF96 fuel kit after acute injection of UK5099 (2 μmol l^−1^ per well) or Etomoxir (4 μmol l^−1^ per well). Seahorse data were normalized using the CyQUANT Kit (ThermoFisher) according to the manufacturer’s instructions. Immediately after Seahorse analysis, the cells were incubated with the CyQUANT reagent and fluorescence was measured. For estimation of basal and maximal respiration, the mean non-mitochondrial respiration was subtracted from the mean values of basal and maximal respiration. For glycostress tests, the mean non-glycolytic acidification was subtracted from all data points.

#### RNA sequencing and pathway analyses

Total RNA from si*C* and si*GLS* cells was isolated as described above and used for library preparation. The yield and quality of the amplified libraries were analysed using Qubit (Thermo Fisher) and Tapestation (Agilent) and subsequently normalized and combined. These pools were sequenced on the Illumina Nextseq 2000 P2 100 cycle sequencing run, generating 59 base paired end reads with dual index. Basecalling and demultiplexing was performed using CASAVA software with default settings generating Fastq files aligned to GRCh38 for further downstream mapping and analysis. Raw counts were normalized and analysed using DESeq2 in R studio (v.4.1.1). Pathway analyses were performed using GSEA (gene set enrichment analysis) and ProFat^18^.

#### GLS activity assay

In human and mouse adipocytes, GLS activity was determined using the Glutaminase Assay Kit (Abcam). In brief, samples were homogenized on ice in 1 ml of assay buffer and subjected to 8,000*g* centrifugation for 10 min at 4 °C. Protein concentrations were determined by Pierce BCA assay (Thermo Scientific). Samples containing equal amounts of protein were incubated with kit reagents at 37 °C, and fluorescence was detected over time using a Varioskan Lux. Control samples exposed to the kit reagent mixture lacking GLS substrate were used to measure endogenous basal glutamate levels. GLS activity was calculated on the basis of the increase in glutamate over time. Data were normalized by protein concentration for each sample.

#### Targeted glutamine and glutamate measurements

Glutamine and glutamate levels in adipocytes were determined using the Glutamine/Glutamate-Glo Assay (Promega). One half of the volume of the lysed cells was used to measure glutamate and the other half was used to measure glutamine. Protein concentrations were determined by Pierce BCA assay (Thermo Scientific) and samples were normalized to total protein content.

#### Chromatin immunoprecipitation

Chromatin immnuprecipiation against c-Jun was performed using Magna ChIP HiSense Chromatin Immunoprecipitation Kit (cat. no. 17-10460, Sigma), following the manufacturer’s instructions. The antibody targeting c-Jun (cat. no. 9165, Cell Signalling), was added at the concentration of 1:50, as suggested by the manufacturer. A negative control was generated using IgG Ab (cat. no. 7074S, Cell Signalling). Pull down enrichment quantification was performed using normal qPCR with reverse transcription as above described with the primer set in Supplementary Table 2. Relative enrichment was calculated using input enrichment ((Ct Input − log_2_100) → 100 × 2^(Adjusted input−Ct IP)^) and fold change against IgG signal (Ct IP/Ct IgG).

#### Adipocyte U-^13^C glucose labelling

Cells were washed twice with PBS and thereafter incubated with DMEM without glucose (cat. no. 11966025, Thermo Fisher) supplemented with an addition of 5.5 mM glucose (cat. no. G8270, Sigma) or U-13C6 d-Glucose (cat. no. 389374,Sigma) for 4 h. Cells were washed with cold PBS twice before being lysed in 90% ice-cold methanol (cat. no. 34860-1L-R, Sigma). Before cell collection, 1 ml of the media was extracted and centrifuged at 21,000*g* for 10 min at 4 °C then frozen. One millilitre of ice-cold 90% methanol was added to facilitate subsequent metabolic tracing analysis.

#### Lactate measurements

Lactate was measured in cell media using the Lactate-Glo Assay (Promega). Data were normalized by protein concentration per well.

#### Targeted metabolite analyses

Metabolites were profiled at the Swedish Metabolomics Centre. To trace U-^13^C-glutamine conversion, human adipocytes were treated with 10 μmol l^−1^ of BPTES or vehicle (DMSO) and 2.5 mmol l^−1^ U-^13^C-glutamine (Sigma-Aldrich) for 1 h. The cell, tissue and media samples were extracted with 1 ml of 90% methanol (diluted in water). Two aliquots of the extracts were analysed by (1) gas-chromatography-mass spectrometry (GC–MS) and (2) liquid chromatography–mass spectrometry (LC–MS). For GC–MS measurements, derivatization and GC–MS analysis were performed as described previously^66,67^. The extracted mass spectra were annotated (putatively/tentatively identified) by library comparisons of their retention index and mass spectra^68^. Mass spectra and retention index comparison was performed using NIST MS v.2.2 software, and annotation of mass spectra was based on reverse and forward searches. Both the Swedish Metabolomics Centre’s in-house standards libraries and public libraries as NIST (https://chemdata.nist.gov/), MoNA (https://mona.fiehnlab.ucdavis.edu/) and MS-DIAL (https://systemsomicslab.github.io/compms/msdial/main.html) was used. Peak detection and peak area calculations of both labelled and unlabelled fragments was done as described in ref. ^69^

The LC–MS analysis of amino acids was conducted using ultrahigh-performance liquid chromatography wtih electrospray ionizaton and quadrupole time of flight after the amino acids were derivatized with AccQ-Tag (Waters). The derivatized amino acids were identified on the basis of their retention time and exact mass. The acquired LC–MS data were converted to XML and eventually to NetCDF format, then the peaks integration and labelling calculation were conducted as described^41^.

### Statistical analyses

Unless otherwise stated, results are reported as mean ± standard error of the mean (s.e.m.) with individual data points shown for experiments with fewer than ten replicates per sample group. The number of independent experiments and relevant statistical methods (all two-sided) for each panel are detailed in the corresponding figure legends. Statistical analyses were performed using Prism (v.9.2.0, GraphPad Software), JMP (v.15.1, SAS) or R v.4.1.1./4.2.1.

### Reporting summary

Further information on research design is available in the Nature Portfolio Reporting Summary linked to this article.

### Supplementary information

Supplementary Information Supplementary Tables 1 and 2.

Reporting Summary

### Source data

Source Data Fig. 1 Source data for Fig. 1.

Source Data Fig. 2 Source data for Fig. 2.

Source Data Fig. 3 Source data for Fig. 3.

Source Data Fig. 4 Source data for Fig. 4.

Source Data Fig. 5 Source data for Fig. 5.

Source Data Fig. 6 Source data for Fig. 6.

Source Data Fig. 7 Source data for Fig. 7.

Source Data Fig. 8 Source data for Fig. 8.

Source Data Figs. 1–8 and Extended Data Figs. 1–7 Source western blots in main Figs. 1–8 and Extended Data Figs. 1–7.

Source Data Extended Data Fig. 1 Source data.

Source Data Extended Data Fig. 2 Source data.

Source Data Extended Data Fig. 3 Source data.

Source Data Extended Data Fig. 4 Source data.

Source Data Extended Data Fig. 5 Source data.

Source Data Extended Data Fig. 6 Source data.

Source Data Extended Data Fig. 7 Source data.

## Data Availability

Data generated or retrospectively analysed in this study are publicly available in the National Center for Biotechnology Information Gene Expression Omnibus repository under the accession numbers GSE25402 (transcriptomics of cohort 3) and GSE267800 (transcriptomics of si*GLS* versus si*C* transfected cells and single-nucleus RNA sequencing of *Gls*^*AdipoqCre*^ and control mice). Metabolomics and clinical data from cohort 1 are provided in Petrus et al.^7^ and Maqdasy et al.^54^. Additional data that support the findings of this study are available from the corresponding authors (N.M. and M.R.). Full individual clinical data are not publicly available due to them containing information that could compromise research participant privacy or consent. Source data are provided with this paper.

## Code availability

Scripts used for the analyses presented in this study are available at GitHub (https://github.com/lmassier/GLS).
