# Supplementary material for: Reduced adipocyte glutaminase activity promotes energy expenditure and metabolic health
Source: Nat Metab. 2024 Jul 15;6(7):1329–46. doi: 10.1038/s42255-024-01083-y (PMC11272588; doi:10.1038/s42255-024-01083-y)
Supplement: Supplementary file 1 — Supplementary Tables 1 and 2. [file 42255_2024_1083_MOESM1_ESM.pdf]

nature metabolism

Supplementary information

https://doi.org/10.1038/s42255-024-01083-y

Reduced adipocyte glutaminase activity promotes energy expenditure and metabolic health

In the format provided by the authors and unedited

Supplementary Table 1

Cohort 1 metabolomics in human plasma			
**Parameters**	**Non-obese (n=23)**	**Obese (n=30)**	**p-value**
Age (years)	44.5 +/- 11.3	42.4 +/- 8.7	N.S
BMI (kg/m^2)	24.3 +/- 2.3	37.4 +/- 3.1	p<0.0001
Insulin sensitivity index (M/I ratio)	4.1 +/- 1.6	2 +/- 0.99	p<0.0001

Cohort 1 metabolomics in WAT			
**Parameters**	**Non-obese (n=13)**	**Obese (n=13)**	**p-value**
Age (years)	43.5 +/- 14.2	44.1 +/- 8.5	N.S
BMI (kg/m^2)	22.9 +/- 2.3	38.3 +/- 2	p<0.0001
Insulin sensitivity index (M/I ratio)	5.1 +/- 1.6	1.9 +/- 0.8	p<0.0001

Cohort 2 human mature adipocytes			
**Parameters**	**Non-obese (n=12)**	**Obese (n=16)**	**p-value**
Sex ratio (male vs female)	4 vs 8	2 vs 14	
Age (years)	35.6 +/- 13.2	41 +/- 11.2	N.S
BMI (kg/m^2)	22 +/- 2.4	47.6 +/- 11.1	p<0.0001

Supplementary Table 2. List of reagents.

Reagents and	SOURCE	IDENTIFIER	Dilution
Lamin A/C	Cell Signaling Tech.	Cat#4777	WB: 1/1000
α-Tubulin	Cell Signaling Tech.	Cat#2144	WB: 1/1000
Calnexin	Cell Signaling Tech.	Cat#2679S	WB: 1/1000
GAPDH (14C10)	Cell Signaling Tech.	Cat#2118	WB: 1/1000
GLUTAMINASE [EP7212] (HRP)	Abcam	Cat#ab200408	WB: 1/2500
UCP-1	Abcam	Cat#AB10983	WB: 1/1000<br>IF: 1/100
Total OXPHOS	Abcam	Cat#AB110413	WB: 1/1000
FABP4	Sigma-Aldrich	Cat#SAB1408986	WB: 1/1000
PPAR gamma (C26H12)	Cell Signaling Tech.	Cat#2435	WB: 1/1000
PLIN1	Cell Signaling Tech.	Cat#9349	WB: 1/1000
Adiponectin	Thermo Fisher	Cat#MA1-054	WB: 1/1000
C/EBPα (D56F10)	Cell Signaling Tech.	Cat#8178	WB: 1/1000
Goat α-Rabbit IgG (H+L)	Thermo Fisher	Cat#R-6394	1/500
LCA, Rhodamine	Vector lab	Cat#RL-1042	1/500
Rabbit IgG-HRP	Sigma-Aldrich	Cat#A9169	WB: 1/10,000
Mouse IgG-HRP	Sigma-Aldrich	Cat#5278	WB: 1/10,000
AMPK	Cell Signaling Tech.	Cat#2532S	WB: 1/1000
pAMPK	Cell Signaling Tech.	Cat#2535L	WB: 1/1000
S6 Ribosomal Protein (5G10)	Cell Signaling Tech.	Cat#2217	WB: 1/1000
Phospho-S6 Ribosomal Protein (Ser240/244)	Cell Signaling Tech.	Cat#2215	WB: 1/1000
P70S6K	Cell Signaling Tech.	Cat#2708S	WB: 1/1000
pP70S6K	Cell Signaling Tech.	Cat#9205S	WB: 1/1000
P38 MAPK	Cell Signaling Tech.	Cat#9212	WB: 1/1000
pP38 MAPK	Cell Signaling Tech.	Cat#9211	WB: 1/1000
ATF-2	Cell Signaling Tech.	Cat#35031S	WB: 1/1000
pATF-2	Cell Signaling Tech.	Cat#27934S	WB: 1/1000
CREB	Cell Signaling Tech.	Cat#9197S	WB: 1/1000
pCREB	Cell Signaling Tech.	Cat#9198S	WB: 1/1000
COX VI	Abcam	Cat#ab16056	IF: 1/100
TOM20			IF: 1/100
c-Jun	Cell Signaling Tech.	Cat#9165S	WB: 1/1000;<br>ChIP: 1/50
p-c-Jun	Cell Signaling Tech.	Cat#3270S	WB: 1/1000
STAT3	Cell Signaling Tech.	Cat#9139S	WB: 1/1000
pSTAT3	Cell Signaling Tech.	Cat#9145T	WB: 1/1000
P65	Cell Signaling Tech.	Cat#3036S	WB: 1/1000
pP65	Cell Signaling Tech.	Cat#3033T	WB: 1/1000
HIF1α	Cell Signaling Tech.	Cat#14179S	WB: 1/500
Goat serum	Sigma-Aldrich	Cat#G9023	5%

Gene silencers		
ON-TARGETplus Non-targeting Pool	Dharmacon	Cat# D-001810-01-20
ON-TARGETplus siRNA GLS- Human	Dharmacon	Cat#L-004548-01-0005
ON-TARGETplus siRNA UCP1- Human	Dharmacon	Cat#LL-007636-00-0005

Critical Commercial Assays		
Seahorse XF Glycolysis Stress Test	Agilent	Cat#103020-100
Seahorse XF Mito Stress Test	Agilent	Cat#103015-100
Seahorse XFp Mito Fuel Flex Test	Agilent	Cat#103070-100
iScript cDNA Synthesis kit	Bio-Rad	Cat#170-8891
Pierce BCA Protein Assay Kit	ThermoFisher	Cat#23227
Mouse/Rat Leptin Quantikine ELISA Kit	R&D Systems	Cat#MOB00B
PicoProbeTM Glutaminase (GLS) Activity Assay Kit	BioVision	Cat#K455-100
Human Total Adiponectin/Acrp30 Quantikine ELISA	R&D Systems	Cat#DRP300
Glutamine/Glutamate-Glo kit	Promega	Cat#J8021
Lactate-Glo assay	Promega	Cat#J5021
NAD+/NADH	Abcam	Cat#ab65348

Chemicals, Peptides, and Recombinant Proteins		
SYBR-green Master Mix	Bio-Rad	Cat#1708884
TaqMan Master Mix	Applied Biosystems	Cat#4318157
Amersham ECL Prime Blocking Reagent	GE Healthcare	Cat#RPN418
Amersham ECL Prime Western Blotting Detection Reagent	GE Healthcare	Cat#RPN2232
DTT	Sigma-Aldrich	Cat#10197777001
QIAzol lysis reagent	Qiagen	Cat#79306
Bodipy 493/503	ThermoFisher	Cat# D3922
Hoechst	ThermoFisher	Cat#H21486
CyQUANT	ThermoFisher	Cat#C7026
Glucose Solution	ThermoFisher	Cat#A2494001
RIPA buffer	ThermoFisher	Cat#89901
Collagenase	Sigma-Aldrich	Cat#C6885
FGF2 human	Sigma-Aldrich	Cat#F0291
Insulin (liquid, ready to use, 10mg/ml, 5ml)	Sigma-Aldrich	Cat#I9278
T3	Sigma-Aldrich	Cat#T6397
Transferrin	Sigma-Aldrich	Cat#T8158
IBMX (3-Isobutyl-1-methylxanthin)	Sigma-Aldrich	Cat#I5879
Rosiglitazone (100 mg)	Cayman Chemicals	Cat#71740
Dexamethasone	Sigma-Aldrich	Cat#D1756
0.5% Trypsin/EDTA (10X)	Invitrogen (GIBCO)	Cat#15400-054
Bovine serum albumin	Sigma-Aldrich	Cat#A7030
Penicilline G 10000U/ml/Streptomycine 10000µg/ml	Invitrogen (GIBCO)	Cat#15140-122
Hepes 1M	Invitrogen (GIBCO)	Cat#15630-056
Ham’s F-12 Nutrient Mix	Invitrogen (GIBCO)	Cat#21765-037
DMEM, no glucose	ThermoFisher (GIBCO)	Cat#11966025
Pierce™ 16% Formaldehyde (w/v), Methanol-free	ThermoFisher	Cat#28906
4x Laemmli Sample Buffer	BioRad	Cat#1610747
L-Glutamine-13C5,98 atom % 13C, 95% (CP)	Sigma-Aldrich	Cat#605166
D-Glucose-$^{13}C_6$	Sigma-Aldrich	Cat# 389374
UK-5099	Sigma-Aldrich	Cat#PZ0160
EtaKG	Sigma-Aldrich	Cat#SML1743-5MG
PF-739	Aobious	Cat#AOB33584
Rapamycin	Sigma-Aldrich	Cat#R8781-200UL
L-Glutamine	Cytiva	Cat#SH30034.01
BPTES	Sigma-Aldrich	Cat#SML0601
CB-839	MedChemExpress	Cat#HY-12248
GPR81 agonist 1	MedChemExpress	Cat#HY-135982

SB203580	Sigma-Aldrich	Cat#S8307
HIF1α inhibitor VI	Sigma-Aldrich	Cat#400086
2-deoxy-D-glucose	Sigma-Aldrich	Cat#D6134-1G
2-deoxy-D- [1-H^3]-glucose	Perkin Eimer	Cat#NET328250UC
STAT3 Inhibitor VII	Sigma-Aldrich	Cat#573103
JNK inhibitor VIII	Sigma-Aldrich	Cat#420135
NF-κB inhibitor	Cayman	Cat#17493
Sodium pyruvate	Sigma-Aldrich	Cat#P2256
Oligomycin	Agilent	Cat#103015-100
Seahorse Glutamine solution	Agilent	Cat#103579-100
Seahorse glucose solution	Agilent	Cat#103577-100
Seahorse pyruvate solution	Agilent	Cat#103578-100

Oligonucleotides		
TaqMan probe human *LRP10*	Applied Biosystems	Hs00204094_m1
TaqMan probe human *PPIA*	Applied Biosystems	Hs04194521_s1
TaqMan probe human *HPRT1*	Applied Biosystems	Hs99999909_m1
TaqMan probe human *GLS*	Applied Biosystems	Hs01014020_m1
TaqMan probe human *PLIN1*	Applied Biosystems	Hs00193510_m1
TaqMan probe human *PPARG*	Applied Biosystems	Hs01115513_m1
TaqMan probe human *CEBPA*	Applied Biosystems	Hs00269972_s1
TaqMan probe human *ADIPOQ*	Applied Biosystems	Hs00605917_m1
TaqMan probe human *CO1*	Applied Biosystems	Hs02596864_g1
TaqMan probe human *NDUFB5*	Applied Biosystems	Hs00159582_m1
TaqMan probe human *GLUL*	Applied Biosystems	Hs00365928_g1
TaqMan probe murine *Actin B*	Applied Biosystems	Mm00607939_s1
TaqMan probe murine *Gapdh*	Applied Biosystems	Mm99999915_g1

Cloning primers		
ID	Name	Sequence
GL019	GLS_N_term_Fwd	CTGTCTGGTAGCTAGCGCCACCATGCATCACCATCACCATCAC
GL020	GLS_N_term_Rev	CGCGCTATCAGACAGGGATCCCTAGAGCAAACCGTCCAGAT
GL021	GLS_C_term_Fwd	TGGTAGCTAGCGCCATGATGCGCCTTCGAGGGTCAG
GL022	GLS_C_term_Rev	CGCGCTATCAGACAGGGATCCCTAGTGATGGTGATGGTGA

mRNA synthesis		
ID	Name	Sequence
GL226	His-GLS T7 mRNA Fwd	ACACTAATACGACTCACTATAGGGGCCACCATGCATCACCATCACCATCACATGA
GL227	His-GLS T7 mRNA Rev	CTAGAGCAAACCGTCCAGATTCTTGTGCAC

qPCR primers		
ID	Name	Sequence
GL033	GLS_OE_Fwd	TGGCCGGTAACGAGTATGTG
GL034	GLS_OE_Rev	GCAGAAGCCTCCATTAGCCA
ChIP	GLS promoter_Fwd	5’-GCGTGCAGAAAGTGGCTACTGAGC-3’
ChIP	GLS promoter_Rev	5’-CTCTCGGCTCTGGGTGCGCGGAGAG-3’
qPCR	*Ucp1*_mm_Fwd	5´-ACTGCCACACCTCCAGTCATT-3´

qPCR	*Ucp1*_mm_Rev	5´-CTTTGCCTCACTCAGGATTGG-3´
qPCR	*Ppargc1a*_mm_Fwd	5´-TTCCCCATTTGAGAACAAGA-3´
qPCR	*Ppargc1a*_mm_Rev	5´-GGAGGAGTTGTGGGAGGAGT-3´
qPCR	*Prdm16*_mm_Fwd	5´-GCACGGTGAAGCCATTCATATG-3´
qPCR	*Prdm16*_mm_Rev	5´-TCGGCGTGCATCCGCTTGTG-3
qPCR	*Cox8b_mm_Fwd*	5´-GAACCATGAAGCCAACGACT-3´
qPCR	*Cox8b_mm_Rev*	5´-GCGAAGTTCACAGTGGTTCC-3´
qPCR	*Dio2_mm_Fwd*	5´-CATTGATGAGGCTCACCCTTC-3´
qPCR	*Dio2_mm_Rev*	5´-GGTTCCGGTGCTTCTTAACCT-3
qPCR	*Rps18_mm_Fwd*	5´-CATGCAAACCCACGACAGTA-3´
qPCR	*Rps18_mm_Rev*	5´-CCTCACGCAGCTTGTTGTCTA-3´
qPCR	*Rplp0_mm_Fwd*	5´-TCCAGGCTTTGGGCATCA-3´
qPCR	*Rplp0_mm_Rev*	5´-CTTTATCAGCTGCACATCACTCAGA-3´
qPCR	*Glul_mm_Fwd*	5´-TTAGGGGAATTAAGGACAGG-3
qPCR	*Glul_mm_Rev*	5-GAAGGAAAGTAACAGTCTGC-3´
qPCR	*MT-CYB_ Fwd*	5’-TCATCGACCTCCCCACCCCATC-3´
qPCR	*MT-CYB_ Rev*	5’-CGTCTCGAGTGATGTGGGCGATT-3´
qPCR	*SDHB_ Fwd*	5’-ACAGCTCCCCGTATCAAGAAA-3´
qPCR	*SDHB_ Rev*	5’-GCATGATCTTCGGAAGGTCAA-3´
qPCR	*Gls_ Fwd*	5'-ATATTCCTCAGCTGGCCAAA-3´
qPCR	*Gls_ Rev*	5'-CTTGGCTCCTTCCCAACATA-3´
qPCR	*Ndufs7_Fwd*	5´-CTTCTGTTCACGCTTGATCTTC
qPCR	*Ndufs7_ Rev*	5´-GGCTACTACCACTACTCCTACT
qPCR	*Cox5b_ Fwd*	5'- GCGAAGTAACCTTGAAGCCA
qPCR	*Cox5b_ Rev*	5'- CCGCCCATCTTGCTCAG
qPCR	*Cox4i1_ Fwd*	5´-GCAGACAGCATCGTGACAT
qPCR	*Cox4i1_ Rev*	5´-GAGAGCCATTTCTACTTCGGT
qPCR	*Cox8a_ Fwd*	5´-CTTCGAGTGGACCTGAGC
qPCR	*Cox8a _ Rev*	5´-CATCTTGACTCCCTGACCTTG
qPCR	*Atp5d_ Fwd*	5´-AAGATGCCAAAGGCTCCAG
qPCR	*Atp5d_ Rev*	5´-GATGTCCTTCACCTTTGCCT
qPCR	*Slc1a2_ Fwd*	GCAGTTCGGCTATAACACTGG
qPCR	*Slc1a2_ Rev*	GCGGTGGTTCCATGTTTGATTG
qPCR	*Pdk1_Fwd*	GGACTTCGGGTCAGTGAATGC
qPCR	*Pdk1_Rev*	TCCTGAGAAGATTGTCGGGGA
qPCR	*Pdhb_Fwd*	GTGGAAGAAATACGGTGACAAGA
qPCR	*Pdhb_Rev*	ACCTGGTCAATAGCTTGCATAGA
qPCR	*Ldha_Fwd*	CAAAGACTACTGTGTAACTGCGA
qPCR	*Ldha_Rev*	TGGACTGTACTTGACAATGTTGG
qPCR	*Angptl14_Fwd*	CATCCTGGGACGAGATGAACT
qPCR	*Angptl14_Rev*	TGACAAGCGTTACCACAGGC
qPCR	*Vegfa_Fwd*	ACATTGGCTCACTTCCAGAAACAC
qPCR	*Vegfa_Rev*	GGTTGGAACCGGCATCTTTATC
qPCR	*Cre_Fwd*	CCCTGTTTCACTATCCAGGT

qPCR	*Cre_Rev*	GGGTAACTAAACTGGTCGAG

		gblocks
ID	Name	Sequence
cGL004	His_GLS_TAG first half with overhangs	ctgtctggtagctagcgccaccATGCATCACCATCACCATCACATGATGCGCCT<br>TCGAGGGTCAGGTATGCTTCGCGATCTCTTGCTTCGGAGCCCAGCA<br>GGTGTCAGTGCGACTCTTAGAAGGGCTCAGCCTCTCGTCACTCTCT<br>GTAGAAGGCCTAGAGGCGGAGGACGACCAGCGGCGGGACCTGCT<br>GCTGCCGCCAGACTTCATCCTTGGTGGGGAGGAGGAGGATGGCCA<br>GCTGAACCTCTGGCAAGGGGTCTCTCTTCTTCCCCCAGCGAGATAC<br>TGCAAGAACTGGGTAAGGGGTCCACTCATCCTCAGCCAGGGGTCTC<br>ACCTCCAGCCGCTCCTGCAGCCCCTGGCCCTAAAGATGGGCCTGG<br>AGAGACAGACGCCTTTGGAAACAGTGAAGGCAAGGAGCTCGTTGCT<br>TCCGGGGAGAATAAAATTAAACAAGGACTCTTGCCTAGTCTCGAGG<br>ATTTGCTCTTCTATACGATTGCAGAAGGACAGGAAAAGATCCCTGTG<br>CATAAATTTATCACCGCTTTGAAGAGTACAGGATTGCGCACCAGTGA<br>CCCTCGGCTCAAGGAGTGTATGGACATGCTGCGGTTGACGCTTCAA<br>ACTACCTCCGATGGAGTGATGCTTGACAAAGATCTGTTCAAGAAGTG<br>TGTCCAGAGTAACATAGTACTGCTTACCCAGGCTTTCAGACGAAAGT<br>TTGTGATACCGGATTTCATGTCTTTCACCTCACACATTGACGAGCTC<br>TACGAGAGCGCCAAGAAGCAAAGTGGGGGTAAGGTTGCCGATTATA<br>TACCTCAACTGGCTAAATTTAGCCCAGACCTTTGGGGCGTGAGTGTT<br>TGTACGGTAGACGGACAACGCCATTCTACAGGTGACACTAAGGTAC<br>CTTTCTGCTTGCAATCTTGCGTAAAACCTCTTAAATACGCTATCGCA<br>GTTAATGACCTTGGTACAGAATATGTACATCGATATGTGGGGAAGGA<br>GCCTTCCGGGTTGCGATTCAACAAGCTCTTTCTGAATGAGGACGATA<br>AGCCTCATAACCCTATGGTCAATGCCGGGGCTATAGTAGT
cGL005	GLS_His-TAG second half no overhangs	AATGCCGGGGCTATAGTAGTTACTTCACTCATCAAGCAGGGTGTAAA<br>TAATGCTGAGAAGTTCGATTACGTTATGCAATTCTTGAACAAGATGG<br>CCGGTAACGAGTATGTGGGATTTTCTAATGCAACCTTTCAAAGTGAG<br>CGAGAGTCAGGTGATCGAAACTTTGCCATAGGTTACTACCTTAAGGA<br>GAAGAAGTGCTTCCCCGAGGGAACAGACATGGTAGGAATACTTGAC<br>TTTTACTTTCAGCTGTGTTCTATCGAGGTTACCTGCGAATCCGCTTCT<br>GTTATGGCTGCGACGTTGGCTAATGGAGGCTTCTGCCCTATAACCG<br>GCGAGCGGGTATTGTCTCCAGAAGCGGTACGCAATACGCTTAGCCT<br>TATGCACTCCTGCGGGATGTATGATTTCAGTGGTCAATTTGCTTTCC<br>ATGTGGGGCTGCCCGCCAAGTCTGGTGTGGCAGGAGGCATCCTTTT<br>GGTAGTGCCTAACGTCATGGGGATGATGTGCTGGTCACCGCCATTG<br>GATAAGATGGGTAATTCTGTTAAGGGGATTCATTTCTGCCATGACCT<br>TGTTTCTCTCTGTAATTTTCACAATTATGACAACCTTCGACACTTTGC<br>GAAGAAGTTGGACCCTAGGCGAGAGGGCGGCGATCAACGCGTTAA<br>ATCCGTCATTAACCTCCTCTTTGCGGCTTACACGGGTGACGTCAGTG<br>CTCTCCGACGATTCGCGCTGAGCGCTATGGACATGGAACAAAGAGA<br>TTATGACAGTCGCACAGCTTTGCATGTCGCTGCTGCTGAAGGACAC<br>GTTGAAGTGGTAAAGTTTCTTTTGGAAGCCTGCAAGGTGAATCCCTT<br>CCCTAAGGATCGATGGAACAATACTCCGATGGACGAGGCACTCCAT<br>TTCGGTCATCATGACGTTTTTAAAATACTGCAAGAGTATCAGGTCCA<br>ATATACACCTCAGGGCGACTCTGATAACGGAAAAGAAAACCAGACA<br>GTGCACAAGAATCTGGACGGTTTGCTCCATCACCATCACCATCACTA<br>Gggatccctgtctgata

Software, Algorithms and others		
Image Lab	Bio-Rad	N/A
GraphPad Prism 7.0	GraphPad Software	N/A
RStudio	GNU	N/A
ImageJ 1.45 software	NIH	N/A
Milli EZSlide 4-well Glass Slide	Merck	Cat#PEZGS0416
