# Supplementary material for: Reduced adipocyte glutaminase activity promotes energy expenditure and metabolic health
Source: Nat Metab. 2024 Jul 15;6(7):1329–46. doi: 10.1038/s42255-024-01083-y (PMC11272588; doi:10.1038/s42255-024-01083-y)
Supplement: Supplementary file 2 — Reporting Summary [file 42255_2024_1083_MOESM2_ESM.pdf]

nature portfolio

Corresponding author(s): Rydén and Mejhert

Last updated by author(s): May 28, 2024

Reporting Summary

Nature Portfolio wishes to improve the reproducibility of the work that we publish. This form provides structure for consistency and transparency in reporting. For further information on Nature Portfolio policies, see our Editorial Policies and the Editorial Policy Checklist.

Statistics

For all statistical analyses, confirm that the following items are present in the figure legend, table legend, main text, or Methods section.

n/a	Confirmed	
☐	☒	The exact sample size (*n*) for each experimental group/condition, given as a discrete number and unit of measurement
☒	☐	A statement on whether measurements were taken from distinct samples or whether the same sample was measured repeatedly
☐	☒	The statistical test(s) used AND whether they are one- or two-sided<br>*Only common tests should be described solely by name; describe more complex techniques in the Methods section.*
☐	☒	A description of all covariates tested
☒	☐	A description of any assumptions or corrections, such as tests of normality and adjustment for multiple comparisons
☐	☒	A full description of the statistical parameters including central tendency (e.g. means) or other basic estimates (e.g. regression coefficient) AND variation (e.g. standard deviation) or associated estimates of uncertainty (e.g. confidence intervals)
☐	☒	For null hypothesis testing, the test statistic (e.g. *F*, *t*, *r*) with confidence intervals, effect sizes, degrees of freedom and *P* value noted<br>*Give P values as exact values whenever suitable.*
☒	☐	For Bayesian analysis, information on the choice of priors and Markov chain Monte Carlo settings
☒	☐	For hierarchical and complex designs, identification of the appropriate level for tests and full reporting of outcomes
☐	☒	Estimates of effect sizes (e.g. Cohen's *d*, Pearson's *r*), indicating how they were calculated

Our web collection on statistics for biologists contains articles on many of the points above.

Software and code

Policy information about availability of computer code

Data collection	No software was used for data collection
Data analysis	Image Lab 6.0.1<br>GraphPad Prism 7.0 and 9.5.0<br>RStudio v4.1.1 and v4.2.1<br>ImageJ 1.45 software<br>NIST MR 2.2<br>CASAVA 1.8.2<br>JMP v15.1<br>Scripts used for the analyses presented in this study are available at GitHub (https://github.com/lmassier/GLS).

For manuscripts utilizing custom algorithms or software that are central to the research but not yet described in published literature, software must be made available to editors and reviewers. We strongly encourage code deposition in a community repository (e.g. GitHub). See the Nature Portfolio guidelines for submitting code & software for further information.

Data

Policy information about availability of data

All manuscripts must include a data availability statement. This statement should provide the following information, where applicable:

- Accession codes, unique identifiers, or web links for publicly available datasets
- A description of any restrictions on data availability
- For clinical datasets or third party data, please ensure that the statement adheres to our policy

Data generated or retrospectively analyzed in this study are publicly available in the NCBI Gene Expression Omnibus repository under the accession numbers GSE25402 (transcriptomics of cohort 3) and GSE267800 (transcriptomics of siGLS vs. siC transfected cells and snSeq of GlsAdipoqCre and control mice). Metabolomics data from cohort 1 are provided in Petrus et al. and Maqdasy et al. (refs 7 and 54 in the manuscript) Source data for western blots are provided with this paper.

Research involving human participants, their data, or biological material

Policy information about studies with human participants or human data. See also policy information about sex, gender (identity/presentation), and sexual orientation and race, ethnicity and racism.

Reporting on sex and gender	The sex of all participants is detailed in the manuscript.
Reporting on race, ethnicity, or other socially relevant groupings	In Sweden and France we are not allowed to ask for race or ethinicity in clinical studies.
Population characteristics	Characteristics of the clinical cohorts are provided in Supplementary Table 1
Recruitment	No recruitment was done for the present study, participants were included as described in the original publications.
Ethics oversight	All studies were approved by the regional ethics boards in Stockholm (cohort 1 and 3) and Paris (cohort 2), and informed written consent was obtained from all study participants. Cohort 1 is part of NCT01727245 and cohort 2 is part of several studies (NCT00476658, NCT01655017, NCT01454232) registered at clinicaltrials.gov.

Note that full information on the approval of the study protocol must also be provided in the manuscript.

Field-specific reporting

Please select the one below that is the best fit for your research. If you are not sure, read the appropriate sections before making your selection.

☒ Life sciences ☐ Behavioural & social sciences ☐ Ecological, evolutionary & environmental sciences

For a reference copy of the document with all sections, see nature.com/documents/nr-reporting-summary-flat.pdf

Life sciences study design

All studies must disclose on these points even when the disclosure is negative.

Sample size	All cohorts included in this study have already been presented previously. No power calculation was performed but the clinical data was reproduced in three independent cohorts from two different countries. The experiments in vitro were repeated at least two-three times using several technical replicates. Finally, for the mouse studies , data are based on at least four animals per group and condition and were reproduced in two different laboratories using orthogonal approaches.
Data exclusions	Possible outliers were identified by “boxplot.stats” with default parameters in R.
Replication	As described above, results were replicated via independent experiments and studies. These included in vitro validation in adipocytes; by in vivo validation utilizing both genetic and pharmacological methodologies; and by analyses in independent clinical cohorts. Furthermore, the reliability and reproducibility of the findings were ensured as all key experiments were independently replicated more than three times, with details regarding the number of independent replications provided in the figure legends.
Randomization	Randomization was not applicable due to the inclusion of samples from participant with diverse medical conditions (i.e with or without obesity) and the analysis of existing data without intervention.
Blinding	The technicians performing the isolation and preparation of the samples were blinded to the group to which the sample belonged.

Reporting for specific materials, systems and methods

We require information from authors about some types of materials, experimental systems and methods used in many studies. Here, indicate whether each material, system or method listed is relevant to your study. If you are not sure if a list item applies to your research, read the appropriate section before selecting a response.

Materials & experimental systems

n/a	Involved in the study
☐	☒ Antibodies
☐	☒ Eukaryotic cell lines
☒	☐ Palaeontology and archaeology
☐	☒ Animals and other organisms
☐	☒ Clinical data
☒	☐ Dual use research of concern
☒	☐ Plants

Methods

n/a	Involved in the study
☒	☐ ChIP-seq
☒	☐ Flow cytometry
☒	☐ MRI-based neuroimaging

Antibodies

Antibodies used	All antibodies are detailed in Supplementary Table 2 including the relevant dilutions.
Validation	All antibodies used in this study are commercially available and have been validated by the respective manufacturer. In addition, we further validated their specificity by western blot showing bands of the expected molecular sizes that were depleted upon gene depletion/knockout or increased upon over expression, respectively.

Eukaryotic cell lines

Policy information about cell lines and Sex and Gender in Research

Cell line source(s)	We use mesenchymal stem cells derived from human white adipose tissue as detailed in the manuscript, see reference 7 in the manuscript. HEK293 cells were purchased from ATCC. Primary murine cells were freshly isolated in the laboratory and immediately utilized for experiments.
Authentication	HEK293 cells were validated by ATCC, while primary cells, including mesenchymal stem cells for efficient adipogenesis and primary adipocytes for adipokine secretion and lipolysis, were authenticated in the laboratory. Authentication primarily relied on morphological characteristics and functional assays (adipokine secretion by ELISA, lipolysis assay).
Mycoplasma contamination	We regularly test for mycoplasma contamination and only use cells that are mycoplasma-free.
Commonly misidentified lines (See ICLAC register)	No commonly misidentified cell lines were used.

Animals and other research organisms

Policy information about studies involving animals; ARRIVE guidelines recommended for reporting animal research, and Sex and Gender in Research

Laboratory animals	C57/Bl6 were used throughout the study. Adiponectin-Cre mice were crossed with Glsfl/fl mice (stock 017894, Glstm2.1Sray/J) to generate adipocyte-specific Gls-depleted mice (GlsAdipoq-Cre). Male mice aged five to fifteen weeks were utilized for the experiments. The mice were group-housed in ventilated cages maintaining a 12-hour light/12-hour dark cycle with lights on from 06:00 to 18:00. The facilities maintained a temperature range of 20-24°C and 50% humidity, with mice having ad libitum access to food and water.
Wild animals	No wild animals were used in the study.
Reporting on sex	Data in male and female mice are presented in the manuscript.
Field-collected samples	No field collected samples were used in the study.
Ethics oversight	Ethical animal board in Stockholm and Paris, respectively

Note that full information on the approval of the study protocol must also be provided in the manuscript.

Clinical data

Policy information about clinical studies

All manuscripts should comply with the ICMJE guidelines for publication of clinical research and a completed CONSORT checklist must be included with all submissions.

Clinical trial registration	Cohort 1 is part of a study registered at clinicaltrials.gov as NCT01727245, and cohort 2 includes samples from studies registered in

Clinical trial registration	the same database under NCT00476658, NCT01655017, NCT01454232. Please note that the metabolomics data from cohort 1 have been published and the relevant references are provided in the paper (refs 7 and 54).
Study protocol	NCT01727245 studied the effect of bariatric surgery in people living with obesity compared with non-obese controls. Only baseline data are provided here.
Data collection	N/a
Outcomes	Outcomes are described under the respective accession numbers at clinicaltrials.gov.

Plants

Seed stocks	N/a
Novel plant genotypes	N/a
Authentication	N/a
