# Supplementary material for: Reduced adipocyte glutaminase activity promotes energy expenditure and metabolic health
Source: Nat Metab. 2024 Jul 15;6(7):1329–46. doi: 10.1038/s42255-024-01083-y (PMC11272588; doi:10.1038/s42255-024-01083-y)
Supplement: Supplementary file 11 — Source western blots in main Figs. 1–8 and Extended Data Figs. 1–7. [file 42255_2024_1083_MOESM11_ESM.pdf]

Uncropped Western Blots

Figure 1e

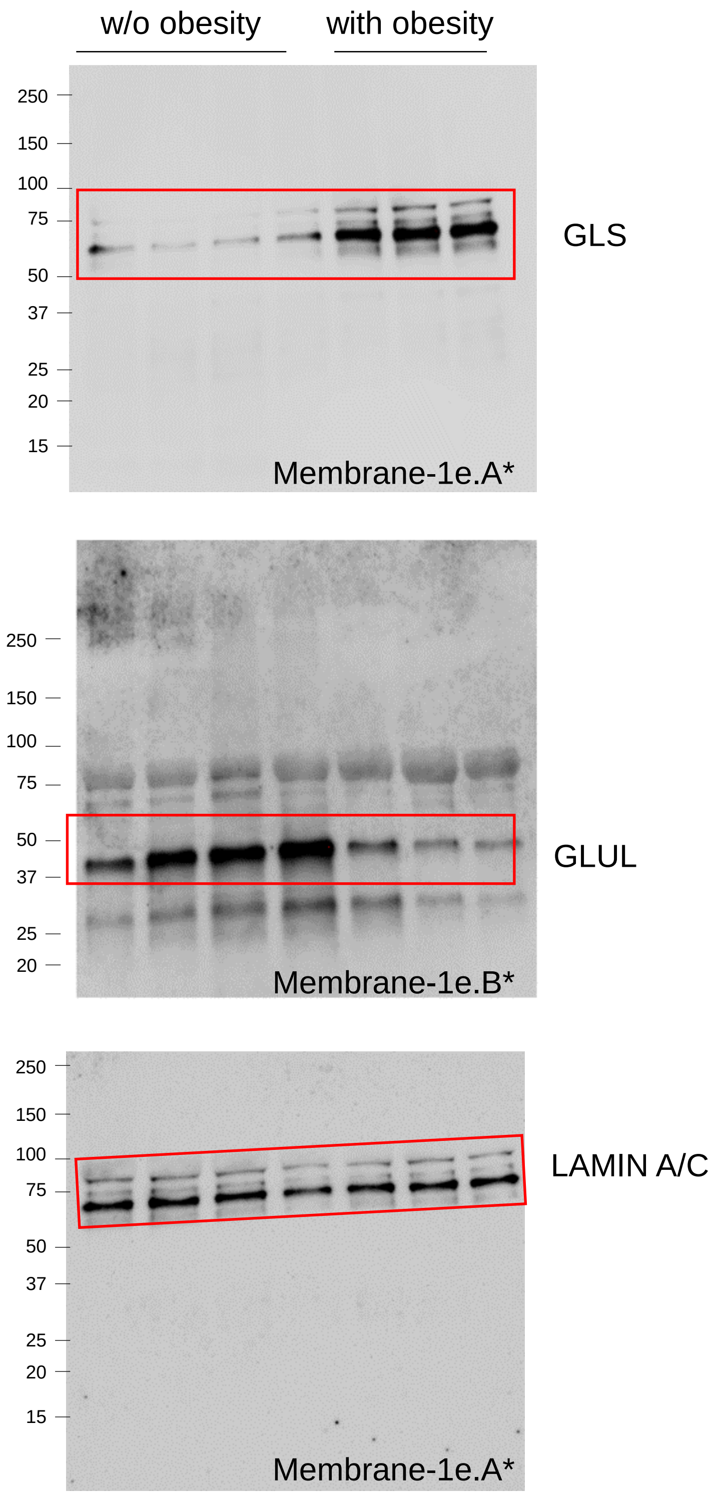

*Lysates were subdivided in equal amounts and loaded on two separate gels.

Uncropped Western Blots

Figure 2a

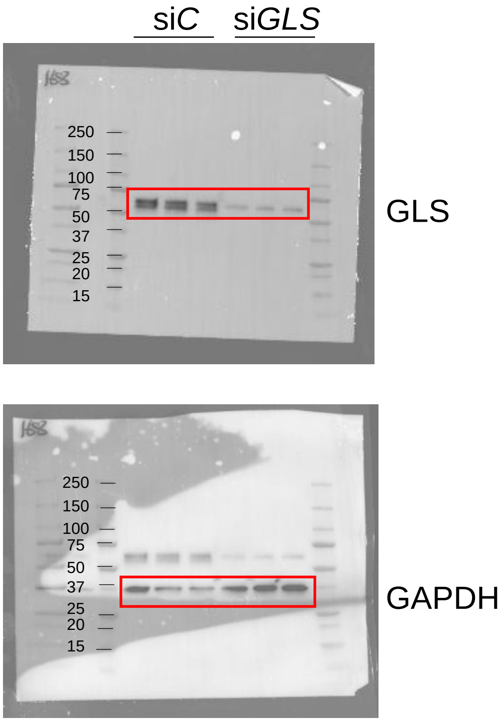

Uncropped Western Blots

Figure 2g

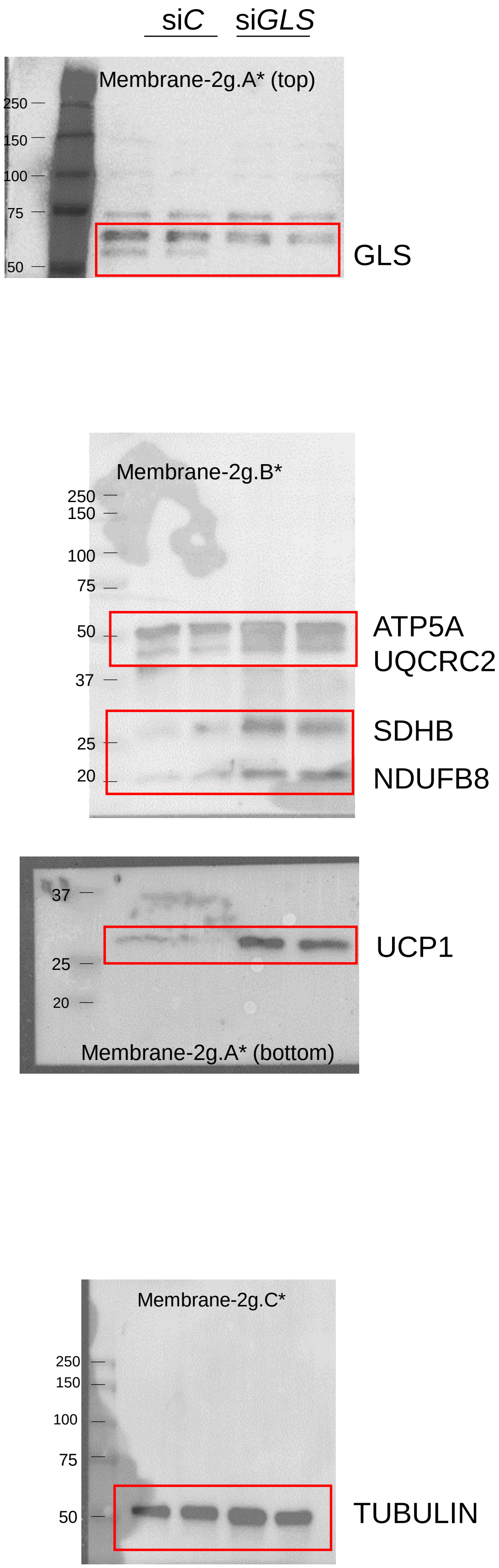

*Lysates were subdivided in equal amounts and loaded on three separate gels.

Uncropped Western Blots

Figure 2i

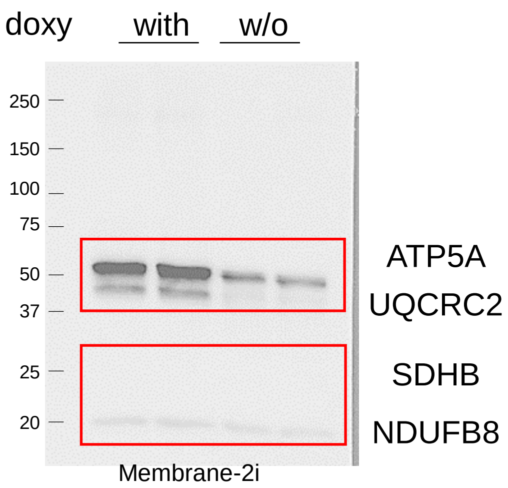

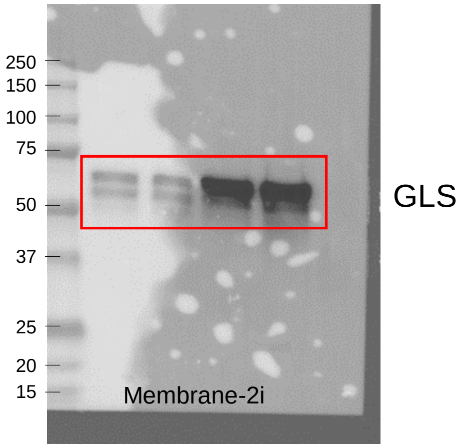

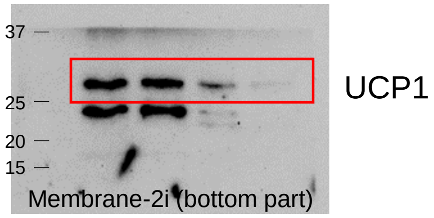

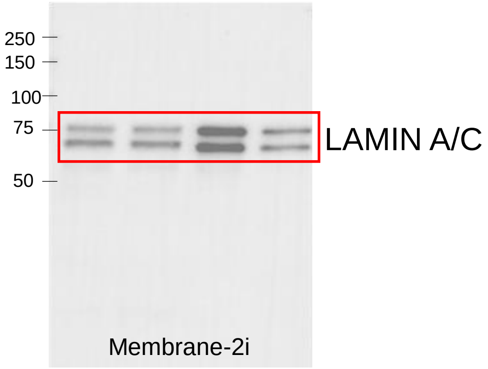

Uncropped Western Blots

Figure 4b

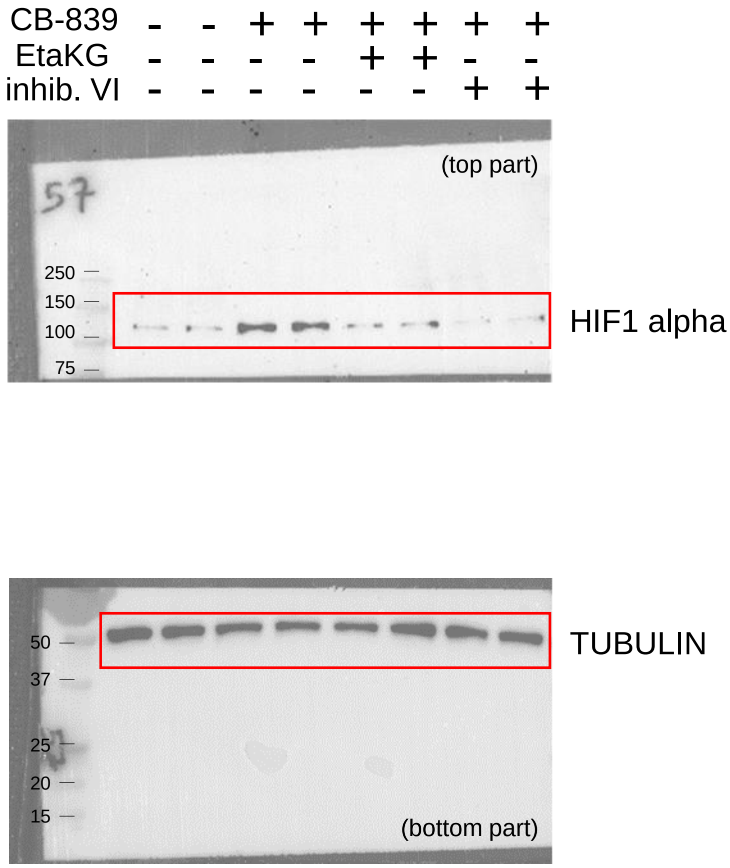

Uncropped Western Blots

Figure 4i

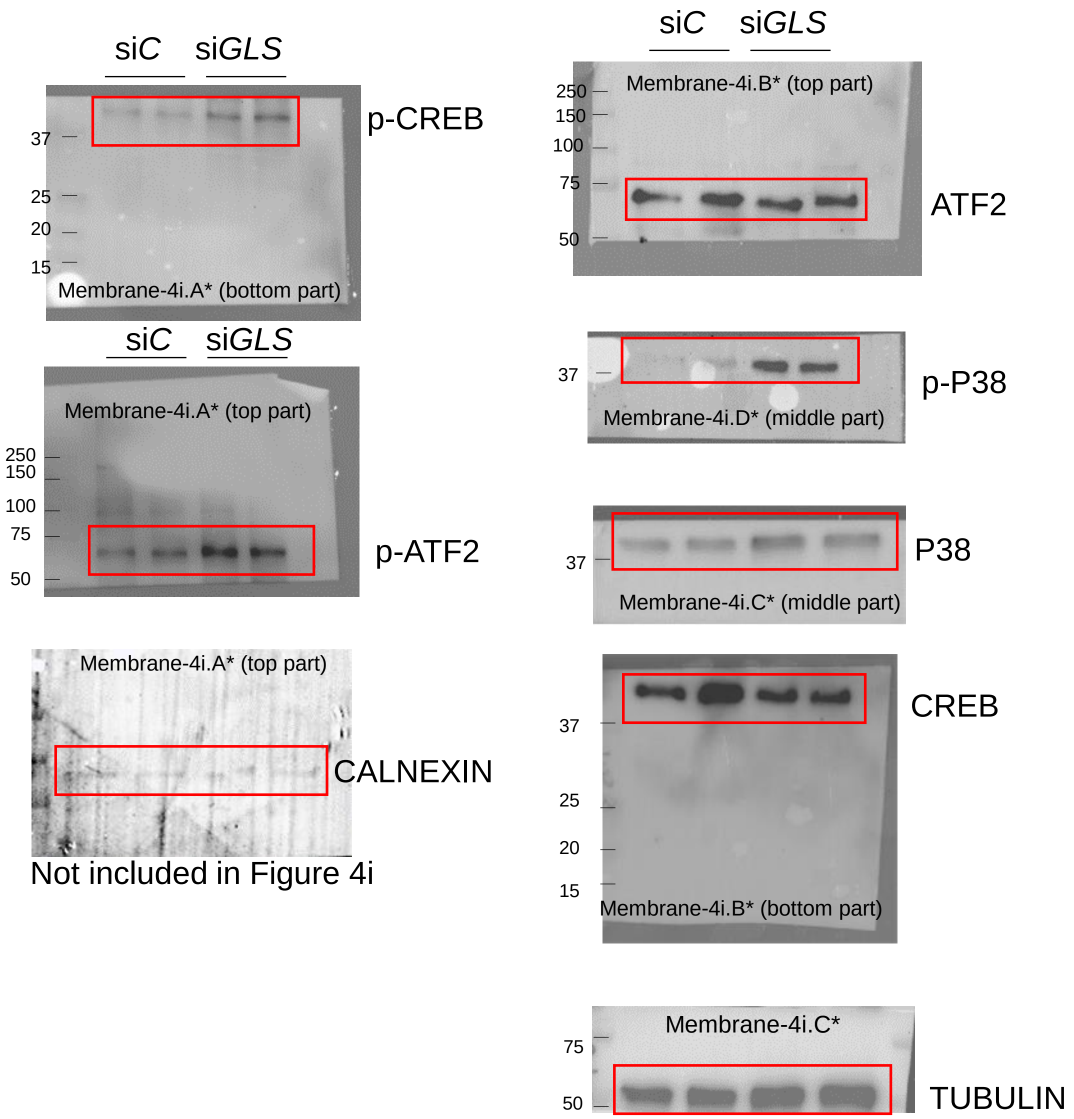

*Lysates were subdivided in equal amounts and loaded on four separate gels.

Uncropped Western Blots

Figure 4j

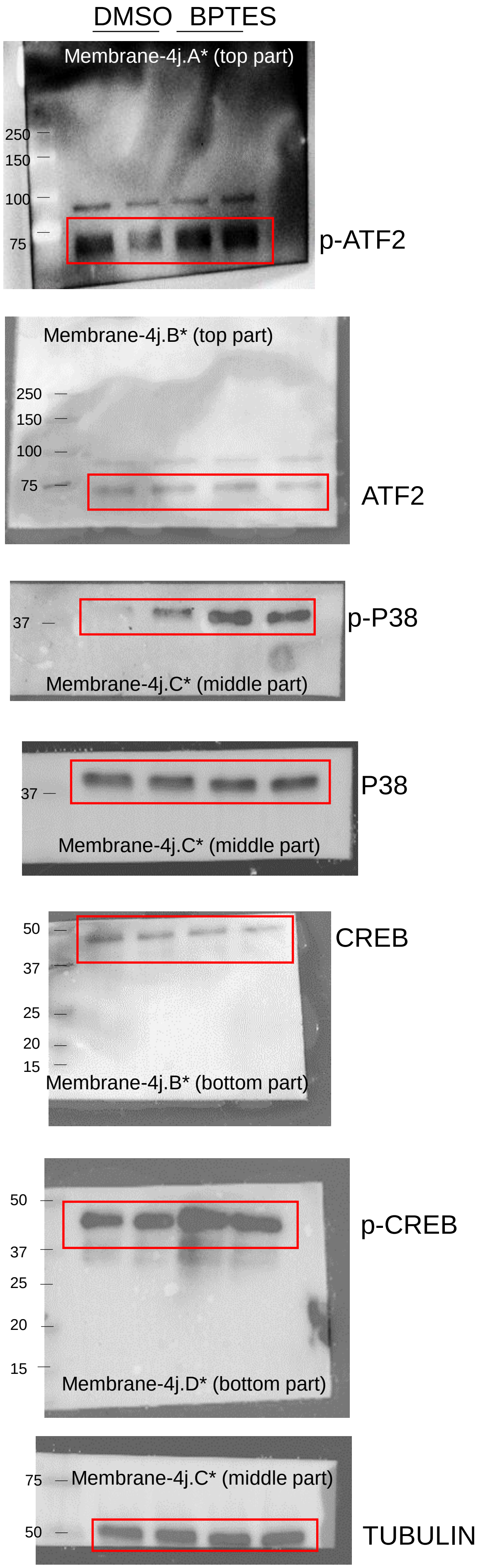

*Lysates were subdivided in equal amounts and loaded on four separate gels.

Uncropped Western Blots

Figure 4k

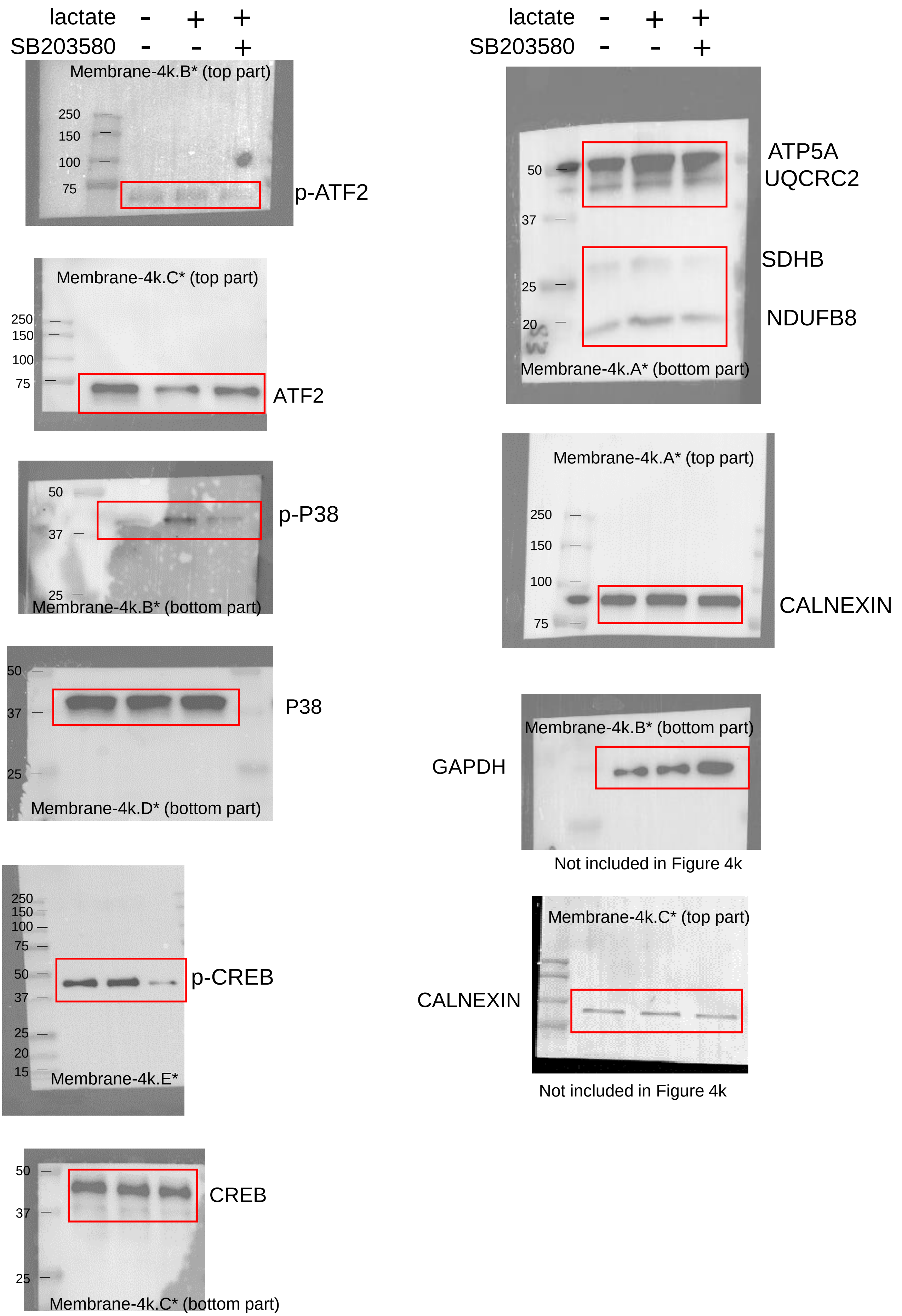

*Lysates were subdivided in equal amounts and loaded on five separate gels.

Uncropped Western Blots

Figure 4l

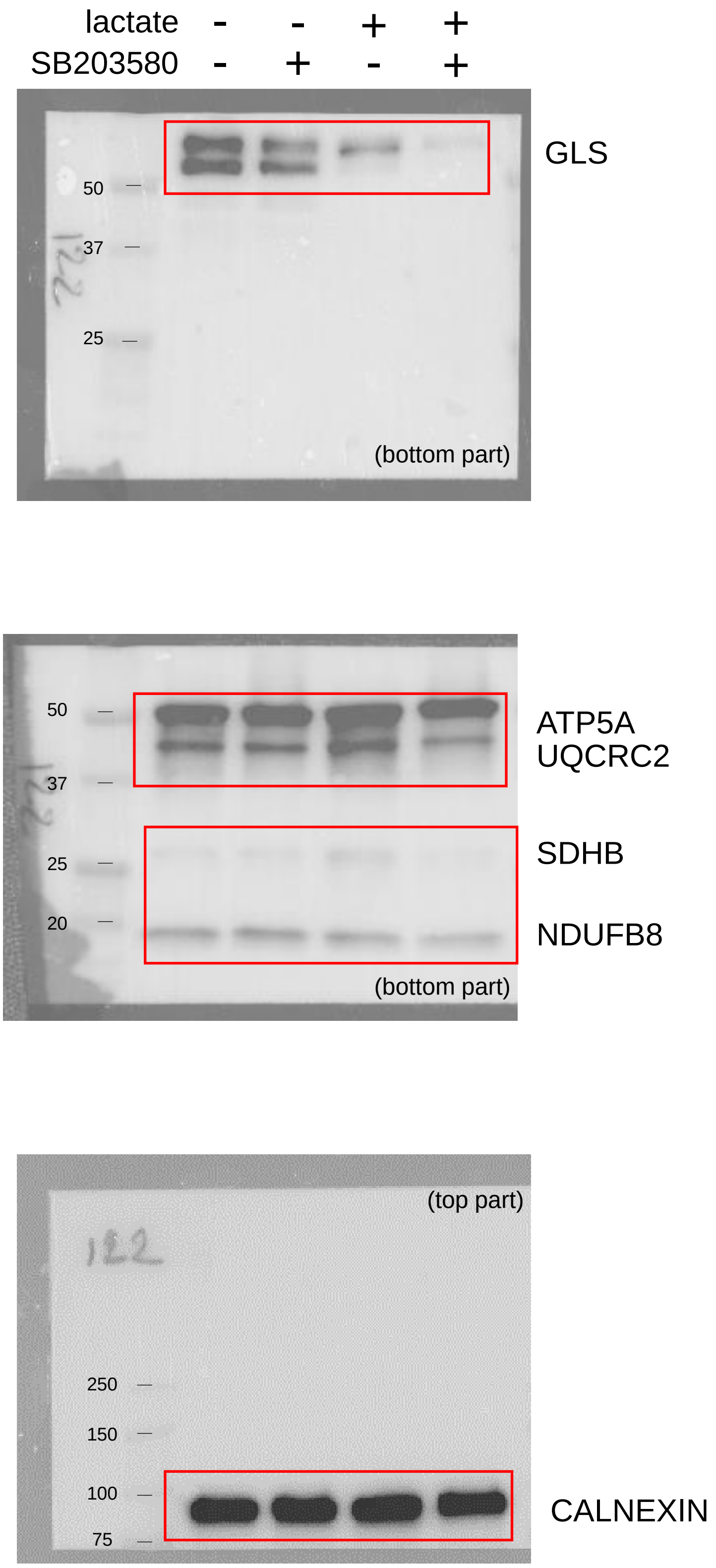

Uncropped Western Blots

Figure 6c

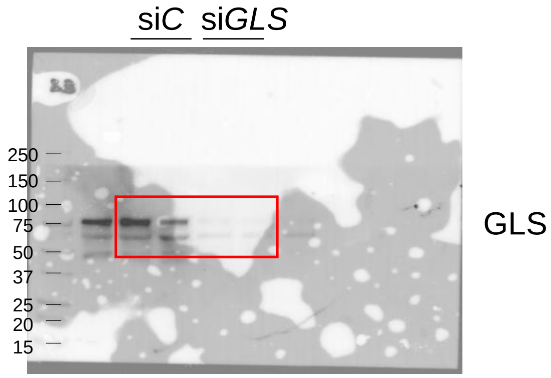

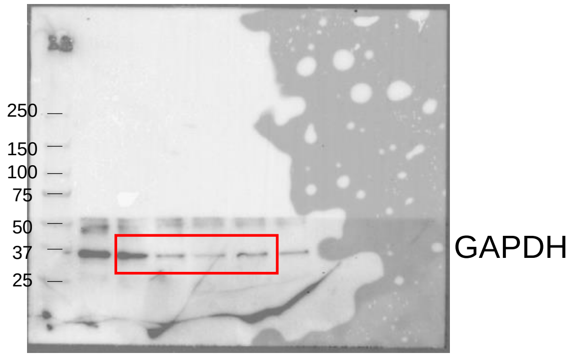

Uncropped Western Blots

Figure 8b

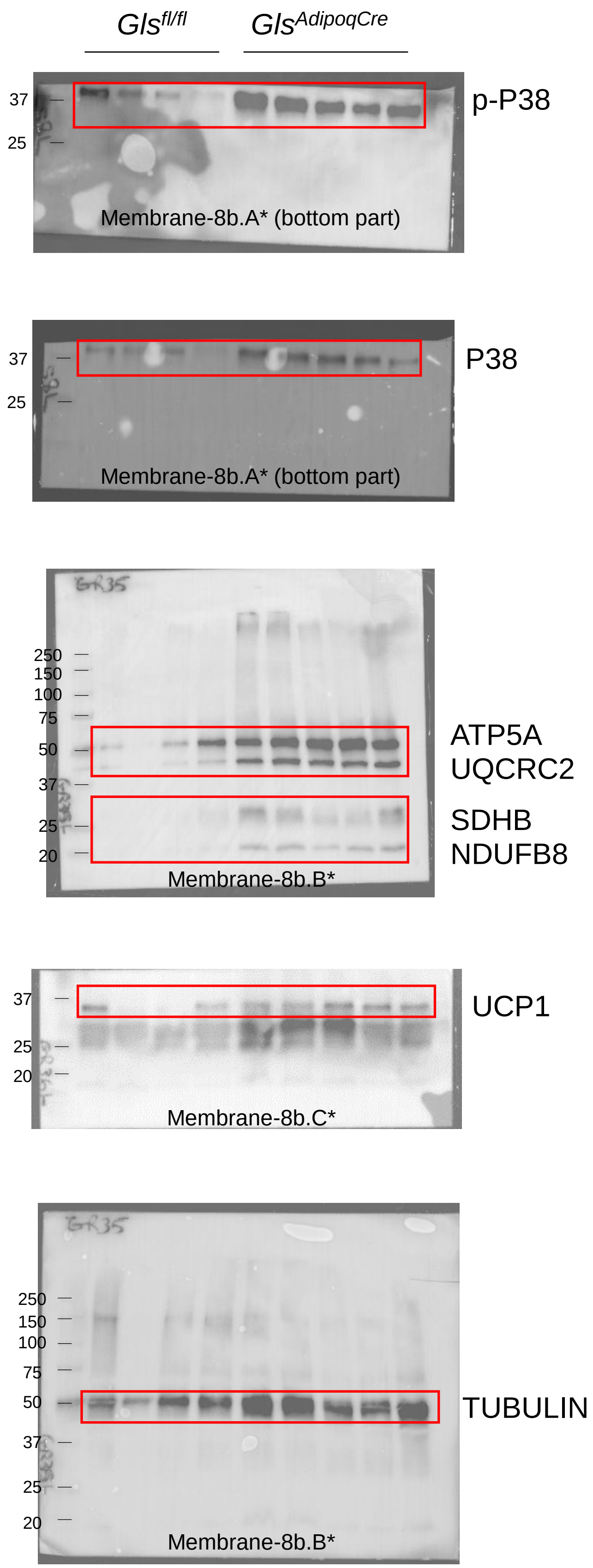

*Lysates were subdivided in equal amounts and loaded on three separate gels.

Uncropped Western Blots

Figure 8i

$Gls^{fl/fl}$ $Gls^{AdipoqCre}$

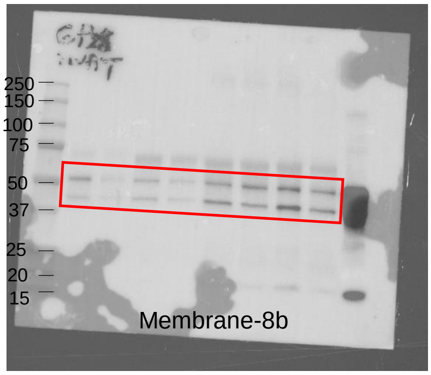

ATP5A
UQCRC2

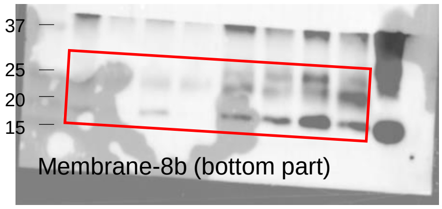

SDHB
NDUFB8

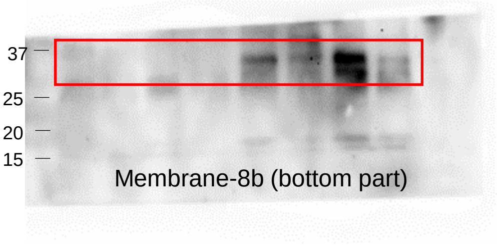

UCP1

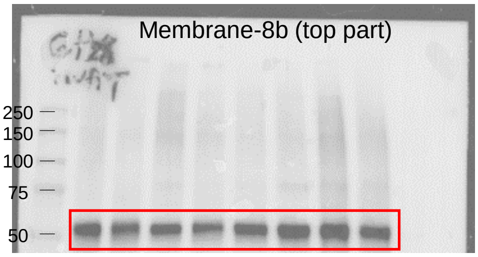

TUBULIN

Uncropped Western Blots

ED Figure 2e

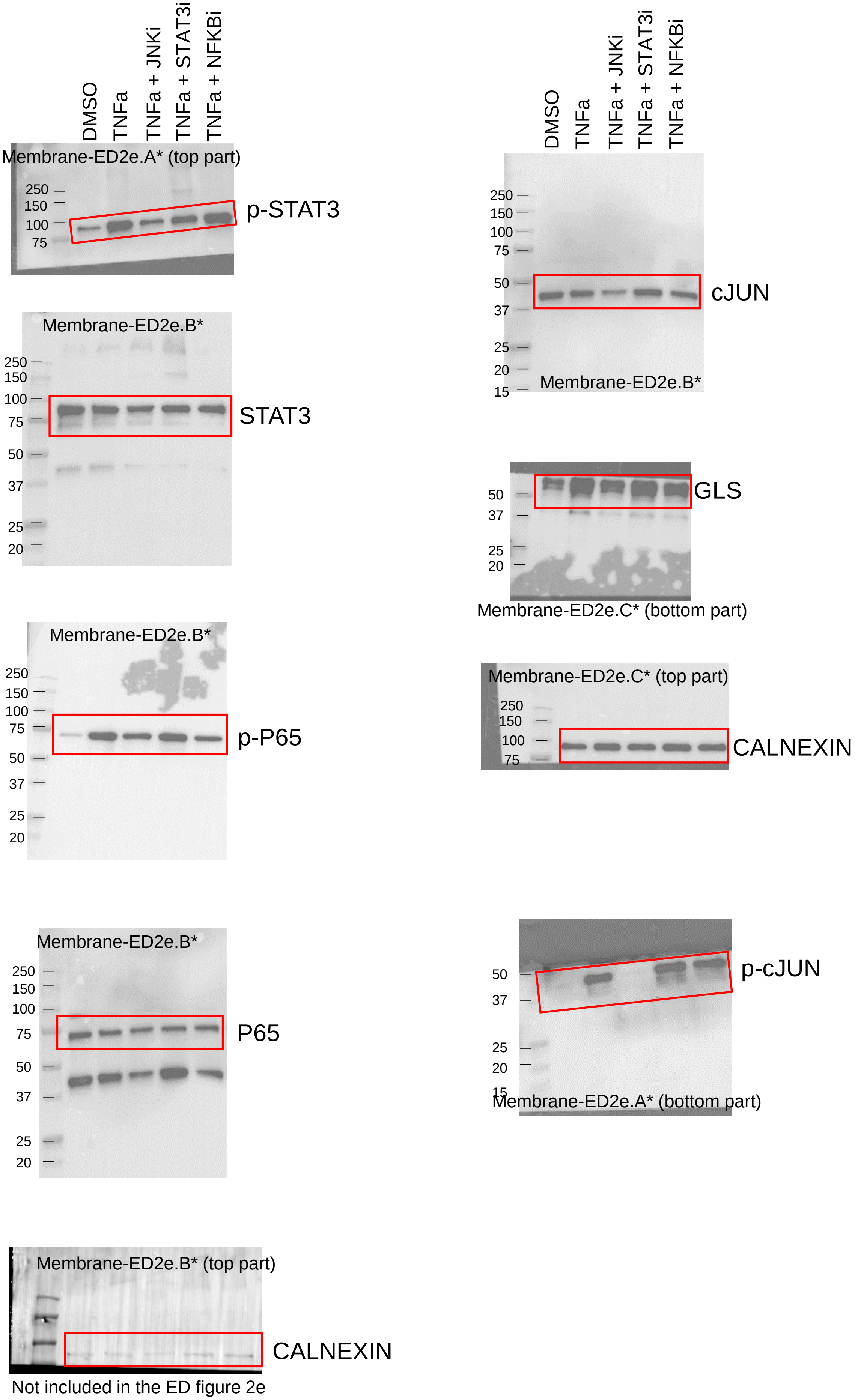

*Lysates were subdivided in equal amounts and loaded on three separate gels.

Uncropped Western Blots

ED Figure 3b

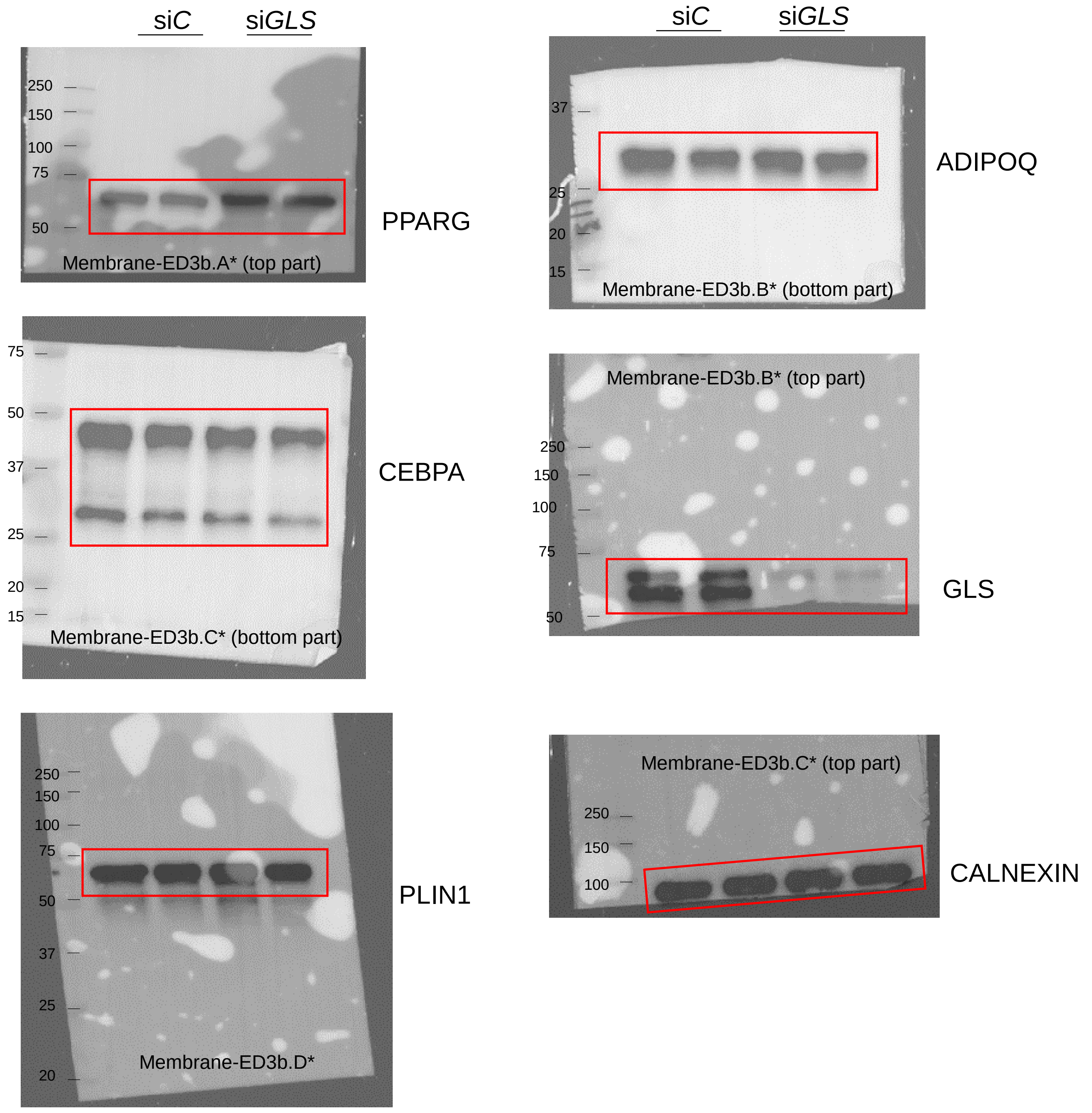

*Lysates were subdivided in equal amounts and loaded on four separate gels.

Uncropped Western Blots

ED Figure 3f

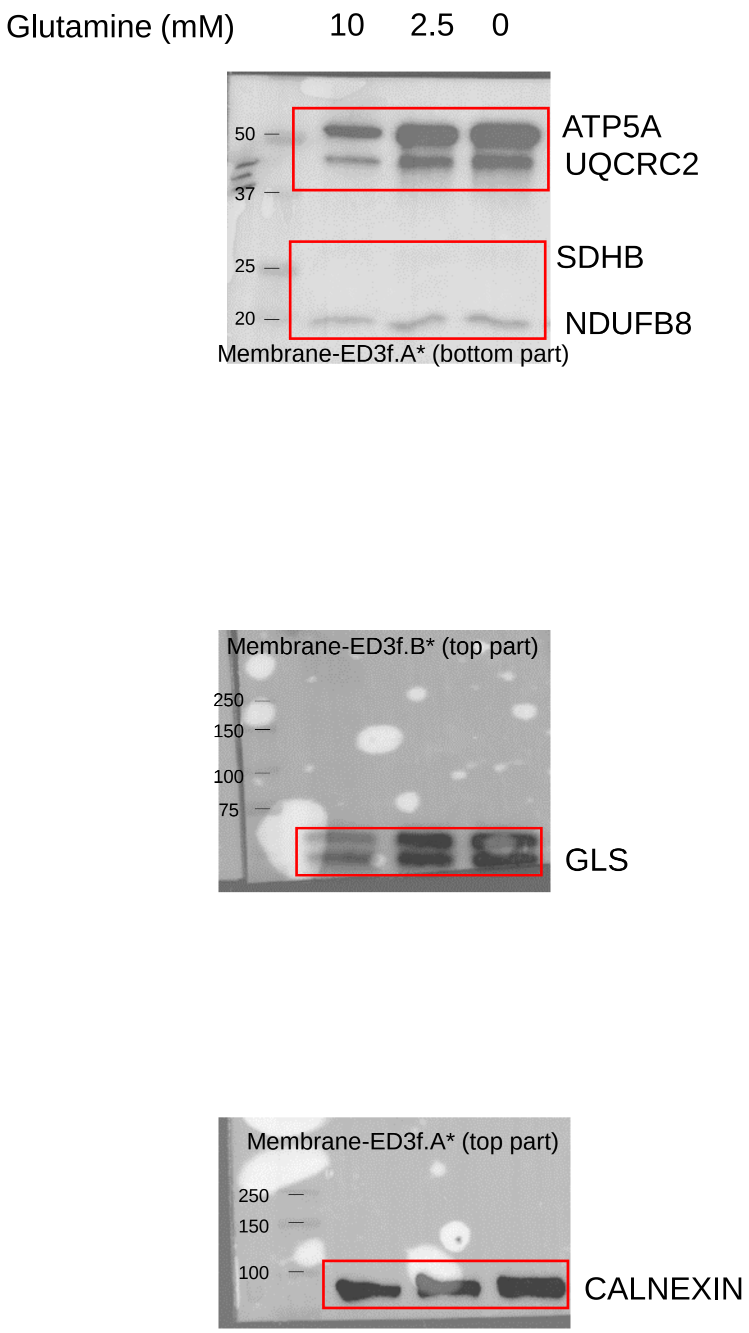

*Lysates were subdivided in equal amounts and loaded on two separate gels.

Uncropped Western Blots

ED Figure 3g

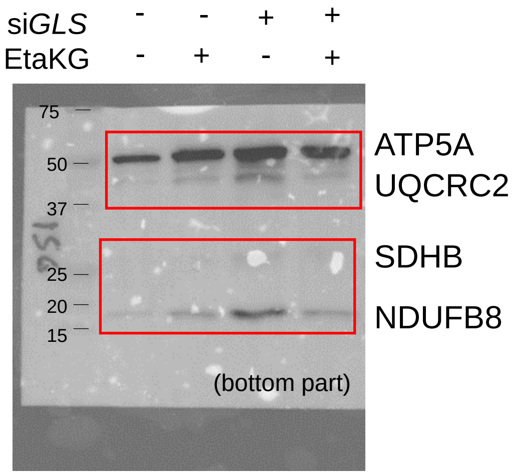

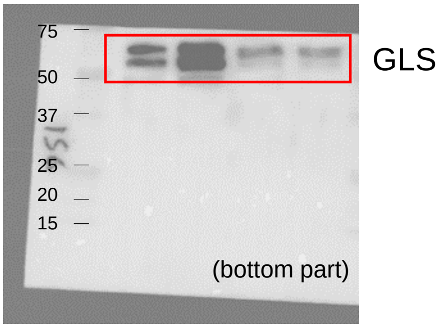

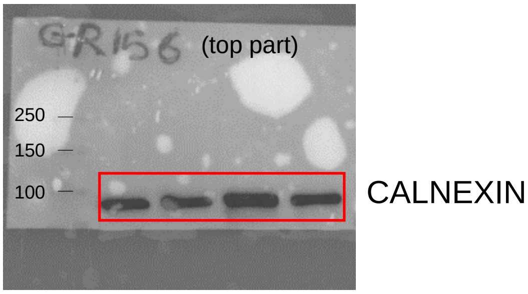

Uncropped Western Blots

ED Figure 3h

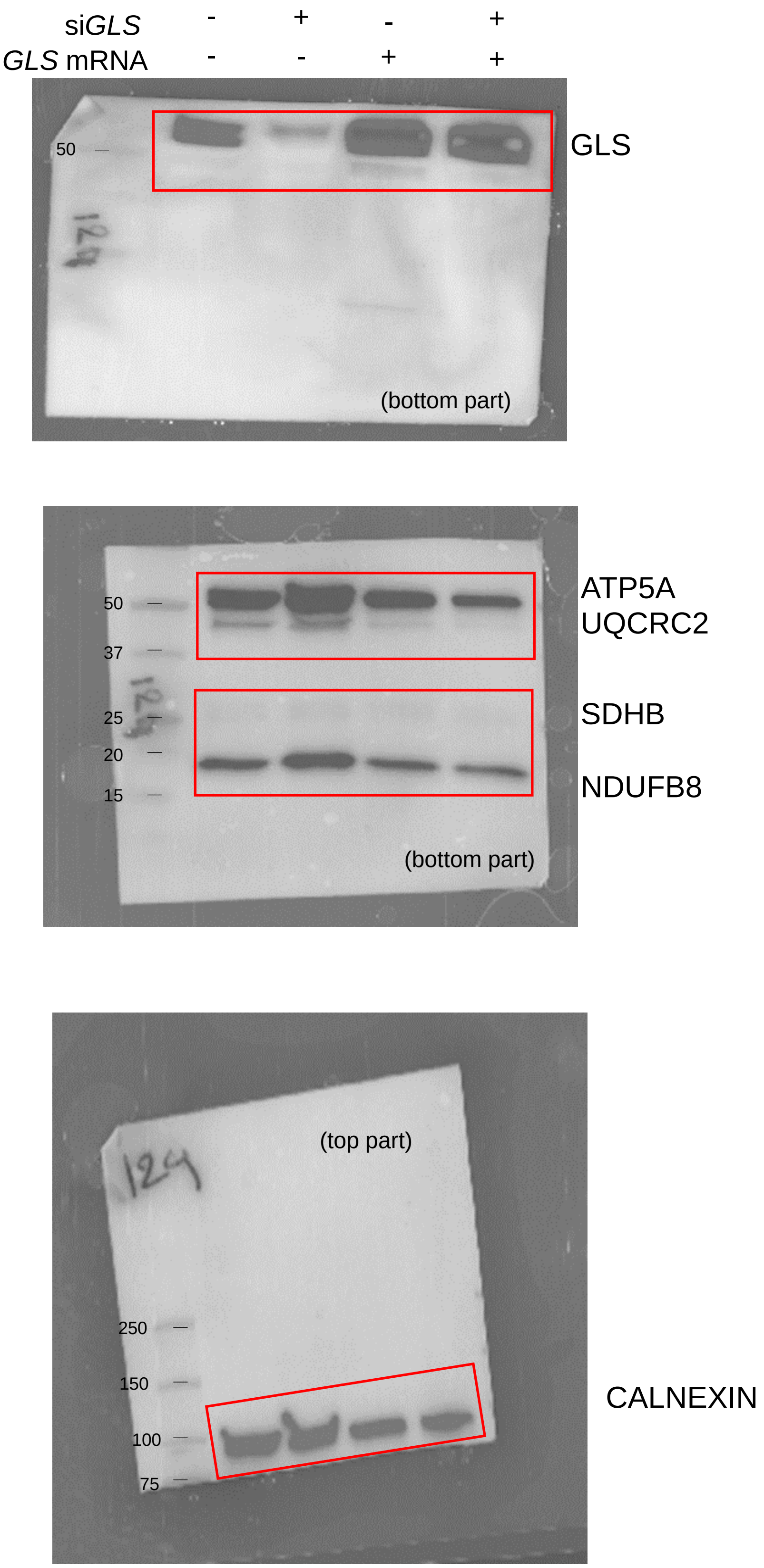

Uncropped Western Blots

ED Figure 5c

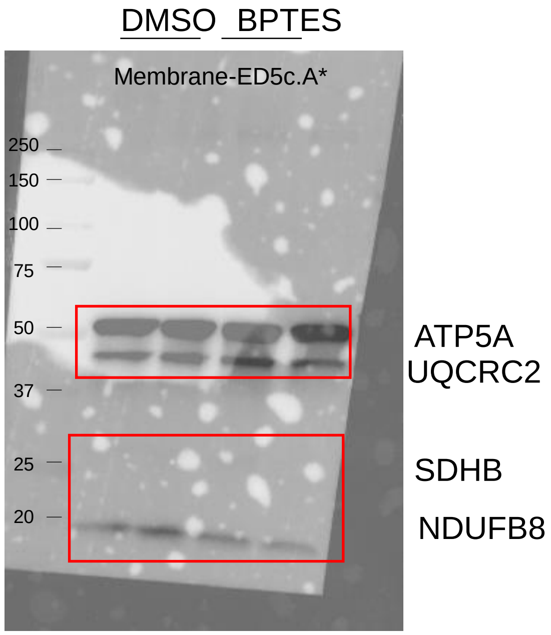

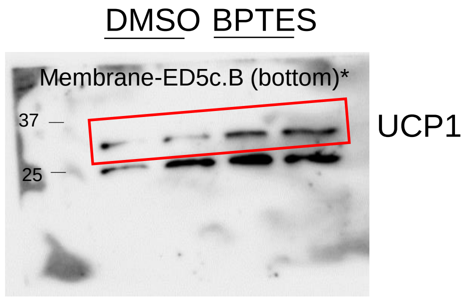

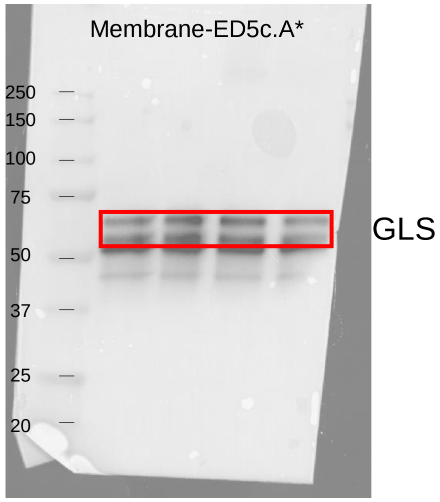

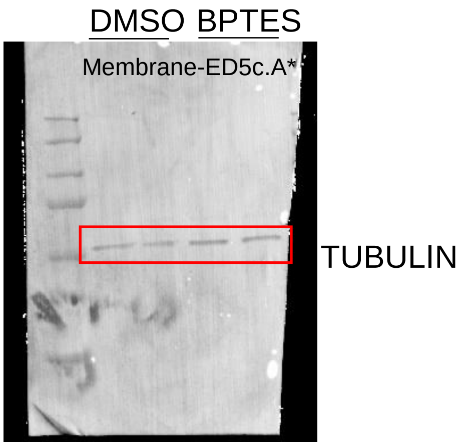

Not included in ED figure 5c

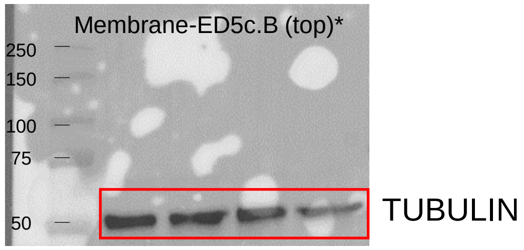

*Lysates were subdivided in equal amounts and loaded on two separate gels.

Uncropped Western Blots

ED Figure 5g

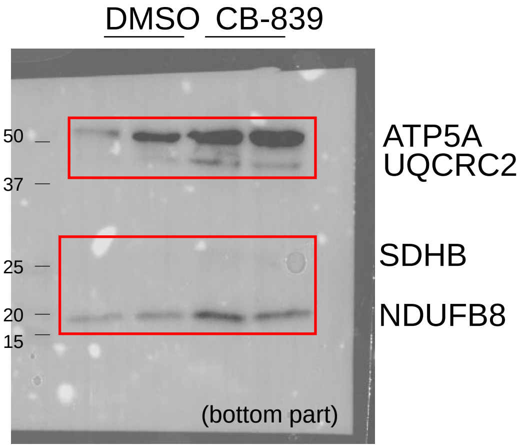

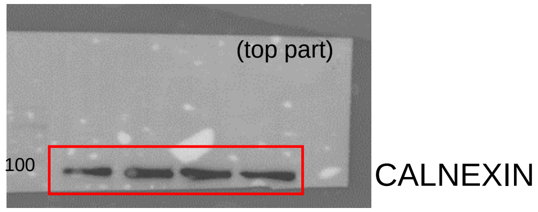

Uncropped Western Blots

ED Figure 6a

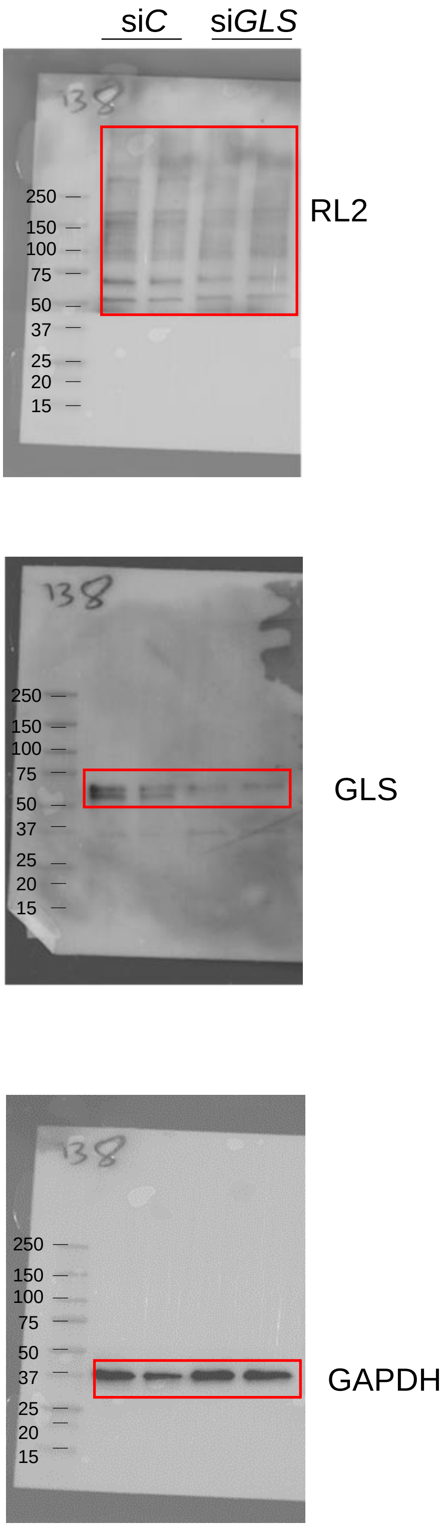

Uncropped Western Blots

ED Figure 6c

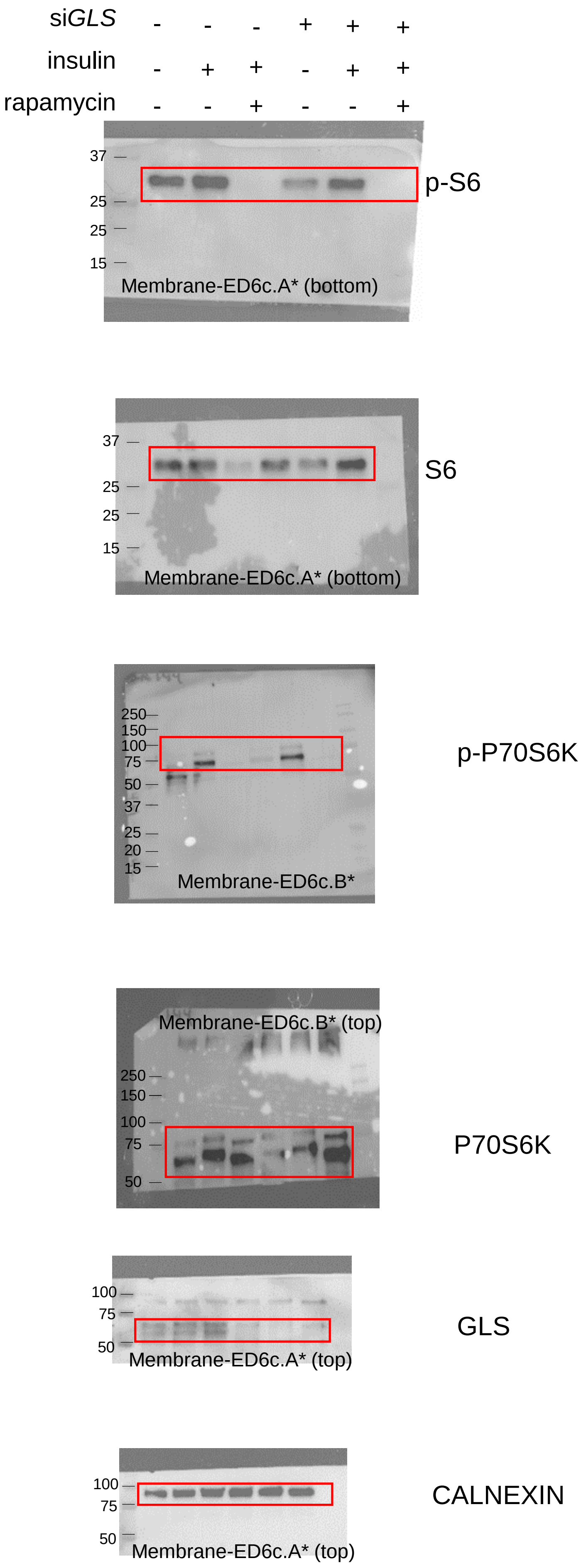

*Lysates were subdivided in equal amounts and loaded on two separate gels.

Uncropped Western Blots

ED Figure 6c – 2nd part

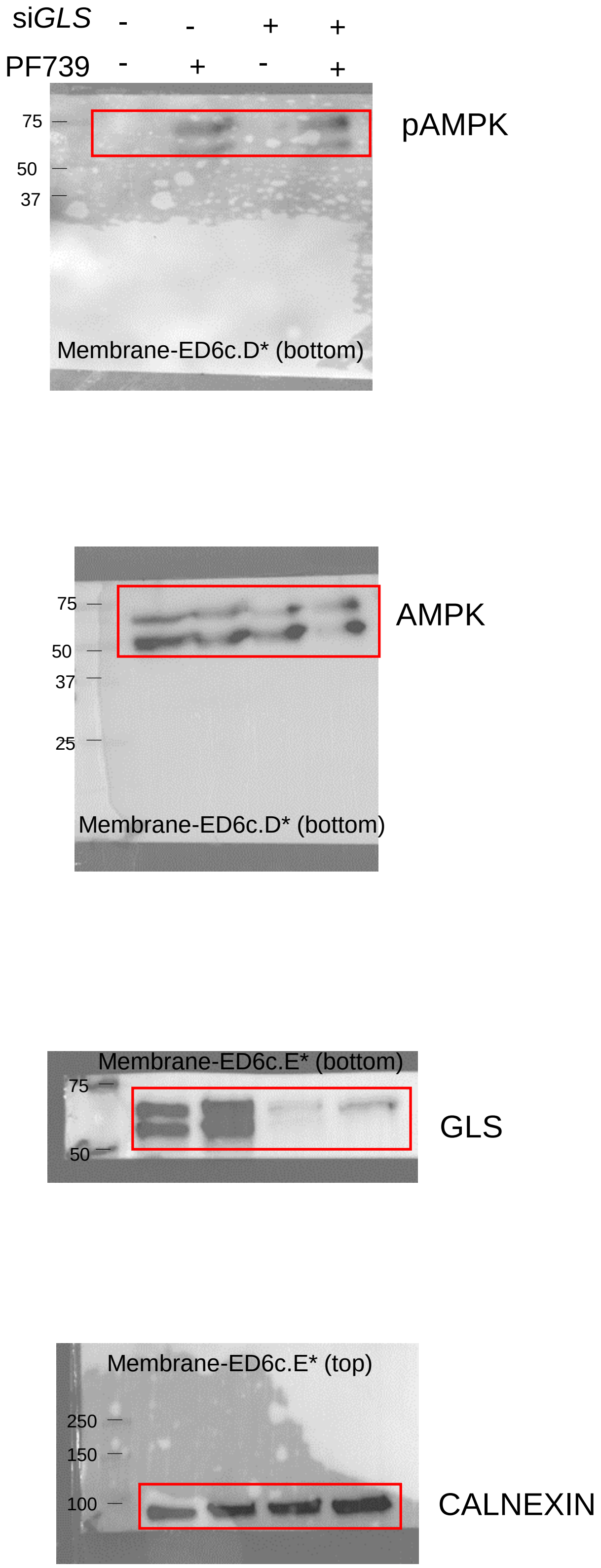

*Lysates were subdivided in equal amounts and loaded on two separate gels.

Uncropped Western Blots

ED Figure 6f

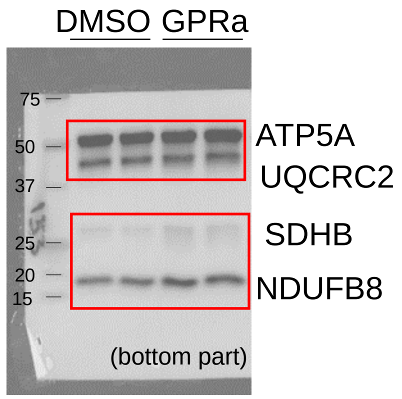

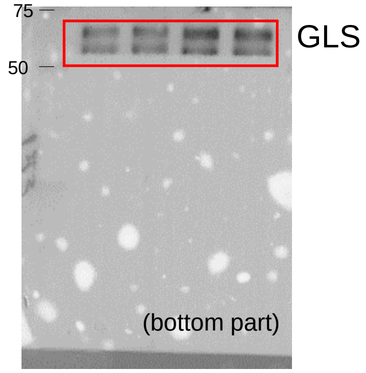

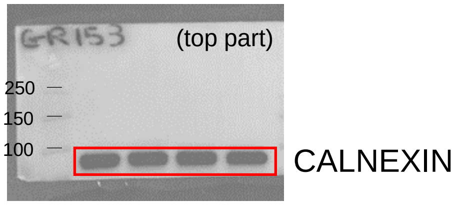

Uncropped Western Blots

ED Figure 6h

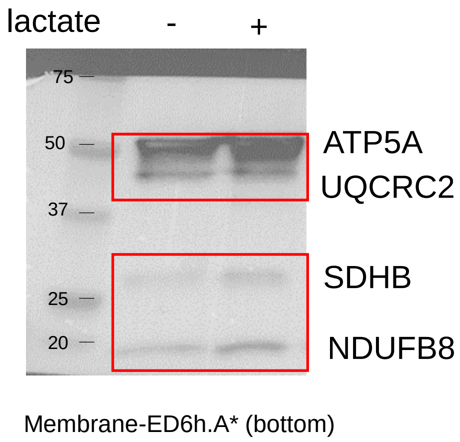

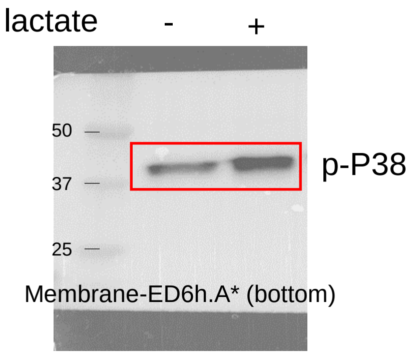

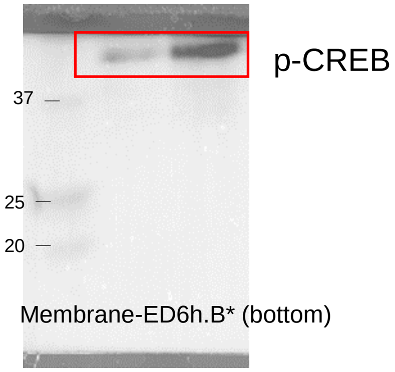

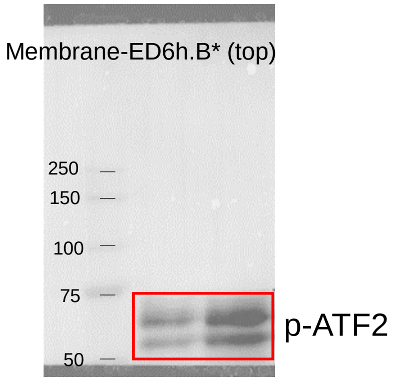

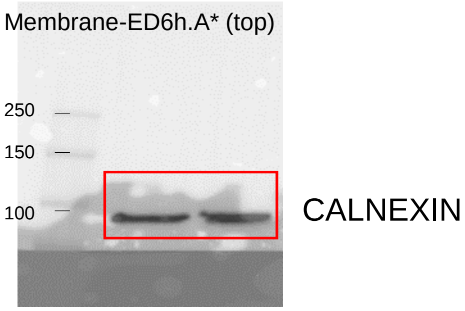

*Lysates were subdivided in equal amounts and loaded on two separate gels.
